# Mercury Isotopes as Proxies to Identify Sources and Environmental Impacts of Mercury in Sphalerites

**DOI:** 10.1038/srep18686

**Published:** 2016-01-05

**Authors:** Runsheng Yin, Xinbin Feng, James P. Hurley, David P. Krabbenhoft, Ryan F. Lepak, Ruizhong Hu, Qian Zhang, Zhonggen Li, Xianwu Bi

**Affiliations:** 1 State Key Laboratory of Environmental Geochemistry, Institute of Geochemistry, Chinese Academy of Sciences, Guiyang 550002, China; 2 Environmental Chemistry and Technology Program, University of Wisconsin-Madison, Madison, WI, 53706, USA; 3 State Key Laboratory of Ore Deposit Geochemistry, Institute of Geochemistry, Chinese Academy of Sciences, Guiyang 550002, China; 4 Department of Civil and Environmental Engineering, University of Wisconsin-Madison, Madison, WI, 53706, USA; 5 U.S. Geological Survey, 8505 Research Way, Middleton, WI, 53562, USA

## Abstract

During the past few years, evidence of mass independent fractionation (MIF) for mercury (Hg) isotopes have been reported in the Earth’s surface reservoirs, mainly assumed to be formed during photochemical processes. However, the magnitude of Hg-MIF in interior pools of the crust is largely unknown. Here, we reported significant variation in Hg-MIF signature (Δ^199^Hg: −0.24 ~ + 0.18‰) in sphalerites collected from 102 zinc (Zn) deposits in China, indicating that Hg-MIF can be recorded into the Earth’s crust during geological recycling of crustal material. Changing magnitudes of Hg-MIF signals were observed in Zn deposits with different formations, evidence that Hg isotopes (especially Hg-MIF) can be a useful tracer to identify sources (syngenetic and epigenetic) of Hg in mineral deposits. The average isotopic composition in studied sphalerites (*δ*^*202*^*Hg*_*average*_: −0.58‰; Δ^*199*^*Hg*_*average*_: +0.03‰) may be used to fingerprint Zn smelting activities, one of the largest global Hg emission sources.

Mercury (Hg) is a photochemically active, redox-sensitive metal and exists as multiple physical states in the environment^1^. It has seven natural stable isotopes (196, 198, 199, 200, 201, 202 and 204) with a relative mass span of 4%. Recently, multi-collector inductively coupled plasma mass spectrometry (MC-ICP-MS) has enabled very high precision to quantify small differences in Hg isotopic ratios (< ± 0.1‰)^2^,^3^. With the recent discovery that Hg can exhibit both mass-dependent (MDF, expressed as δ^202^Hg) and mass-independent (MIF, expressed as Δ^199^Hg) isotope fractionation, Hg isotopes can provide multi-dimensional tracers to discriminate sources, transport, transformation and bioaccumulation of Hg in the environment^4^,^5^,^6^,^7^. Hg-MDF, which is induced by differences in zero-point energy of different isotopes masses, can occur during various physical, chemical and biological processes^4^,^5^,^6^,^7^. Hg-MIF of odd Hg isotopes (^199^Hg and ^201^Hg), mainly caused by the nuclear volume effect (NVE)^8^ and magnetic isotope effect (MIE)^9^, can give additional information on specific processes such as elemental Hg(0) volatilization^10^,^11^, equilibrium Hg-thiol complexation^12^, dark Hg(II) reduction^13^ and photochemical processes^13^,^14^,^15^,^16^,^17^. Signatures of both Hg-MDF and Hg-MIF, often of very large magnitude (δ^202^Hg and Δ^199^Hg: >10‰), have been reported in natural samples^4^,^5^,^6^,^7^.

Previous studies reported changing magnitudes of Hg-MIF in natural samples which are mainly located in the Earth’s surface (e.g., soil, sediment, peat, water, atmosphere and biological samples) and near surface environment (e.g., coal, black shale – Fig. 1). In contrast, syngenetic (e.g. mantle-derived) Hg source has shown the absence of Hg-MIF (Δ^199^Hg ~ 0)^18^. Photochemical reactions have been implicated as the main processes to generate Hg-MIF in the environment^4^,^5^,^6^,^7^. Photo-reduction of Hg(II) and photo-degradation of methylmercury (MeHg), driven by dissolved organic matter (DOM), produce Δ^199^Hg/Δ^201^Hg of approximately 1 and 1.3 (ref. 14), respectively, which is in accordance with the Δ^199^Hg/Δ^201^Hg reported in natural samples (Fig. 2).

The atmosphere, biosphere and the crust are all interconnected, and the interactions between tectonic and hydrologic systems cause constant recycling of the Earth’s crustal materials^19^. This includes transport of surface materials to the interior crust followed by heating, metamorphosis, melting, lithification and weathering^19^. During these processes, it is possible that Hg-MIF may leave a record in the interior of the crust. However, the magnitudes of Hg-MIF in Hg pools of the interior crust have been largely unexplored. Because Hg is a toxic pollutant, most studies on Hg isotope geochemistry have been focused on the Earth’s surface environment-the critical zone for humans and wildlife^4^,^5^,^6^,^7^. Only a few studies reported a small extent of Hg-MIF in crustal rocks^4^,^20^, hydrothermal ores^20^,^21^,^22^,^23^,^24^ while mantle-derived materials^18^ have almost been ignored.

Sulphide mineral deposits are the most important Hg pool in the Earth’s crust^25^. Due to the chalcophilic nature of its associations, Hg is found in abundance in hydrothermal deposits of sulphide minerals [e.g. cinnabar (HgS), sphalerite (ZnS), etc]^25^. Both Hg and zinc (Zn) belong to the IIB group in the element periodic table, and Hg has a close geochemical relationship with Zn^26^. The presence of anomalous concentrations of Hg have been observed in sphalerites^22^,^25^,^26^,^27^, the most abundant form of Zn in hydrothermal deposits^28^,^29^,^30^,^31^. Extraction of Zn from sphalerites has received broad concerns due to the fact that Zn smelting is regarded as one of the largest anthropogenic Hg emission sources to the atmosphere^26^,^32^,^33^. From an economic geology viewpoint^26^,^28^,^29^,^30^,^31^, four main formations of Zn deposits are categorized: sedimentary exhalative deposits (SEDEX), Mississippi Valley type (MVT), volcanic hosted massive sulphides (VMS) and intrusion related deposits (IR). Both SEDEX and MVT deposits formed from formation waters derived from sedimentary basins with high heat flows, which are characterized by the lack of igneous rocks^26^,^28^,^29^,^31^. The main difference between SEDEX and MVT deposits is in their depositional settings. SEDEX deposits form at or just below the seafloor^22^,^29^,^31^, whereas MVT deposits form in open spaces within carbonate platformal sequences^22^,^28^,^31^. VMS and IR deposits are common to igneous rocks, and have shown to be largely related to deep-seated intrusions of magmatic materials^26^,^30^,^31^,^34^. VMS deposits are mainly located in submarine divergent margins^26^,^30^,^31^, whereas IR deposits are typically found in carbonate rocks in conjunction with magmatic systems^26^,^31^,^34^. The total Hg concentration (THg) in sphalerites is highly variable, mainly controlled by deposit formations^26^. Changes in formation of Zn deposits indicate that sphalerites may be an important formation to investigate variations of Hg-MIF in deep geological settings. Meanwhile, knowing the Hg isotopic composition in sphalerites is essential to evaluating its environmental impact, including as a source signature of Hg emission from Zn smelting.

To date, only one study reported Hg isotopic distribution in sphalerites collected from Zn deposits worldwide^22^. Even though very limited number of samples (n = 7) were investigated, this study reported large variations of δ^202^Hg (−1.41 to +0.46‰) and minor Hg-MIF (Δ^199^Hg: −0.12 to +0.05‰)^22^. China has rich Zn resources and its Zn reserve ranks the second in the world^35^. In this study, sphalerites collected from 102 Zn deposits in China were measured for Hg isotopic compositions. Our data (Supplementary Table S2) show large ranges of δ^202^Hg (−1.87 to +0.70‰, n = 102) and Δ^199^Hg (range: −0.24 to +0.18‰, n = 102). The overall range of Δ^199^Hg are more than twice that reported by previous studies (Δ^199^Hg: −0.12 to +0.05‰, n = 7)^22^.

## Mass dependent fractionation signature of Hg

Previous studies on hydrothermal ore deposit samples have reported a large range of δ^202^Hg values, attributable to MDF during vapor phase transport and venting of hydrothermal fluids^18^,^20^,^21^. Similar processes are likely responsible for the observed variability in δ^202^Hg values (−1.87 to +0.70‰, n = 102) in sphalerites. No clear correlation and spatial distribution patterns were observed for δ^202^Hg, THg and Δ^199^Hg values. Given the intra-deposit variations of Hg (concentrations and isotopic compositions) and the limited sample size from each deposit, it remains unclear whether the variations of δ^202^Hg in sphalerites is mainly a result of Hg(0) volatilization. More detailed studies focused on a certain deposit are needed in the future.

Samples investigated in this study show an overall mean δ^202^Hg of −0.47 ± 0.93‰ (2σ, n = 102), similar to previous data on sphalerites (mean δ^202^Hg: −0.76 ± 0.62‰, σ, n = 7)^22^. ANOVA tests for δ^202^Hg values among MVT, SEDEX, VMS and IR deposits showed *P* values range from 0.32 to 0.78, indicating no statistically significant differences. The mean δ^202^Hg values for MVT (mean: −0.65 ± 0.65‰, σ, n = 25), SEDEX (mean: −0.57 ± 0.40‰, σ, n = 19), VMS (mean: −0.52 ± 0.24‰, σ, n = 14) and IR (mean: −0.32 ± 0.38‰, σ, n = 44) deposits are similar to previous data on Hg ore deposits. For instance, Smith and co-authors^20^,^21^ demonstrated a mean δ^202^Hg of −0.64 ± 0.96‰ (σ, n = 112) for Hg ore deposits from the California Coast Ranges and Nevada. Blum and Bergquist^2^ reported a δ^202^Hg value of −0.54‰ for the world’s historically largest Hg mine (Almadén, Spain), and Yin *et al.*^36^ reported a similar mean δ^202^Hg (−0.74 ± 0.11‰, σ, n = 14) for world’s third largest Hg mine (Wanshan, China). Syngenetic and epigenetic Hg are the two primary sources of Hg in hydrothermal deposits^37^,^38^. Syngenetic Hg enters the crust through volcanoes, hot spots, and oceanic spreading centres^18^. Values of syngenetic δ^202^Hg (mean: −0.23 ± 0.19‰, σ, n = 3) have been reported for vent chimneys from the Guaymas Basin sea-floor rift, USA^18^. Epigenetic Hg originally comes from syngenetic Hg, whereas it has undergone biogeochemical cycling in the surface environment (e.g. emission, long-range transport and deposition), and re-entered the crust through sediment diagenesis processes^37^,^38^. Large variations of δ^202^Hg (>10‰) have been reported in surface reservoirs (e.g., atmospheric, soils, sediments), whereas epigenetic Hg in sedimentary rock units in California Coast Ranges revealed relatively narrow δ^202^Hg ranges (−0.93 to −0.17‰) with a mean value of −0.63 ± 0.24‰ (σ, n = 15)^20^, suggestive that epigenetic Hg is a mixture of Hg from surface reservoirs. Hydrothermal fluids percolate through crustal rocks which can leach, concentrate, and transport both syngenetic and epigenetic Hg^18^,^37^,^38^, and may be the reason for similar mean δ^202^Hg values between Zn and Hg ore deposits.

## Mass independent fractionation signature of Hg

The overall range of 0.42% in Δ^199^Hg values in our samples is surprisingly large, being an order of magnitude higher than the analytical uncertainty for UM-Almadén (±0.04‰, 2σ). Even though some sphalerites showed large uncertainties of Δ^199^Hg (up to ±0.10‰, 2σ), possibly reflective of the heterogeneity of Hg in the samples, 83% of the samples have uncertainties within ±0.04‰ (2σ). Hg-MIF has been shown to be induced by MIE^9^ during photoreduction of aqueous Hg(II) and photo-degradation of MeHg processes^14^,^15^,^16^,^17^. Other processes [e.g., elemental Hg(0) volatilization^10^,^11^, equilibrium Hg-thiol complexation^12^, dark Hg(II) reduction^13^] have also been shown to generate Hg-MIF, which has been mainly explained by the NVE^8^. Among the various processes, photochemical reactions may be of greatest importance in observed MIF, as these reactions typically generate the largest Hg-MIF. Other processes produce Hg-MIF of almost one order of magnitude lower^5^,^6^,^14^. The ∆^199^Hg/∆^201^Hg of 0.93 ± 0.09 (2σ) for the sphalerites (Fig. 2) is consistent with the aqueous Hg(II) photo-reduction reported by Bergquist and Blum^14^, suggesting that Hg-MIF in sphalerites may be caused by aqueous Hg(II) photo-reductions. Other processes which show ∆^199^Hg/∆^201^Hg of 1.5 to 2.0^10^,^11^,^12^,^13^, cannot explain the Hg-MIF observed in study (∆^199^Hg/∆^201^Hg of ~1).

## Use of Hg-MIF to trace metal sources in different types of Zn deposits

**Figure 1:**
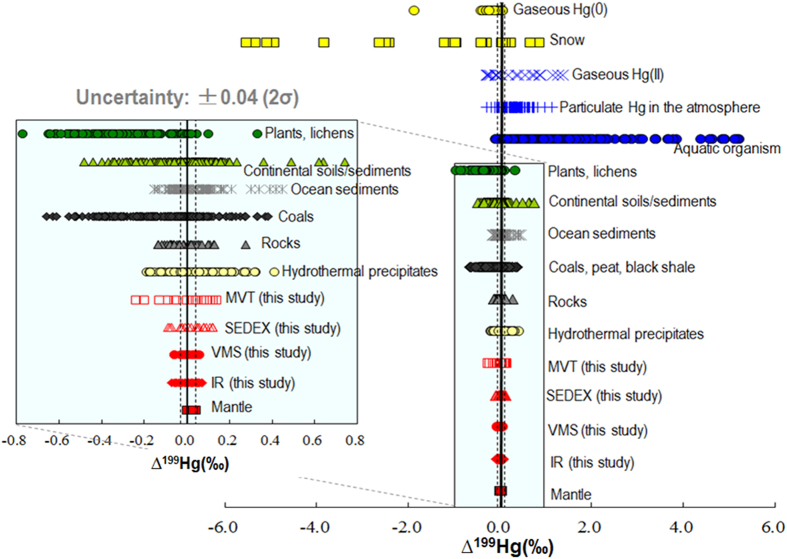
Variations of ∆^199^Hg in different environmental samples (based on previously published data summarized in Table S1) and sphalerites (this study). Black solid line indicates ∆^199^Hg of 0, which represent no Hg-MIF. Gray dot lines represent the analytical uncertainty (∆^199^Hg: ±0.04‰).

**Figure 2:**
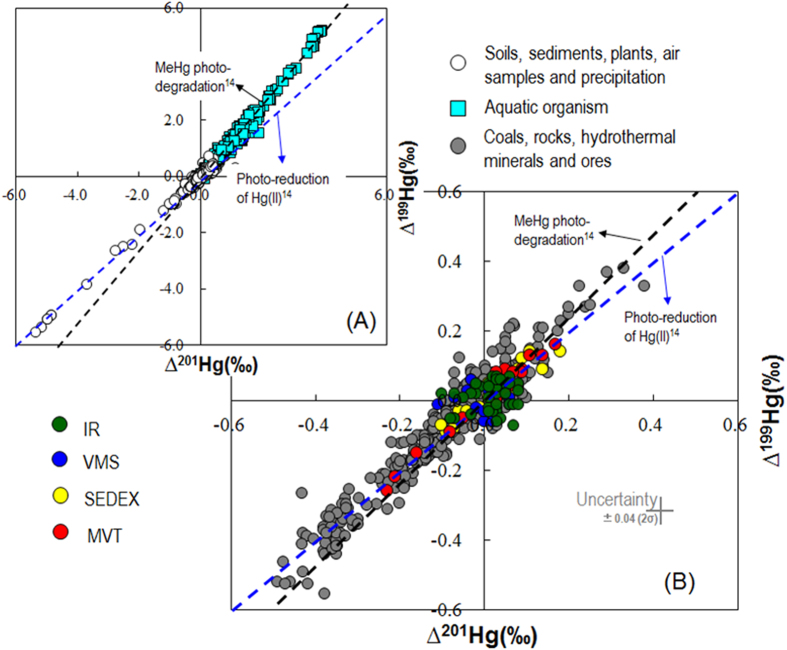
Plot of ∆^199^Hg versus ∆^201^Hg for different environmental samples (A based on previously published data summarized in Table S1) and sphalerites (B this study). The blue dashed line representing aqueous Hg(II) photoreduction^14^, has a slope of ~1.00. The black dashed line representing aqueous MeHg photodegradation^14^, has a slope of 1.36.

A dramatic variation in Hg-MIF was observed among different formations of Zn deposits. Hydrothermal fluids exposed to sunlight have been shown to generate Hg-MIF^18^. However, incorporation of Hg leached from sedimentary rocks with Hg-MIF may be more likely in sphalerites^22^. In our study, MVT (∆^199^Hg: −0.24 ~ + 0.14‰) and SEDEX (∆^199^Hg: −0.09 ~ + 0.18‰) deposits show large range of ∆^199^Hg values (Fig. 1). MVT deposits are stratabound, epigenetic orebodies that occur in clusters in carbonate formations^28^,^31^. Sulphur and metals of MVT deposits are derived from low-temperature hydrothermal solutions formed by diagenetic recrystallization of the carbonates^28^,^31^. SEDEX is interpreted to have been formed by release of ore-bearing fluids into ocean water, where heavy, hot brines mixed with cooler sea water, result in the precipitation of stratiform ore^29^,^31^. The ore-bearing hydrothermal fluids for SEDEX deposits are deep formational brines formed during sediments diagenesis^26^,^29^,^31^. During sediment diagenesis at relative high temperatures, the metals (including Hg) liberated as pore fluid are assumed to have a considerable sulphur and metal (e.g. Hg) 26content. Both SEDEX and MVT have no obvious spatial association with igneous rocks^29^,^31^. Leaching of sedimentary rocks by hydrothermal fluids then, are important sources of metals for both SEDEX and MVT deposits. As shown in Fig. 2, previous studies reported large Hg-MIF mainly in the surface of the crust, such as soil^39^,^40^,^41^, sediments^42^,^43^,^44^, water^45^,^46^,^47^, atmosphere^48^,^49^ and biological samples^14^,^50^,^51^,^52^. Sedimentation^42^,^43^,^44^, coalification^37^,^38^ and hydrothermal leaching of Hg from source-rocks^20^ have been shown unlikely to alter the MIF signature of Hg; the Hg-MIF signature has been observed in coals (∆^199^Hg: −0.66 to +0.38‰)^37^,^38^,^40^,^53^, peat bogs (∆^199^Hg: −0.50 to +0.22‰)^54^,^55^, and black shales^56^. Although no ∆^199^Hg data were reported, ∆^201^Hg values in sedimentary rocks (−0.10 to +0.28‰) have shown larger Hg-MIF compared to the metamorphic rocks (−0.06 to +0.03‰) and volcanic rocks (−0.09 to +0.05‰) in California Coast Ranges, USA^4^. In our study, two sphalerites (M-24 and M-25) with the largest Hg-MIF were collected from Lanuoma and Zaxikang in Tibet, both of which are MVT deposits and are found in carbonate-bearing rocks^57^,^58^. Cinnabars (Δ^199^Hg: −0.15 to +0.27‰) collected from South American Andes^24^ and a sphalerite sample (Δ^199^Hg = −0.12 ± 0.02‰, 2σ) collected from a SEDEX Zn deposit (Broken Hill Zn deposit, Australia)^22^ also show Hg-MIF signatures, which all indicate isotopic inheritance from interactions with sedimentary source-rocks. It is plausible then, that hydrothermal fluids have mobilized sedimentary Hg-MIF signatures and subsequently transferred them into deposited SEDEX and MVT ore bodies.

Samples from VMS (∆^199^Hg: −0.06 ~ + 0.06‰) and IR (∆^199^Hg: −0.07 ~ + 0.07‰) deposits show insignificant Hg-MIF (Fig. 1), which indicates that syngenetic Hg is probably the major Hg source. Similar insignificant Hg-MIF (mean ∆^199^Hg: −0.02 ± 0.02‰; range: 0 to +0.04‰; σ, n = 3) has been reported for syngenetic Hg in vent chimney samples from the Guaymas Basin sea-floor rift (USA)^18^. VMS deposits are deep-seated intrusions of magmatic materials in submarine divergent margins (e.g. mid-ocean ridges and back arc rifts)^26^,^30^,^31^. Metals in VMS deposits are mainly incompatible elements which are concentrated in the fluid phase of a volcanic eruption^26^,^30^,^31^ and transport of metals to VMS occurs via convection of hydrothermal fluids^30^,^31^. The heat supplied by the magma chamber (which sits below the volcanic edifice) can enrich the hydrothermal fluid in sulfur and metal ions^26^,^30^,^31^. Submarine volcanism and coeval chemical sedimentation may have provided a favorable setting for Hg transport and deposition. Mercury is found in abundance in VMS deposits associated with subaerial and submarine volcanism^22^. High levels of Hg concentration have been found in eclogite and peridotite in inclusions in kimberlite pipes^59^, which is thought to have a close relation with the formation of VMS deposits^22^. The IR deposits (such as skarn, manto, vein, etc) typically found in carbonate rocks in conjunction with magmatic systems, are characterized by mineral association of calcium and magnesium^22^,^31^,^34^. Similar to VMS, IR deposits have a close connection with igneous intrusions, and the ore-forming fluids are derived mainly from the igneous intrusions^22^,^31^,^34^. Ore bodies are commonly irregular in shape and may terminate abruptly at structural discontinuities^31^,^34^. Considering the close relation to deep-seated intrusions (e.g. volcanic and magmatic)^22^,^31^,^34^, mantle-derived Hg is believed to be most important source of Hg in VMS and IR deposits.

## Implications to the geochemical cycling of Hg

**Figure 3:**
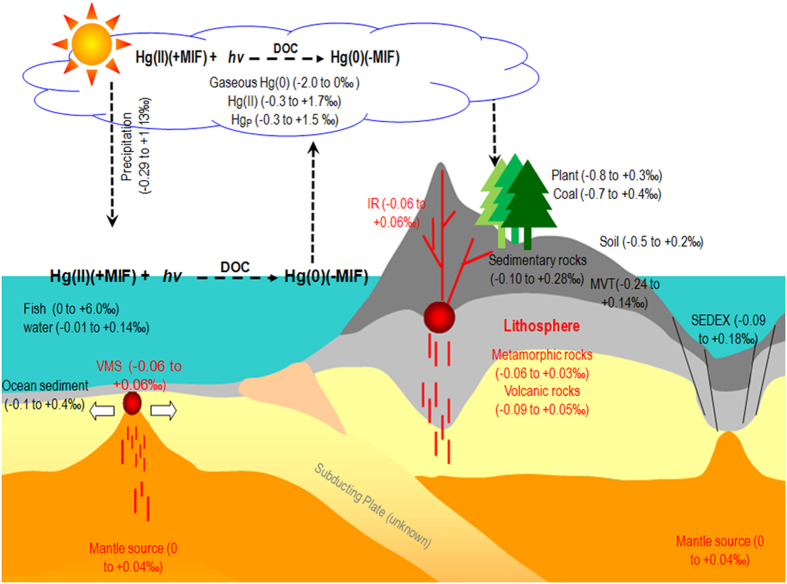
A conceptual model of global cycling of Hg MIF (Data source: Supplementary Table S1). This image is drawn by R. Yin.

A conceptual model for the geochemical cycling of Hg-MIF in different geochemical Hg pools is shown in Fig. 3. Photochemical reactions in the aquatic systems (e.g. ocean, water drops in cloud) play the foremost role in the generation of Hg-MIF^6^,^45^. Photoreduction of Hg(II) and MeHg impart negative Hg-MIF (Δ^199^Hg < 0) in the produced Hg(0), and therefore cause positive Hg-MIF (Δ^199^Hg > 0) in residual Hg(II) in the water phase^14^. The ocean is one of the largest Hg(0) sources to the atmosphere^1^ and gaseous Hg (Hg^0^_g_) represents the majority of atmospheric Hg pool^60^,^61^. It has a long atmospheric residence time of 0.5 to 2 years, allowing for hemispheric-to-global mixing and for transport of this metal far beyond the regions where it was emitted^1^. The biogeochemical cycling of Hg in the Earth’s surface may be capable of distributing the Hg-MIF in a global scale. Tectonic movements allow for the recycling of the Earth’s crustal materials, which transport Earth’s surface materials to the interior crust^19^. The Hg-MIF signals observed in different formations of Zn deposits, as well as other geological Hg pools (e.g., coals, rocks, and mineral deposits), have been interpreted as reflecting the insertion of Hg-MIF generated from the Earth’s surface to the interior crust. The magnitude of Hg-MIF in different geochemical reservoirs may be explained by the mixing of epigenetic and syngenetic Hg. Recycling of the Earth’s crustal material has continued for billions of years, therefore, the magnitudes of Hg-MIF in Hg pools of the interior crust may allow for temporal lags between Hg-MIF generation on the Earth’s surface and ultimate dilution by the syngenetic Hg. Our understanding of many key issues related to the geological cycling of Hg (e.g. the residence time and depth of the subducted Hg in the interior of the crust), may be enhanced by Hg-MIF signatures in future studies. Also, Hg-MIF may be useful in economic geology, particularly in the field of determination of metal sources in sulphide mineral deposits.

## Isotopic signature of Hg in sphalerites and its environmental implications

Based on the reserve of Zn (RZn) in each deposit, and the THg and Hg isotopic composition of its sphalerite (Supplementary Tables S2 and S3), the average isotopic compositions of Hg (*δ*^*202*^*Hg*_*average*_ and Δ^*199*^*Hg*_*average*_) in the 102 Zn deposits may be described by:


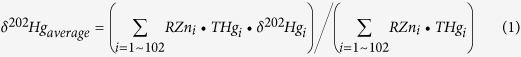



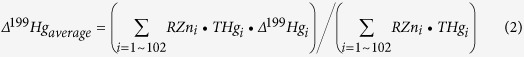


where *i* represents the number of each deposit; RZn_*i*_ represents the RZn in the deposit *i*; THg_*i*,_, *δ*^*202*^*Hg*_*i*_ and Δ^*199*^*Hg*_*i*_ represent the THg, δ^202^Hg and Δ^199^Hg values in sphalerite of deposit *i*, respectively. Our mass balance estimation is based on the assumption that the concentration and isotopic composition of Hg measured in a relatively small number of sphalerites for each deposit is representative of the entire deposit. Given the intra-deposit variations of Hg and the limited sample size in this study, our estimated results (*δ*^*202*^*Hg*_*average*_ = −0.58‰ and Δ^*199*^*Hg*_*average*_ = +0.03‰) may have relatively large uncertainties.

**Figure 4:**
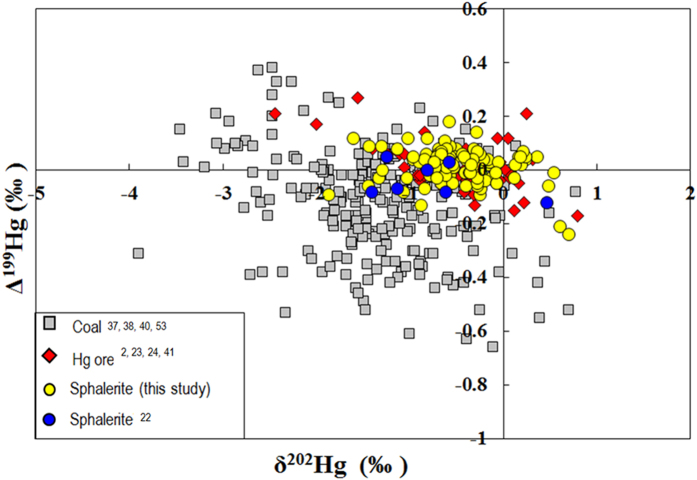
Δ^199^Hg versus δ^202^Hg in sphalerites, Hg ores and coal deposits.

Previous studies have revealed that coal combustion, Hg and Zn mining are major anthropogenic emission sources to the atmosphere^26^,^32^,^33^,^62^. In a plot of ∆^199^Hg vs. δ^202^Hg for sphalerites, Hg ores and coals (Fig. 4), most sphalerites overlap with Hg ores. ANOVA tests for δ^202^Hg (*P* = 0.87) and Δ^199^Hg (*P* = 0.57) show insignificant difference between sphalerites and Hg ores. However, most coal samples are outside the ranges of δ^202^Hg and Δ^199^Hg values for Zn and Hg ore deposits. ANOVA tests between coals and Zn/Hg ores showed significant difference in Δ^199^Hg (*P* = 0.03), but insignificant differences in δ^202^Hg (*P* = 0.80). This study implies that Hg isotopes may be useful to discriminate Hg and Zn mining from coal combustion on local, regional and global scales. Using Hg isotope to trace Hg emissions from Zn smelting requires a better understanding of how smelting processes may induce Hg isotope fractionation. Hg isotope fractionation has been observed during coal combustion^63^ and ore roasting^22^,^23^,^36^,^64^, resulting in isotope signatures different from the parent materials. Zn smelting requires roasting of sphalerites for desulfurization, which produces waste slag and flue gas containing gaseous Hg(0)^32^,^33^. Roasting of sphalerites inevitably leads to Hg(0) volatilization^32^,^33^, and elemental Hg(0) volatilization has shown to cause relative negative δ^202^Hg in the produced Hg(0)^10^,^11^, which may lead to relative positive δ^202^Hg in Zn slags. Sonke *et al.*^22^. demonstrated MDF of +0.4‰ in δ^202^Hg between Zn slags (δ^202^Hg: −0.24 ± 0.71‰, 2ó, n = 4) and sphalerite (δ^202^Hg: −0.65 ± 1.33‰, 2σ, n = 4) during Zn smelting. This study does not attempt to investigate Hg isotope fractionation that is likely to occur during zinc smelting and atmospheric transport. To reveal the true Hg isotopic signature of Chinese Zn smelting, more research on Hg isotope fractionation during hydrometallurgical processing is needed.

## Methods

### Sample information

Details of sample location, collection, preparation and Hg concentration (THg) analysis of 100 samples have been described by Yin *et al.*^26^. Two additional samples collected from the Lanuoma deposit (M-24) and Zaxikang deposit (M-24) in eastern Tibet were prepared similarly^22^. Relevant information (e.g., name and type) of all the deposits are summarized in Supplementary Table S3.

### Total mercury concentration and mercury isotopic composition analysis

Approximately 0.2 g of each sample was digested (95 °C, 1 hour) using a 5 mL aqua regia (HCl:HNO_3_ = 3:1, v-v). Certified reference material (NIST SRM 2711, Montana soil II) was digested in the same way. Sample digests of M-24, M-25 and NIST SRM 2711 were measured for THg using a previous method^22^. The THg recoveries of NIST SRM 2711 were in the range of 94 to 107‰ (n = 11). Based on the measured THg (Appendix Table A1), all sample digests were diluted to ~2 ng mL^−1^ with acid concentration <20%. Hg isotope ratios were measured using a Nu-Plasma MC-ICP-MS at the Institute of Geochemistry (Chinese Academy of Sciences) and a Neptune-Plus MC-ICP-MS at the Wisconsin State Laboratory of Hygiene (University of Wisconsin-Madison), following the methods described by Yin *et al.*^65^, and Foucher and Hintelmann^3^. An internal Tl standard (NIST SRM 997) was used for instrument mass bias correction. To reduce the matrix dependent mass bias, the concentrations of Hg and acid in the bracketing standard (NIST SRM 3133) and sample solutions were matched within 10%. Hg-MDF is expressed in δ^202^Hg notation in units of permil (‰) referenced to the NIST SRM 3133 Hg standard (analyzed before and after each sample)^2^:


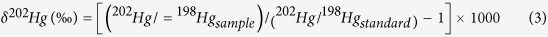


Hg-MIF is reported in Δ notation (Δ^xxx^Hg, deviation from mass dependency in units of permil, %) and is the difference between the measured Δ^xxx^Hg and the theoretically predicted Δ^xxx^Hg value using the following formula^2^:


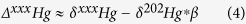


where β is equal to 0.2520 for ^199^Hg, 0.5024 for ^200^Hg, and 0.7520 for ^201^Hg, respectively^2^.

To assess the reproducibility of the Hg isotopic data, duplicate sample digests (n = 2) were measured. We also measured the UM-Almadén standard solution (ref. 2) once every 10 samples. Concentrations of Hg and acid were matched to the closely measured NIST-3133 solution. Data uncertainties of each sample adopt the larger values of either the external precision of the replication of the UM-Almadén solutions or the measurement uncertainty of duplicate sample digests. The overall average and uncertainty (σ, standard deviation) of UM-Almadén (δ^202^Hg: −0.50 ± 0.09‰; Δ^199^Hg: −0.03 ± 0.04‰; Δ^201^Hg: −0.02 ± 0.04‰; 2σ, n = 21) agreed with Blum and Bergquist^2^. Measurements of NIST SRM 2711 (δ^202^Hg: −0.21 ± 0.09‰; Δ^199^Hg: −0.17 ± 0.04‰; Δ^201^Hg: −0.19 ± 0.04‰, 2σ, n = 11) also agreed well with previous studies^38^,^40^.

## Additional Information

**How to cite this article**: Yin, R. *et al.* Mercury Isotopes as Proxies to Identify Sources and Environmental Impacts of Mercury in Sphalerites. *Sci. Rep.* **6**, 18686; doi: 10.1038/srep18686 (2016).

## Supplementary Material

Supplementary Information
