# Supplementary material for: Mercury Isotopes as Proxies to Identify Sources and Environmental Impacts of Mercury in Sphalerites
Source: Sci Rep. 2016 Jan 5;6:18686. doi: 10.1038/srep18686 (PMC4700498; doi:10.1038/srep18686)
Supplement: Supplementary Information [file srep18686-s1.doc]

**Supplementary**

**Mercury Isotopes as** [**Proxies**](http://www.baidu.com/link?url=QE3rJFerev-6lNYvhQVmlmrjwxpSR8Z0YJe6iU9I6vlX7WVhouL_Vt_YlZhEMjT899OLbHhpL-i4sX2B5jR7P9Hqcfn3ojptdDT-lsYuHlu) **to Identify the Sources and Environmental Impacts of Mercury in Sphalerites**

Runsheng Yin^1, 2^, **^3^**, Xinbin Feng^1^,^*^, James P. Hurley^2^, David P. Krabbenhoft^4^, Ryan F. Lepak^2^, Ruizhong Hu**^3^**, Qian Zhang**^3^**, Zhonggen Li^1^, Xianwu Bi**^3^**

*^1^State Key Laboratory of Environmental Geochemistry, Institute of Geochemistry, Chinese Academy of Sciences, Guiyang 550002, China*

*^2^Department of Civil and Environmental Engineering, Environmental Chemistry and Technology Program, University of Wisconsin-Madison, Madison, WI, 53706, USA*

***^3^****State Key Laboratory of Ore Deposit Geochemistry, Institute of Geochemistry, Chinese Academy of Sciences, Guiyang 550002, China*

*^4^U.S. Geological Survey, 8505 Research Way, Middleton, WI, 53562, USA*

*^*^Email: fengxinbin@vip.skleg.cn*

**Table S1** Previous published data on the Hg isotopic compositions in different environmental compartments

| Sample Type | Sample Location | Sample ID | δ^202^Hg | σ | Δ^199^Hg | σ | Δ^201^Hg | σ | *Data source* |
| --- | --- | --- | --- | --- | --- | --- | --- | --- | --- |
|  |  |  | (‰) | (‰) | (‰) | (‰) | (‰) | (‰) |  |
| Precipitation | California | Moss Beach | 0.02 |  | 0.16 |  | 0.19 |  | *Donovan et al., Chem. Geo., 2013* |
| Precipitation | California | Moss Beach (rep) | -0.01 |  | 0.33 |  | 0.31 |  | *Donovan et al., Chem. Geo., 2013* |
| Precipitation | California | San Jose | -0.01 |  | 0.35 |  | 0.19 |  | *Donovan et al., Chem. Geo., 2013* |
| Precipitation | California | Oakland | 0.19 |  | 0.29 |  | 0.27 |  | *Donovan et al., Chem. Geo., 2013* |
| Precipitation | Dexter | DXT-1 | -0.39 |  | 0.50 |  | 0.30 |  | *Gratz et al., 2010* |
| Precipitation | Dexter | DXT-2 | -0.34 |  | 0.45 |  | 0.47 |  | *Gratz et al., 2010* |
| Precipitation | Dexter | DXT-3A | -0.04 |  | 0.48 |  | 0.05 |  | *Gratz et al., 2010* |
| Precipitation | Dexter | DXT-5 | -0.29 |  | 0.22 |  | 0.17 |  | *Gratz et al., 2010* |
| Precipitation | Dexter | DXT-7 | -0.53 |  | 0.11 |  | 0.31 |  | *Gratz et al., 2010* |
| Precipitation | Dexter | DXT-11 | -0.49 |  | 0.14 |  | 0.37 |  | *Gratz et al., 2010* |
| Precipitation | Dexter | DXT-13 | -0.21 |  | 0.23 |  | 0.27 |  | *Gratz et al., 2010* |
| Precipitation | Dexter | DXT-26 | -0.41 |  | 0.07 |  | 0.09 |  | *Gratz et al., 2010* |
| Precipitation | Dexter | DXT-27 | 0.19 |  | 0.28 |  | 0.18 |  | *Gratz et al., 2010* |
| Precipitation | Dexter | DXT-28 | -0.31 |  | 0.25 |  | 0.18 |  | *Gratz et al., 2010* |
| Precipitation | Dexter | DXT-29 | -0.52 |  | 0.24 |  | 0.28 |  | *Gratz et al., 2010* |
| Precipitation | Holland | HOL-2 | -0.52 |  | 0.61 |  | 0.55 |  | *Gratz et al., 2010* |
| Precipitation | Holland | HOL-5 | 0.00 |  | 0.37 |  | 0.47 |  | *Gratz et al., 2010* |
| Precipitation | Holland | HOL-7 | -0.50 |  | 0.10 |  | 0.20 |  | *Gratz et al., 2010* |
| Precipitation | Chicago | UOC-1 | -0.27 |  | 0.43 |  | 0.43 |  | *Gratz et al., 2010* |
| Precipitation | Chicago | UOC-2A | -0.38 |  | 0.36 |  | 0.35 |  | *Gratz et al., 2010* |
| Precipitation | Chicago | UOC-2B | -0.40 |  | 0.36 |  | 0.33 |  | *Gratz et al., 2010* |
| Precipitation | Chicago | UOC-5 | -0.79 |  | 0.20 |  | 0.42 |  | *Gratz et al., 2010* |
| Precipitation | Chicago | UOC-7 | -0.40 |  | 0.14 |  | 0.23 |  | *Gratz et al., 2010* |
| Precipitation | Chicago | UOC-8 | 0.21 |  | 0.37 |  | 0.17 |  | *Gratz et al., 2010* |
| Precipitation | USA | CR-N | -3.87 |  | 0.34 |  | 0.36 |  | *Gehrke et al., 2012 EST* |
| Precipitation | USA | CR-NE | -1.12 |  | 0.62 |  | 0.54 |  | *Gehrke et al., 2012 EST* |
| Precipitation | USA | CR-S | -3.12 |  | 0.22 |  | 0.11 |  | *Gehrke et al., 2012 EST* |
| Precipitation | USA | CR-E | -3.15 |  | 0.45 |  | 0.21 |  | *Gehrke et al., 2012 EST* |
| Precipitation | USA | CR-N | -1.52 |  | 0.49 |  | 0.42 |  | *Gehrke et al., 2012 EST* |
| Precipitation | USA | CR-NE | -3.05 |  | 0.43 |  | 0.37 |  | *Gehrke et al., 2012 EST* |
| Precipitation | USA | CR-S | -3.40 |  | 0.37 |  | 0.17 |  | *Gehrke et al., 2012 EST* |
| Precipitation | USA | CR-E | -2.71 |  | 0.45 |  | 0.30 |  | *Gehrke et al., 2012 EST* |
| Precipitation | USA | CR-N | -1.58 |  | 0.31 |  | 0.27 |  | *Gehrke et al., 2012 EST* |
| Precipitation | USA | CR-S | -3.96 |  | 0.29 |  | 0.17 |  | *Gehrke et al., 2012 EST* |
| Precipitation | USA | CR-E | -2.90 |  | 0.31 |  | 0.23 |  | *Gehrke et al., 2012 EST* |
| Precipitation | USA | CR-S | -1.74 |  | 0.31 |  | 0.27 |  | *Gehrke et al., 2012 EST* |
| Precipitation | USA | CR-N | -3.76 |  | 0.32 |  | 0.19 |  | *Gehrke et al., 2012 EST* |
| Precipitation | USA | CR-NE | -4.37 |  | 0.41 |  | 0.24 |  | *Gehrke et al., 2012 EST* |
| Precipitation | USA | CR-S | -3.80 |  | 0.36 |  | 0.29 |  | *Gehrke et al., 2012 EST* |
| Precipitation | USA | CR-E | -4.00 |  | 0.33 |  | 0.20 |  | *Gehrke et al., 2012 EST* |
| Precipitation | USA | CR-NE | -1.66 |  | 0.22 |  | 0.14 |  | *Gehrke et al., 2012 EST* |
| Precipitation | USA | CR-E | -2.48 |  | 0.09 |  | 0.00 |  | *Gehrke et al., 2012 EST* |
| Precipitation | USA | CR-NE | -2.47 |  | 0.15 |  | 0.19 |  | *Gehrke et al., 2012 EST* |
| Precipitation | USA | CR-S | -2.11 |  | 0.31 |  | 0.29 |  | *Gehrke et al., 2012 EST* |
| Precipitation | USA | CR-E | -1.41 |  | 0.22 |  | 0.21 |  | *Gehrke et al., 2012 EST* |
| Precipitation | USA | CR-N | -2.42 |  | 0.16 |  | 0.12 |  | *Gehrke et al., 2012 EST* |
| Precipitation | USA | CR-E | -2.58 |  | 0.23 |  | 0.09 |  | *Gehrke et al., 2012 EST* |
| Precipitation | USA | CR-NE | -1.85 |  | 0.23 |  | 0.18 |  | *Gehrke et al., 2012 EST* |
| Precipitation | USA | CR-S | -3.77 |  | 0.32 |  | 0.21 |  | *Gehrke et al., 2012 EST* |
| Precipitation | USA | CR-E | -1.91 |  | 0.24 |  | 0.15 |  | *Gehrke et al., 2012 EST* |
| Precipitation | USA | CR-E-REP | -2.09 |  | 0.28 |  | 0.26 |  | *Gehrke et al., 2012 EST* |
| Precipitation | USA | CR-NE | -0.26 |  | 0.12 |  | 0.25 |  | *Gehrke et al., 2012 EST* |
| Precipitation | USA | CR-NE | -0.53 |  | 0.50 |  | 0.50 |  | *Gehrke et al., 2012 EST* |
| Precipitation | USA | SDK | 0.13 |  | 0.35 |  | 0.30 |  | *Gehrke et al., 2012 EST* |
| Precipitation | USA | TPA | 0.10 |  | 0.25 |  | 0.24 |  | *Gehrke et al., 2012 EST* |
| Precipitation | USA | HSB | 0.10 |  | -0.03 |  | 0.12 |  | *Gehrke et al., 2012 EST* |
| Precipitation | USA | UCF | -0.43 |  | 0.54 |  | 0.56 |  | *Gehrke et al., 2012 EST* |
| Precipitation | USA | MKA | -0.04 |  | -0.18 |  | -0.11 |  | *Gehrke et al., 2012 EST* |
| Precipitation | USA | KYB | 0.21 |  | 0.43 |  | 0.50 |  | *Gehrke et al., 2012 EST* |
| Precipitation | USA | MIA | 0.12 |  | -0.12 |  | -0.01 |  | *Gehrke et al., 2012 EST* |
| Precipitation | USA | MIA-REP | 0.13 |  | -0.06 |  | 0.01 |  | *Gehrke et al., 2012 EST* |
| Precipitation | USA | DVE | 0.13 |  | 0.29 |  | 0.15 |  | *Gehrke et al., 2012 EST* |
| Precipitation | USA | ENP | 0.08 |  | 0.40 |  | 0.44 |  | *Gehrke et al., 2012 EST* |
| Precipitation | USA | ENP | 0.11 |  | -0.08 |  | 0.06 |  | *Gehrke et al., 2012 EST* |
| Precipitation | USA | ENP | 0.07 |  | -0.02 |  | 0.01 |  | *Gehrke et al., 2012 EST* |
| Precipitation | USA | ENP | 0.10 |  | 0.30 |  | 0.47 |  | *Gehrke et al., 2012 EST* |
| Precipitation | USA | ENP-REP | -0.09 |  | 0.17 |  | 0.32 |  | *Gehrke et al., 2012 EST* |
| Precipitation | USA | ENP | 0.14 |  | -0.11 |  | -0.03 |  | *Gehrke et al., 2012 EST* |
| Precipitation | USA | ENP-REP | 0.03 |  | -0.12 |  | -0.04 |  | *Gehrke et al., 2012 EST* |
| Precipitation |  |  | -0.74 |  | 0.50 |  | 0.45 |  | *Demers et al., 2013* |
| Precipitation |  |  | -0.39 |  | 0.82 |  | 0.61 |  | *Demers et al., 2013* |
| Precipitation |  |  | 0.06 |  | 0.64 |  | 0.40 |  | *Demers et al., 2013* |
| Precipitation |  |  | -0.41 |  | 0.28 |  | 0.50 |  | *Demers et al., 2013* |
| Precipitation |  |  | -0.23 |  | 0.16 |  | 0.26 |  | *Demers et al., 2013* |
| Precipitation |  |  | -1.35 |  | 0.32 |  | 1.11 |  | *Chen et al., 2012* |
| Precipitation |  |  | -0.02 |  | -0.29 |  | -0.24 |  | *Chen et al., 2012* |
| Precipitation |  |  | -1.36 |  | 0.36 |  | 0.35 |  | *Chen et al., 2012* |
| Precipitation |  |  | -0.42 |  | 0.37 |  | 0.32 |  | *Chen et al., 2012* |
| Precipitation |  |  | -0.82 |  | 0.97 |  | 0.94 |  | *Chen et al., 2012* |
| Precipitation |  |  | -0.72 |  | -0.01 |  | -0.29 |  | *Chen et al., 2012* |
| Precipitation |  |  | -0.68 |  | 0.59 |  | 0.54 |  | *Chen et al., 2012* |
| Precipitation |  |  | -1.19 |  | 0.32 |  | 0.35 |  | *Chen et al., 2012* |
| Precipitation |  |  | -0.68 |  | 0.54 |  | 0.47 |  | *Chen et al., 2012* |
| Precipitation |  |  | -0.55 |  | 0.47 |  | 0.48 |  | *Chen et al., 2012* |
| Precipitation |  |  | -1.25 |  | 0.49 |  | 0.61 |  | *Chen et al., 2012* |
| Precipitation |  |  | -1.02 |  | 0.73 |  | 0.74 |  | *Chen et al., 2012* |
| Precipitation |  |  | -1.27 |  | 0.72 |  | 0.65 |  | *Chen et al., 2012* |
| Precipitation |  |  | -0.80 |  | 0.56 |  | 0.51 |  | *Chen et al., 2012* |
| Precipitation |  |  | -1.04 |  | 1.13 |  | 1.00 |  | *Chen et al., 2012* |
| Precipitation |  |  | -1.18 |  | 0.43 |  | 0.25 |  | *Chen et al., 2012* |
| Precipitation |  |  | -1.59 |  | 0.75 |  | 0.78 |  | *Chen et al., 2012* |
| Precipitation |  |  | -1.37 |  | 0.75 |  | 0.71 |  | *Chen et al., 2012* |
| Precipitation |  |  | -1.03 |  | 0.72 |  | 0.61 |  | *Chen et al., 2012* |
| Precipitation |  |  | -1.48 |  | 0.70 |  | 0.71 |  | *Chen et al., 2012* |
| Precipitation |  |  | -1.20 |  | 0.50 |  | 0.38 |  | *Chen et al., 2012* |
| Precipitation |  |  | -1.41 |  | 0.47 |  | 0.57 |  | *Chen et al., 2012* |
| Precipitation |  |  | -1.45 |  | 0.44 |  | 0.43 |  | *Chen et al., 2012* |
| Precipitation |  |  | -1.10 |  | 0.73 |  | 0.65 |  | *Chen et al., 2012* |
| Precipitation |  |  | -1.86 |  | -0.02 |  | -0.04 |  | *Sherman et al., 2012* |
| Precipitation |  |  | -3.87 |  | 0.34 |  | 0.36 |  | *Sherman et al., 2012* |
| Precipitation |  |  | -1.12 |  | 0.62 |  | 0.54 |  | *Sherman et al., 2012* |
| Precipitation |  |  | -3.12 |  | 0.22 |  | 0.11 |  | *Sherman et al., 2012* |
| Precipitation |  |  | -3.15 |  | 0.45 |  | 0.21 |  | *Sherman et al., 2012* |
| Precipitation |  |  | -1.52 |  | 0.49 |  | 0.42 |  | *Sherman et al., 2012* |
| Precipitation |  |  | -3.05 |  | 0.43 |  | 0.37 |  | *Sherman et al., 2012* |
| Precipitation |  |  | -3.40 |  | 0.37 |  | 0.17 |  | *Sherman et al., 2012* |
| Precipitation |  |  | -2.71 |  | 0.45 |  | 0.30 |  | *Sherman et al., 2012* |
| Precipitation |  |  | -1.58 |  | 0.31 |  | 0.27 |  | *Sherman et al., 2012* |
| Precipitation |  |  | -3.96 |  | 0.29 |  | 0.17 |  | *Sherman et al., 2012* |
| Precipitation |  |  | -2.90 |  | 0.31 |  | 0.23 |  | *Sherman et al., 2012* |
| Precipitation |  |  | -1.74 |  | 0.31 |  | 0.27 |  | *Sherman et al., 2012* |
| Precipitation |  |  | -3.76 |  | 0.32 |  | 0.19 |  | *Sherman et al., 2012* |
| Precipitation |  |  | -4.37 |  | 0.41 |  | 0.24 |  | *Sherman et al., 2012* |
| Precipitation |  |  | -3.80 |  | 0.36 |  | 0.29 |  | *Sherman et al., 2012* |
| Precipitation |  |  | -4.00 |  | 0.33 |  | 0.20 |  | *Sherman et al., 2012* |
| Precipitation |  |  | -1.66 |  | 0.22 |  | 0.14 |  | *Sherman et al., 2012* |
| Precipitation |  |  | -2.48 |  | 0.09 |  | 0.00 |  | *Sherman et al., 2012* |
| Precipitation |  |  | -2.47 |  | 0.15 |  | 0.19 |  | *Sherman et al., 2012* |
| Precipitation |  |  | -2.11 |  | 0.31 |  | 0.29 |  | *Sherman et al., 2012* |
| Precipitation |  |  | -1.41 |  | 0.22 |  | 0.21 |  | *Sherman et al., 2012* |
| Precipitation |  |  | -2.42 |  | 0.16 |  | 0.12 |  | *Sherman et al., 2012* |
| Precipitation |  |  | -2.58 |  | 0.23 |  | 0.09 |  | *Sherman et al., 2012* |
| Precipitation |  |  | -1.85 |  | 0.23 |  | 0.18 |  | *Sherman et al., 2012* |
| Precipitation |  |  | -3.77 |  | 0.32 |  | 0.21 |  | *Sherman et al., 2012* |
| Precipitation |  |  | -1.91 |  | 0.24 |  | 0.15 |  | *Sherman et al., 2012* |
| Precipitation |  |  | -2.09 |  | 0.28 |  | 0.26 |  | *Sherman et al., 2012* |
| Precipitation |  |  | -0.26 |  | 0.12 |  | 0.25 |  | *Sherman et al., 2012* |
| Precipitation |  |  | -0.53 |  | 0.50 |  | 0.50 |  | *Sherman et al., 2012* |
| Precipitation |  |  | 0.13 |  | 0.35 |  | 0.30 |  | *Sherman et al., 2012* |
| Precipitation |  |  | 0.10 |  | 0.25 |  | 0.24 |  | *Sherman et al., 2012* |
| Precipitation |  |  | 0.10 |  | -0.03 |  | 0.12 |  | *Sherman et al., 2012* |
| Precipitation |  |  | -0.43 |  | 0.54 |  | 0.56 |  | *Sherman et al., 2012* |
| Precipitation |  |  | -0.04 |  | -0.18 |  | -0.11 |  | *Sherman et al., 2012* |
| Precipitation |  |  | 0.21 |  | 0.43 |  | 0.50 |  | *Sherman et al., 2012* |
| Precipitation |  |  | 0.12 |  | -0.12 |  | -0.01 |  | *Sherman et al., 2012* |
| Precipitation |  |  | 0.13 |  | -0.06 |  | 0.01 |  | *Sherman et al., 2012* |
| Precipitation |  |  | 0.13 |  | 0.29 |  | 0.15 |  | *Sherman et al., 2012* |
| Precipitation |  |  | 0.08 |  | 0.40 |  | 0.44 |  | *Sherman et al., 2012* |
| Precipitation |  |  | 0.11 |  | -0.08 |  | 0.06 |  | *Sherman et al., 2012* |
| Precipitation |  |  | 0.07 |  | -0.02 |  | 0.01 |  | *Sherman et al., 2012* |
| Precipitation |  |  | 0.10 |  | 0.30 |  | 0.47 |  | *Sherman et al., 2012* |
| Precipitation |  |  | -0.09 |  | 0.17 |  | 0.32 |  | *Sherman et al., 2012* |
| Precipitation |  |  | 0.14 |  | -0.11 |  | -0.03 |  | *Sherman et al., 2012* |
| Precipitation |  |  | 0.03 |  | -0.12 |  | -0.04 |  | *Sherman et al., 2012* |
| Precipitation |  |  | -0.39 |  | 0.50 |  | 0.30 |  | *Gratz et al., 2010* |
| Precipitation |  |  | -0.34 |  | 0.45 |  | 0.47 |  | *Gratz et al., 2010* |
| Precipitation |  |  | -0.04 |  | 0.48 |  | 0.05 |  | *Gratz et al., 2010* |
| Precipitation |  |  | -0.29 |  | 0.22 |  | 0.17 |  | *Gratz et al., 2010* |
| Precipitation |  |  | -0.53 |  | 0.11 |  | 0.31 |  | *Gratz et al., 2010* |
| Precipitation |  |  | -0.49 |  | 0.14 |  | 0.37 |  | *Gratz et al., 2010* |
| Precipitation |  |  | -0.21 |  | 0.23 |  | 0.27 |  | *Gratz et al., 2010* |
| Precipitation |  |  | -0.41 |  | 0.07 |  | 0.09 |  | *Gratz et al., 2010* |
| Precipitation |  |  | 0.19 |  | 0.28 |  | 0.18 |  | *Gratz et al., 2010* |
| Precipitation |  |  | -0.31 |  | 0.25 |  | 0.18 |  | *Gratz et al., 2010* |
| Precipitation |  |  | -0.52 |  | 0.24 |  | 0.28 |  | *Gratz et al., 2010* |
| Precipitation |  |  | -0.52 |  | 0.61 |  | 0.55 |  | *Gratz et al., 2010* |
| Precipitation |  |  | 0.00 |  | 0.37 |  | 0.47 |  | *Gratz et al., 2010* |
| Precipitation |  |  | -0.50 |  | 0.10 |  | 0.20 |  | *Gratz et al., 2010* |
| Precipitation |  |  | -0.27 |  | 0.43 |  | 0.43 |  | *Gratz et al., 2010* |
| Precipitation |  |  | -0.38 |  | 0.36 |  | 0.35 |  | *Gratz et al., 2010* |
| Precipitation |  |  | -0.40 |  | 0.36 |  | 0.33 |  | *Gratz et al., 2010* |
| Precipitation |  |  | -0.79 |  | 0.20 |  | 0.42 |  | *Gratz et al., 2010* |
| Precipitation |  |  | -0.40 |  | 0.14 |  | 0.23 |  | *Gratz et al., 2010* |
| Precipitation |  |  | 0.21 |  | 0.37 |  | 0.17 |  | *Gratz et al., 2010* |
|  |  |  |  |  |  |  |  |  |  |
| Fish | California | CB1MI | 0.05 |  | 1.07 |  | 0.87 |  | *Gehrke et al., ES&T, 2011* |
| Fish | California | CB5MI | 0.01 |  | 1.52 |  | 1.25 |  | *Gehrke et al., ES&T, 2011* |
| Fish | California | LSB1MI | 0.60 |  | 0.71 |  | 0.57 |  | *Gehrke et al., ES&T, 2011* |
| Fish | California | LSB2MI | 0.22 |  | 0.72 |  | 0.56 |  | *Gehrke et al., ES&T, 2011* |
| Fish | California | LSB3MI | 0.31 |  | 0.86 |  | 0.66 |  | *Gehrke et al., ES&T, 2011* |
| Fish | California | LSB4MI | 0.53 |  | 0.74 |  | 0.60 |  | *Gehrke et al., ES&T, 2011* |
| Fish | California | LSB5MI | 0.48 |  | 0.70 |  | 0.58 |  | *Gehrke et al., ES&T, 2011* |
| Fish | California | LSB6MI | 0.42 |  | 0.63 |  | 0.49 |  | *Gehrke et al., ES&T, 2011* |
| Fish | California | SoB1MI | 0.41 |  | 0.66 |  | 0.53 |  | *Gehrke et al., ES&T, 2011* |
| Fish | California | SoB2MI | 0.10 |  | 1.05 |  | 0.83 |  | *Gehrke et al., ES&T, 2011* |
| Fish | California | SPB1MI | -0.03 |  | 0.58 |  | 0.42 |  | *Gehrke et al., ES&T, 2011* |
| Fish | California | SPB2MI | -0.25 |  | 0.61 |  | 0.49 |  | *Gehrke et al., ES&T, 2011* |
| Fish | California | SPB3MI | -0.03 |  | 0.61 |  | 0.49 |  | *Gehrke et al., ES&T, 2011* |
| Fish | California | SPB4MI | -0.10 |  | 0.46 |  | 0.38 |  | *Gehrke et al., ES&T, 2011* |
| Fish | California | SPB5MI | -0.19 |  | 0.50 |  | 0.42 |  | *Gehrke et al., ES&T, 2011* |
| Fish | California | SuiB1MI | -0.08 |  | 0.51 |  | 0.40 |  | *Gehrke et al., ES&T, 2011* |
| Fish | California | SuiB2MI | -0.11 |  | 0.64 |  | 0.53 |  | *Gehrke et al., ES&T, 2011* |
| Fish | California | SuiB3MI | 0.21 |  | 0.86 |  | 0.69 |  | *Gehrke et al., ES&T, 2011* |
| Fish | California | SuiB5MI | 0.31 |  | 1.55 |  | 1.21 |  | *Gehrke et al., ES&T, 2011* |
| Fish | California | SuiB6MI | 0.13 |  | 1.08 |  | 0.82 |  | *Gehrke et al., ES&T, 2011* |
| Fish | California | SuiB4MI | 0.01 |  | 0.94 |  | 0.71 |  | *Gehrke et al., ES&T, 2011* |
| Fish | California | CB1TO | 0.11 |  | 1.05 |  | 0.82 |  | *Gehrke et al., ES&T, 2011* |
| Fish | California | CB2TO | -0.04 |  | 0.65 |  | 0.50 |  | *Gehrke et al., ES&T, 2011* |
| Fish | California | CB4TO | 0.18 |  | 1.02 |  | 0.82 |  | *Gehrke et al., ES&T, 2011* |
| Fish | California | CB5TO | 0.08 |  | 0.65 |  | 0.56 |  | *Gehrke et al., ES&T, 2011* |
| Fish | California | CB6TO | -0.03 |  | 0.67 |  | 0.56 |  | *Gehrke et al., ES&T, 2011* |
| Fish | California | LSB3TO | 0.33 |  | 0.70 |  | 0.57 |  | *Gehrke et al., ES&T, 2011* |
| Fish | California | LSB4TO | 0.57 |  | 0.66 |  | 0.52 |  | *Gehrke et al., ES&T, 2011* |
| Fish | California | LSB5TO | 0.47 |  | 0.60 |  | 0.50 |  | *Gehrke et al., ES&T, 2011* |
| Fish | California | LSB6TO | 0.34 |  | 0.63 |  | 0.45 |  | *Gehrke et al., ES&T, 2011* |
| Fish | California | SoB5TO | 0.03 |  | 0.86 |  | 0.69 |  | *Gehrke et al., ES&T, 2011* |
| Fish | California | SoB6TO | 0.16 |  | 0.57 |  | 0.48 |  | *Gehrke et al., ES&T, 2011* |
| Fish | California | SPB1TO | -0.06 |  | 0.67 |  | 0.55 |  | *Gehrke et al., ES&T, 2011* |
| Fish | California | SuiB2TO | -0.03 |  | 0.71 |  | 0.57 |  | *Gehrke et al., ES&T, 2011* |
| Fish | Gulf of Mexico | GS16 | -0.81 |  | 0.56 |  | 0.39 |  | *Senn et al., ES&T, 2010* |
| Fish | Gulf of Mexico | GS18 | -0.34 |  | 0.62 |  | 0.49 |  | *Senn et al., ES&T, 2010* |
| Fish | Gulf of Mexico | GS8 | -0.03 |  | 0.72 |  | 0.56 |  | *Senn et al., ES&T, 2010* |
| Fish | Gulf of Mexico | RD1 | -1.02 |  | 0.37 |  | 0.28 |  | *Senn et al., ES&T, 2010* |
| Fish | Gulf of Mexico | RD2 | -0.26 |  | 0.39 |  | 0.25 |  | *Senn et al., ES&T, 2010* |
| Fish | Gulf of Mexico | RD3 | -0.83 |  | 0.43 |  | 0.28 |  | *Senn et al., ES&T, 2010* |
| Fish | Gulf of Mexico | RS30 | -0.49 |  | 0.50 |  | 0.36 |  | *Senn et al., ES&T, 2010* |
| Fish | Gulf of Mexico | RS38 | -0.36 |  | 0.60 |  | 0.50 |  | *Senn et al., ES&T, 2010* |
| Fish | Gulf of Mexico | RS42 | -0.44 |  | 0.52 |  | 0.32 |  | *Senn et al., ES&T, 2010* |
| Fish | Gulf of Mexico | ST3 | -0.85 |  | 0.59 |  | 0.47 |  | *Senn et al., ES&T, 2010* |
| Fish | Gulf of Mexico | BR3 | 0.22 |  | 1.14 |  | 0.91 |  | *Senn et al., ES&T, 2010* |
| Fish | Gulf of Mexico | BR4 | 0.20 |  | 1.07 |  | 0.86 |  | *Senn et al., ES&T, 2010* |
| Fish | Gulf of Mexico | BR5 | 0.15 |  | 1.13 |  | 0.92 |  | *Senn et al., ES&T, 2010* |
| Fish | Gulf of Mexico | BR6 | 0.51 |  | 1.15 |  | 0.92 |  | *Senn et al., ES&T, 2010* |
| Fish | Gulf of Mexico | SM2 | -0.59 |  | 0.97 |  | 0.75 |  | *Senn et al., ES&T, 2010* |
| Fish | Gulf of Mexico | SM3 | -0.12 |  | 0.69 |  | 0.52 |  | *Senn et al., ES&T, 2010* |
| Fish | Gulf of Mexico | SM6 | 0.03 |  | 0.95 |  | 0.70 |  | *Senn et al., ES&T, 2010* |
| Fish | Gulf of Mexico | SM7 | 0.27 |  | 0.83 |  | 0.68 |  | *Senn et al., ES&T, 2010* |
| Fish | Gulf of Mexico | BT11 | 0.60 |  | 1.92 |  | 1.52 |  | *Senn et al., ES&T, 2010* |
| Fish | Gulf of Mexico | BT13 | 0.35 |  | 1.46 |  | 1.16 |  | *Senn et al., ES&T, 2010* |
| Fish | Gulf of Mexico | BT18 | 0.52 |  | 1.38 |  | 1.11 |  | *Senn et al., ES&T, 2010* |
| Fish | Gulf of Mexico | BT2 | 0.24 |  | 1.19 |  | 0.97 |  | *Senn et al., ES&T, 2010* |
| Fish | Gulf of Mexico | BT22 | 0.37 |  | 1.34 |  | 1.04 |  | *Senn et al., ES&T, 2010* |
| Fish | Gulf of Mexico | BT3 | 0.39 |  | 1.68 |  | 1.33 |  | *Senn et al., ES&T, 2010* |
| Fish | Gulf of Mexico | BT9 | 0.09 |  | 1.43 |  | 1.10 |  | *Senn et al., ES&T, 2010* |
| Fish | Gulf of Mexico | YT12 | 0.25 |  | 1.48 |  | 1.13 |  | *Senn et al., ES&T, 2010* |
| Fish | Gulf of Mexico | YT17 | 0.20 |  | 1.59 |  | 1.28 |  | *Senn et al., ES&T, 2010* |
| Fish | Gulf of Mexico | YT18 | 0.53 |  | 1.43 |  | 1.15 |  | *Senn et al., ES&T, 2010* |
| Fish | Gulf of Mexico | YT19 | 0.49 |  | 2.52 |  | 2.04 |  | *Senn et al., ES&T, 2010* |
| Fish | Gulf of Mexico | YT2 | 0.36 |  | 2.72 |  | 2.24 |  | *Senn et al., ES&T, 2010* |
| Fish | Gulf of Mexico | YT5 | 0.56 |  | 2.01 |  | 1.62 |  | *Senn et al., ES&T, 2010* |
| Fish | Gulf of Mexico | YT7 | 0.73 |  | 2.34 |  | 1.89 |  | *Senn et al., ES&T, 2010* |
| Fish | California | California roach fry | -1.00 |  | 1.00 |  | 0.74 |  | *Tsui et al., ES&T, 2012* |
| Fish | California | Steelhead fish fry | -0.86 |  | 1.31 |  | 0.95 |  | *Tsui et al., ES&T, 2012* |
| Fish | California | Crayfish | -1.23 |  | 0.70 |  | 0.57 |  | *Tsui et al., ES&T, 2012* |
| Fish | California | Armored caddisfly scraper | -0.98 |  | 1.31 |  | 1.04 |  | *Tsui et al., ES&T, 2012* |
| Fish | California | Cased caddisfly grazer | -0.94 |  | 1.02 |  | 0.84 |  | *Tsui et al., ES&T, 2012* |
| Fish | California | Flathead mayfly | -1.13 |  | 1.10 |  | 0.91 |  | *Tsui et al., ES&T, 2012* |
| Fish | California | Black fly larvae | -1.22 |  | 0.64 |  | 0.47 |  | *Tsui et al., ES&T, 2012* |
| Fish | California | Salmonfly | -1.30 |  | 0.83 |  | 0.52 |  | *Tsui et al., ES&T, 2012* |
| Fish | California | Mayfly larvae | -0.98 |  | 0.98 |  | 0.70 |  | *Tsui et al., ES&T, 2012* |
| Fish | California | Net-spilling caddisfly larvae | -1.19 |  | 1.22 |  | 1.02 |  | *Tsui et al., ES&T, 2012* |
| Fish | California | Perlidae stonefly larvae | -0.89 |  | 1.13 |  | 0.84 |  | *Tsui et al., ES&T, 2012* |
| Fish | California | Predaceous caddisflies | -0.94 |  | 0.79 |  | 0.61 |  | *Tsui et al., ES&T, 2012* |
| Fish | California | Megaloptera | -0.91 |  | 0.84 |  | 0.61 |  | *Tsui et al., ES&T, 2012* |
| Fish | California | Snails | -1.06 |  | 0.50 |  | 0.38 |  | *Tsui et al., ES&T, 2012* |
| Fish | California | Detritivores | -1.38 |  | 0.52 |  | 0.45 |  | *Tsui et al., ES&T, 2012* |
| Fish | California | Giant orange sedge larvae | -0.91 |  | 1.00 |  | 0.76 |  | *Tsui et al., ES&T, 2012* |
| Fish | California | Pearl-shell mussel | -1.11 |  | 0.04 |  | -0.02 |  | *Tsui et al., ES&T, 2012* |
| Fish | California | Perlidae stonefly larvae | -1.14 |  | 0.86 |  | 0.67 |  | *Tsui et al., ES&T, 2012* |
| Fish | California | Dragonfly larvae | -1.19 |  | 0.70 |  | 0.54 |  | *Tsui et al., ES&T, 2012* |
| Fish | California | Water strider | -0.90 |  | 1.12 |  | 0.86 |  | *Tsui et al., ES&T, 2012* |
| Fish | California | Collector | -1.04 |  | 1.97 |  | 1.53 |  | *Tsui et al., 2013* |
| Fish | California | Collector | -1.12 |  | 2.00 |  | 1.45 |  | *Tsui et al., 2013* |
| Fish | California | Collector | -1.13 |  | 1.10 |  | 0.91 |  | *Tsui et al., 2013* |
| Fish | California | Collector | -1.02 |  | 0.93 |  | 0.71 |  | *Tsui et al., 2013* |
| Fish | California | Collector | -1.22 |  | 0.46 |  | 0.40 |  | *Tsui et al., 2013* |
| Fish | California | Collector | -1.48 |  | 0.44 |  | 0.38 |  | *Tsui et al., 2013* |
| Fish | California | Collector | -1.10 |  | 0.82 |  | 0.57 |  | *Tsui et al., 2013* |
| Fish | California | Collector | -1.51 |  | 0.02 |  | -0.15 |  | *Tsui et al., 2013* |
| Fish | California | Scraper | -0.81 |  | 2.13 |  | 1.65 |  | *Tsui et al., 2013* |
| Fish | California | Scraper | -0.98 |  | 1.31 |  | 1.04 |  | *Tsui et al., 2013* |
| Fish | California | Scraper | -0.78 |  | 1.22 |  | 0.92 |  | *Tsui et al., 2013* |
| Fish | California | Scraper | -0.84 |  | 1.04 |  | 0.55 |  | *Tsui et al., 2013* |
| Fish | California | Scraper | -1.33 |  | 0.91 |  | 0.68 |  | *Tsui et al., 2013* |
| Fish | California | Scraper | -0.77 |  | 0.82 |  | 0.45 |  | *Tsui et al., 2013* |
| Fish | California | Filterer | -0.98 |  | 1.46 |  | 1.07 |  | *Tsui et al., 2013* |
| Fish | California | Filterer | -0.95 |  | 1.47 |  | 1.16 |  | *Tsui et al., 2013* |
| Fish | California | Filterer | -0.91 |  | 0.98 |  | 0.73 |  | *Tsui et al., 2013* |
| Fish | California | Filterer | -0.80 |  | 0.94 |  | 0.56 |  | *Tsui et al., 2013* |
| Fish | California | Filterer | -1.00 |  | 0.49 |  | 0.28 |  | *Tsui et al., 2013* |
| Fish | California | Filterer | -1.25 |  | 0.52 |  | 0.30 |  | *Tsui et al., 2013* |
| Fish | California | Filterer | -0.61 |  | 1.50 |  | 1.23 |  | *Tsui et al., 2013* |
| Fish | California | Filterer | -0.66 |  | 0.60 |  | 0.46 |  | *Tsui et al., 2013* |
| Fish | California | Predator | -0.92 |  | 1.79 |  | 1.23 |  | *Tsui et al., 2013* |
| Fish | California | Predator | -0.81 |  | 1.68 |  | 1.33 |  | *Tsui et al., 2013* |
| Fish | California | Predator | -0.89 |  | 1.13 |  | 0.84 |  | *Tsui et al., 2013* |
| Fish | California | Predator | -0.97 |  | 0.86 |  | 0.72 |  | *Tsui et al., 2013* |
| Fish | California | Predator | -1.01 |  | 0.63 |  | 0.45 |  | *Tsui et al., 2013* |
| Fish | California | Predator | -1.21 |  | 0.63 |  | 0.43 |  | *Tsui et al., 2013* |
| Fish | California | Predator | -0.65 |  | 1.08 |  | 0.90 |  | *Tsui et al., 2013* |
| Fish | California | Predator | -0.77 |  | 0.52 |  | 0.43 |  | *Tsui et al., 2013* |
| Fish | Lake Baikal | CRM 464 | 0.55 |  | 1.54 |  | 1.88 |  | *Perrot et al., 2011* |
| Fish | Canada | Lake D-1 | -0.68 |  | 1.53 |  | 1.39 |  | *Gantner et al., 2009 EST* |
| Fish | Canada | Lake D-2 | -0.66 |  | 1.43 |  | 1.25 |  | *Gantner et al., 2009 EST* |
| Fish | Canada | Lake D-3 | -0.85 |  | 1.43 |  | 1.31 |  | *Gantner et al., 2009 EST* |
| Fish | Canada | Lake D-4 | -0.86 |  | 1.39 |  | 1.30 |  | *Gantner et al., 2009 EST* |
| Fish | Canada | Lake D-5 | -0.76 |  | 1.92 |  | 1.66 |  | *Gantner et al., 2009 EST* |
| Fish | Canada | Lake D-6 | -0.67 |  | 2.13 |  | 1.86 |  | *Gantner et al., 2009 EST* |
| Fish | Canada | Lake D-7 | -0.61 |  | 2.03 |  | 1.79 |  | *Gantner et al., 2009 EST* |
| Fish | Canada | Lake Hazen-1 | -1.10 |  | 1.55 |  | 1.55 |  | *Gantner et al., 2009 EST* |
| Fish | Canada | Lake Hazen-2 | -1.12 |  | 1.57 |  | 1.42 |  | *Gantner et al., 2009 EST* |
| Fish | Canada | Lake Hazen-3a | -1.01 |  | 1.81 |  | 1.60 |  | *Gantner et al., 2009 EST* |
| Fish | Canada | Lake Hazen-3b | -1.02 |  | 1.75 |  | 1.57 |  | *Gantner et al., 2009 EST* |
| Fish | Canada | Lake Hazen-3c | -1.04 |  | 1.80 |  | 1.68 |  | *Gantner et al., 2009 EST* |
| Fish | Canada | Lake Hazen-12 | -1.62 |  | 1.27 |  | 1.33 |  | *Gantner et al., 2009 EST* |
| Fish | Canada | Lake Hazen-13 | -1.54 |  | 1.28 |  | 1.31 |  | *Gantner et al., 2009 EST* |
| Fish | Canada | Lake Hazen-14a | -1.25 |  | 1.32 |  | 1.27 |  | *Gantner et al., 2009 EST* |
| Fish | Canada | Lake Hazen-14b | -1.24 |  | 1.34 |  | 1.27 |  | *Gantner et al., 2009 EST* |
| Fish | Canada | Lake Hazen-14c | -1.27 |  | 1.42 |  | 1.35 |  | *Gantner et al., 2009 EST* |
| Fish | Canada | Lake G-1 | -1.35 |  | 1.44 |  | 1.36 |  | *Gantner et al., 2009 EST* |
| Fish | Canada | Lake G-2 | -1.68 |  | 0.86 |  | 0.96 |  | *Gantner et al., 2009 EST* |
| Fish | Canada | Lake G-3 | -1.41 |  | 1.16 |  | 1.18 |  | *Gantner et al., 2009 EST* |
| Fish | Canada | Lake G-5a | -1.34 |  | 1.38 |  | 1.34 |  | *Gantner et al., 2009 EST* |
| Fish | Canada | Lake G-5b | -1.39 |  | 1.29 |  | 1.26 |  | *Gantner et al., 2009 EST* |
| Fish | Canada | Lake G-5c | -1.38 |  | 1.30 |  | 1.24 |  | *Gantner et al., 2009 EST* |
| Fish | Canada | Lake G-6 | -1.60 |  | 0.94 |  | 0.99 |  | *Gantner et al., 2009 EST* |
| Fish | Canada | Amituk lake-1 | -0.74 |  | 0.28 |  | 0.16 |  | *Gantner et al., 2009 EST* |
| Fish | Canada | Amituk lake-3 | -0.49 |  | 0.33 |  | 0.12 |  | *Gantner et al., 2009 EST* |
| Fish | Canada | Amituk lake-4 | -0.70 |  | 0.00 |  | -0.11 |  | *Gantner et al., 2009 EST* |
| Fish | Canada | Amituk lake-5 | -0.86 |  | 0.35 |  | 0.25 |  | *Gantner et al., 2009 EST* |
| Fish | Canada | Amituk lake-6 | -0.71 |  | 0.10 |  | 0.00 |  | *Gantner et al., 2009 EST* |
| Fish | Canada | 9 Mile lake-6 | 0.06 |  | 2.24 |  | 1.61 |  | *Gantner et al., 2009 EST* |
| Fish | Canada | 9 Mile lake-7 | 0.18 |  | 2.20 |  | 1.53 |  | *Gantner et al., 2009 EST* |
| Fish | Canada | 9 Mile lake-8 | 0.05 |  | 1.73 |  | 1.18 |  | *Gantner et al., 2009 EST* |
| Fish | Canada | 9 Mile lake-9 | 0.16 |  | 2.32 |  | 1.69 |  | *Gantner et al., 2009 EST* |
| Fish | Canada | 9 Mile lake-10 | 0.12 |  | 2.14 |  | 1.55 |  | *Gantner et al., 2009 EST* |
| Fish | Canada | Resolute-2 | -0.31 |  | 1.05 |  | 0.78 |  | *Gantner et al., 2009 EST* |
| Fish | Canada | Resolute-3 | -0.21 |  | 1.32 |  | 1.00 |  | *Gantner et al., 2009 EST* |
| Fish | Canada | Resolute-6 | -0.15 |  | 1.38 |  | 1.03 |  | *Gantner et al., 2009 EST* |
| Fish | Canada | Resolute-7a | -0.27 |  | 1.32 |  | 1.03 |  | *Gantner et al., 2009 EST* |
| Fish | Canada | Resolute-7b | -0.27 |  | 1.23 |  | 0.95 |  | *Gantner et al., 2009 EST* |
| Fish | Canada | Resolute-7c | -0.26 |  | 1.35 |  | 1.03 |  | *Gantner et al., 2009 EST* |
| Fish | Canada | Resolute-10 | 0.02 |  | 0.00 |  | -0.02 |  | *Gantner et al., 2009 EST* |
| Fish | Canada | Gavia F-3 | 0.25 |  | 1.77 |  | 1.25 |  | *Gantner et al., 2009 EST* |
| Fish | Canada | Gavia F-4 | 0.24 |  | 1.35 |  | 0.90 |  | *Gantner et al., 2009 EST* |
| Fish | Canada | Gavia F-5 | 0.36 |  | 1.78 |  | 1.28 |  | *Gantner et al., 2009 EST* |
| Fish | Canada | Gavia F-6 | 0.41 |  | 1.41 |  | 0.91 |  | *Gantner et al., 2009 EST* |
| Fish | Canada | Gavia F-7 | 0.27 |  | 1.31 |  | 0.96 |  | *Gantner et al., 2009 EST* |
| Fish | Canada | Notgordie-1 | 0.06 |  | 1.88 |  | 1.35 |  | *Gantner et al., 2009 EST* |
| Fish | Canada | Notgordie-2a | -0.01 |  | 1.44 |  | 1.04 |  | *Gantner et al., 2009 EST* |
| Fish | Canada | Notgordie-2b | 0.06 |  | 1.48 |  | 1.05 |  | *Gantner et al., 2009 EST* |
| Fish | Canada | Notgordie-3 | 0.09 |  | 2.02 |  | 1.46 |  | *Gantner et al., 2009 EST* |
| Fish | Canada | Notgordie-4 | 0.16 |  | 2.37 |  | 1.73 |  | *Gantner et al., 2009 EST* |
| Fish | Canada | Notgordie-5 | 0.06 |  | 2.28 |  | 1.72 |  | *Gantner et al., 2009 EST* |
| Fish | Canada | Little N-4 | 0.20 |  | 1.21 |  | 0.86 |  | *Gantner et al., 2009 EST* |
| Fish | Canada | Little N-5 | 0.15 |  | 1.24 |  | 0.90 |  | *Gantner et al., 2009 EST* |
| Fish | Canada | Little N-6 | 0.04 |  | 1.03 |  | 0.79 |  | *Gantner et al., 2009 EST* |
| Fish | Canada | Little N-7a | 0.03 |  | 1.42 |  | 1.14 |  | *Gantner et al., 2009 EST* |
| Fish | Canada | Little N-7b | 0.03 |  | 1.30 |  | 1.00 |  | *Gantner et al., 2009 EST* |
| Fish | Canada | Little N-9 | 0.16 |  | 1.38 |  | 1.05 |  | *Gantner et al., 2009 EST* |
| Fish | Canada | Pingualuk-3a | 1.24 |  | 5.17 |  | 3.82 |  | *Gantner et al., 2009 EST* |
| Fish | Canada | Pingualuk-3b | 1.28 |  | 5.22 |  | 3.89 |  | *Gantner et al., 2009 EST* |
| Fish | Canada | Pingualuk-3c | 1.28 |  | 5.18 |  | 3.87 |  | *Gantner et al., 2009 EST* |
| Fish | Canada | Pingualuk-4 | 1.20 |  | 5.13 |  | 3.78 |  | *Gantner et al., 2009 EST* |
| Fish | Canada | Pingualuk-5 | 1.13 |  | 4.91 |  | 3.67 |  | *Gantner et al., 2009 EST* |
| Fish | Canada | Pingualuk-6 | 1.25 |  | 4.85 |  | 3.59 |  | *Gantner et al., 2009 EST* |
| Fish | Canada | Pingualuk-7 | 1.15 |  | 4.90 |  | 3.60 |  | *Gantner et al., 2009 EST* |
| Fish | Canada | DORM-1-1 | 0.44 |  | 1.26 |  | 0.86 |  | *Gantner et al., 2009 EST* |
| Fish | Canada | DORM-1-2 | 0.06 |  | 1.29 |  | 1.03 |  | *Gantner et al., 2009 EST* |
| Fish | Canada | DORM-1-3 | 0.07 |  | 1.18 |  | 0.93 |  | *Gantner et al., 2009 EST* |
| Chironomids | Canada | Lake Hazen | -1.59 |  | 0.32 |  | 0.16 |  | *Gantner et al., 2009 EST* |
| Chironomids | Canada | Resolute L | -0.32 |  | 1.31 |  | 0.99 |  | *Gantner et al., 2009 EST* |
| Zooplankton | Canada | Lake Hazen | -0.46 |  | 2.30 |  | 1.85 |  | *Gantner et al., 2009 EST* |
| Zooplankton | Canada | Resolute L-2005 | -0.23 |  | 3.40 |  | 2.67 |  | *Gantner et al., 2009 EST* |
| Zooplankton | Canada | Resolute L-2006 | -0.13 |  | 2.20 |  | 1.55 |  | *Gantner et al., 2009 EST* |
| Zooplankton | Canada | Resolute L-2007 | -0.15 |  | 2.88 |  | 2.18 |  | *Gantner et al., 2009 EST* |
| Zooplankton | Canada | Gavia FL | -0.10 |  | 1.51 |  | 1.10 |  | *Gantner et al., 2009 EST* |
| Zooplankton | Canada | Notgordie L | 0.12 |  | 3.05 |  | 2.27 |  | *Gantner et al., 2009 EST* |
| Fish | Lake Baikal | P-Ba1 | -0.33 |  | 1.05 |  | 0.79 |  | *Perrot et al., 2011* |
| Fish | Lake Baikal | P-Ba2 | -0.57 |  | 0.99 |  | 0.77 |  | *Perrot et al., 2011* |
| Fish | Lake Baikal | P-Ba3 | -0.50 |  | 1.30 |  | 1.02 |  | *Perrot et al., 2011* |
| Fish | Lake Baikal | P-Ba4 | -0.55 |  | 1.39 |  | 1.06 |  | *Perrot et al., 2011* |
| Fish | Lake Baikal | P-Ba5 | -0.47 |  | 1.18 |  | 0.89 |  | *Perrot et al., 2011* |
| Fish | Lake Baikal | P-Ba6 | -0.44 |  | 0.93 |  | 0.71 |  | *Perrot et al., 2011* |
| Fish | Lake Baikal | P-Ba7 | -0.38 |  | 1.04 |  | 0.79 |  | *Perrot et al., 2011* |
| Fish | Lake Baikal | P-Ba8 | -0.52 |  | 1.65 |  | 1.24 |  | *Perrot et al., 2011* |
| Fish | Lake Baikal | P-Ba9 | -0.47 |  | 0.87 |  | 0.73 |  | *Perrot et al., 2011* |
| Fish | Lake Baikal | P-Ba10 | -0.53 |  | 1.00 |  | 0.81 |  | *Perrot et al., 2011* |
| Fish | Lake Baikal | P-Ba11 | -0.56 |  | 1.32 |  | 1.05 |  | *Perrot et al., 2011* |
| Fish | Lake Baikal | P-Ba12 | -0.47 |  | 0.99 |  | 0.81 |  | *Perrot et al., 2011* |
| Fish | Lake Baikal | P-Br1 | -0.90 |  | 0.20 |  | 0.13 |  | *Perrot et al., 2011* |
| Fish | Lake Baikal | P-Br2 | -0.64 |  | 0.48 |  | 0.34 |  | *Perrot et al., 2011* |
| Fish | Lake Baikal | P-Br3 | -0.77 |  | 0.52 |  | 0.40 |  | *Perrot et al., 2011* |
| Fish | Lake Baikal | P-Br4 | -0.65 |  | 0.45 |  | 0.36 |  | *Perrot et al., 2011* |
| Fish | Lake Baikal | P-Br5 | -0.51 |  | 0.67 |  | 0.55 |  | *Perrot et al., 2011* |
| Fish | Lake Baikal | P-Br6 | -0.51 |  | 0.30 |  | 0.20 |  | *Perrot et al., 2011* |
| Fish | Lake Baikal | P-Br7 | -0.43 |  | 0.35 |  | 0.25 |  | *Perrot et al., 2011* |
| Fish | Lake Baikal | P-Br8 | -0.85 |  | 0.34 |  | 0.24 |  | *Perrot et al., 2011* |
| Fish | Lake Baikal | P-Br9 | -0.56 |  | 0.43 |  | 0.32 |  | *Perrot et al., 2011* |
| Fish | Lake Baikal | P-Br10 | -0.26 |  | 0.56 |  | 0.40 |  | *Perrot et al., 2011* |
| Fish | Lake Baikal | P-Br11 | -0.39 |  | 0.93 |  | 0.75 |  | *Perrot et al., 2011* |
| Fish | Lake Baikal | P-Br12 | -0.16 |  | 1.04 |  | 0.79 |  | *Perrot et al., 2011* |
| Fish | Lake Baikal | R-Ba1 | -0.61 |  | 0.49 |  | 0.37 |  | *Perrot et al., 2011* |
| Fish | Lake Baikal | R-Ba2 | -0.65 |  | 0.51 |  | 0.41 |  | *Perrot et al., 2011* |
| Fish | Lake Baikal | R-Ba3 | -0.51 |  | 0.58 |  | 0.47 |  | *Perrot et al., 2011* |
| Fish | Lake Baikal | R-Ba4 | -0.47 |  | 0.26 |  | 0.20 |  | *Perrot et al., 2011* |
| Fish | Lake Baikal | R-Ba5 | -0.67 |  | 0.56 |  | 0.45 |  | *Perrot et al., 2011* |
| Fish | Lake Baikal | R-Ba6 | -0.60 |  | 0.52 |  | 0.37 |  | *Perrot et al., 2011* |
| Fish | Lake Baikal | R-Ba7 | -0.73 |  | 0.70 |  | 0.52 |  | *Perrot et al., 2011* |
| Fish | Lake Baikal | R-Ba8 | -0.58 |  | 0.46 |  | 0.35 |  | *Perrot et al., 2011* |
| Fish | Lake Baikal | R-Ba9 | -0.61 |  | 0.79 |  | 0.59 |  | *Perrot et al., 2011* |
| Fish | Lake Baikal | R-Ba10 | -0.62 |  | 0.71 |  | 0.54 |  | *Perrot et al., 2011* |
| Fish | Lake Baikal | R-Ba11 | -0.67 |  | 0.65 |  | 0.50 |  | *Perrot et al., 2011* |
| Fish | Lake Baikal | R-Ba12 | -0.61 |  | 0.75 |  | 0.53 |  | *Perrot et al., 2011* |
| Fish | Lake Baikal | R-Br1 | -0.80 |  | 0.41 |  | 0.30 |  | *Perrot et al., 2011* |
| Fish | Lake Baikal | R-Br2 | -0.40 |  | 0.58 |  | 0.44 |  | *Perrot et al., 2011* |
| Fish | Lake Baikal | R-Br3 | -0.35 |  | 1.09 |  | 0.75 |  | *Perrot et al., 2011* |
| Fish | Lake Baikal | R-Br4 | -0.33 |  | 0.65 |  | 0.49 |  | *Perrot et al., 2011* |
| Fish | Lake Baikal | R-Br5 | -0.31 |  | 0.93 |  | 0.70 |  | *Perrot et al., 2011* |
| Fish | Lake Baikal | R-Br6 | -0.23 |  | 1.24 |  | 0.94 |  | *Perrot et al., 2011* |
| Fish | Lake Baikal | R-Br7 | -0.33 |  | 0.94 |  | 0.71 |  | *Perrot et al., 2011* |
| Fish | Lake Baikal | R-Br8 | -0.22 |  | 1.68 |  | 1.28 |  | *Perrot et al., 2011* |
| Fish | Lake Baikal | R-Br9 | -0.35 |  | 1.87 |  | 1.39 |  | *Perrot et al., 2011* |
| Fish | Lake Baikal | R-Br10 | -0.39 |  | 1.73 |  | 1.39 |  | *Perrot et al., 2011* |
| Fish | Lake Baikal | R-Br11 | -0.38 |  | 1.49 |  | 1.10 |  | *Perrot et al., 2011* |
| Fish | Lake Baikal | R-Br12 | -0.31 |  | 1.67 |  | 1.25 |  | *Perrot et al., 2011* |
| Pllankton | Lake Baikal | Pl-Baikal | -0.90 |  | 1.53 |  | 1.36 |  | *Perrot et al., 2011* |
| Pllankton | Lake Baikal | Pl-Bratsk | -0.75 |  | 1.28 |  | 1.08 |  | *Perrot et al., 2011* |
| Fish |  | DORM-2 | 0.18 |  | 1.07 |  | 0.88 |  | *Bergquist and Blum, 2007* |
| Fish |  | DOLT-2 | -0.65 |  | 0.71 |  | 0.59 |  | *Bergquist and Blum, 2007* |
| Fish | Lake Michigan | 16-S | 0.35 |  | 3.08 |  | 2.40 |  | *Bergquist and Blum, 2007* |
| Fish | Lake Michigan | 16-S | 0.55 |  |  |  | 2.44 |  | *Bergquist and Blum, 2007* |
| Fish | Lake Michigan | 18-S-a | 1.02 |  | 4.62 |  | 3.64 |  | *Bergquist and Blum, 2007* |
| Fish | Lake Michigan | 18-S-b | 1.17 |  | 4.64 |  | 3.61 |  | *Bergquist and Blum, 2007* |
| Fish | Lake Michigan | 20-S | 0.86 |  | 3.87 |  | 3.09 |  | *Bergquist and Blum, 2007* |
| Fish | Lake Michigan | 22-S | 1.13 |  | 4.59 |  | 3.55 |  | *Bergquist and Blum, 2007* |
| Fish | Lake Michigan | 23-S | 0.54 |  | 3.05 |  | 2.42 |  | *Bergquist and Blum, 2007* |
| Fish | Lake Michigan | 26-S | 1.03 |  | 4.39 |  | 3.44 |  | *Bergquist and Blum, 2007* |
| Fish | Lake Michigan | BR-1-S | -0.29 |  | 3.13 |  | 2.47 |  | *Bergquist and Blum, 2007* |
| Fish | Lake Michigan | BR-6-S | 0.55 |  | 2.87 |  | 2.23 |  | *Bergquist and Blum, 2007* |
| Fish | Lake Michigan | BR-11-S-a | 0.25 |  | 2.75 |  | 2.21 |  | *Bergquist and Blum, 2007* |
| Fish | Lake Michigan | BR-11-S-b | 0.21 |  | 2.73 |  | 2.16 |  | *Bergquist and Blum, 2007* |
| Fish | Lake Michigan | Bur100 | 0.56 |  | 3.41 |  | 2.60 |  | *Bergquist and Blum, 2007* |
| Fish | Lake Michigan | Bur100 | 0.24 |  | 3.39 |  | 2.66 |  | *Bergquist and Blum, 2007* |
| Fish | Lake Michigan | Bur111 | 0.78 |  | 3.66 |  | 2.83 |  | *Bergquist and Blum, 2007* |
| Fish | Lake Michigan | Bur111 | 0.72 |  | 3.78 |  | 2.92 |  | *Bergquist and Blum, 2007* |
| Fish | Lake Michigan | Bur121 | 0.01 |  | 3.68 |  | 2.81 |  | *Bergquist and Blum, 2007* |
| Fish | New England | ELYP120-M | -1.40 |  |  |  | 2.25 |  | *Bergquist and Blum, 2007* |
| Fish | New England | ELYP30-M | -2.33 |  |  |  | 1.65 |  | *Bergquist and Blum, 2007* |
| Fish | New England | HPCP200-M | -0.37 |  |  |  | 0.75 |  | *Bergquist and Blum, 2007* |
| Fish | New England | HPCP200-M | -0.30 |  | 0.96 |  | 0.66 |  | *Bergquist and Blum, 2007* |
| Fish | New England | HPCP49-M | -1.81 |  |  |  | 0.88 |  | *Bergquist and Blum, 2007* |
| Fish | New England | HPCP112-M | -1.83 |  |  |  | 0.91 |  | *Bergquist and Blum, 2007* |
| Fish | New England | HPCP141-M | -1.70 |  |  |  | 0.83 |  | *Bergquist and Blum, 2007* |
| Fish | New England | HPPS31-M | -1.00 |  | 0.29 |  | 0.22 |  | *Bergquist and Blum, 2007* |
| Fish | Puerto Salinas | Id44 | -0.79 |  | 0.01 |  | -0.09 |  | *Laffont et al., 2009 EST* |
| Fish | Puerto Salinas | Id123 | -0.59 |  | 0.27 |  | 0.19 |  | *Laffont et al., 2009 EST* |
| Fish | Puerto Salinas | Id128 | -0.65 |  | -0.09 |  | -0.06 |  | *Laffont et al., 2009 EST* |
| Fish | Puerto Salinas | Id282 | -0.67 |  | -0.08 |  | -0.14 |  | *Laffont et al., 2009 EST* |
| Fish | Granja lake | Y46 | -0.87 |  | 0.50 |  | 0.34 |  | *Laffont et al., 2009 EST* |
| Fish | Granja lake | Y17 | -0.89 |  | 0.55 |  | 0.38 |  | *Laffont et al., 2009 EST* |
| Fish | Granja lake | Y205 | -0.61 |  | 0.50 |  | 0.37 |  | *Laffont et al., 2009 EST* |
| Fish | Granja lake | Id26 | -0.92 |  | 0.36 |  | 0.27 |  | *Laffont et al., 2009 EST* |
| Fish | Granja lake | Y237 | -0.81 |  | 0.40 |  | 0.27 |  | *Laffont et al., 2009 EST* |
| Fish | Granja lake | Id97 | -0.61 |  | 0.42 |  | 0.24 |  | *Laffont et al., 2009 EST* |
| Fish | Granja lake | Y59 | -0.53 |  | 0.49 |  | 0.28 |  | *Laffont et al., 2009 EST* |
| Fish | Granja lake | Y292 | -0.54 |  | 0.52 |  | 0.31 |  | *Laffont et al., 2009 EST* |
| Fish | Granja lake | Y52 | -0.40 |  | 0.52 |  | 0.29 |  | *Laffont et al., 2009 EST* |
| Fish | Granja lake | Y37 | -0.44 |  | 0.53 |  | 0.36 |  | *Laffont et al., 2009 EST* |
| Fish | Granja lake | Id106 | -0.63 |  | 0.53 |  | 0.36 |  | *Laffont et al., 2009 EST* |
| Fish | Granja lake | Y72 | -0.48 |  | 0.41 |  | 0.27 |  | *Laffont et al., 2009 EST* |
| Fish | Granja lake | Y26 | -0.47 |  | 0.55 |  | 0.38 |  | *Laffont et al., 2009 EST* |
| Fish | Granja lake | Y70 | -0.55 |  | 0.44 |  | 0.29 |  | *Laffont et al., 2009 EST* |
| Fish | Granja lake | Id235 | -0.61 |  | 0.45 |  | 0.29 |  | *Laffont et al., 2009 EST* |
| Fish | Granja lake | Y326 | -0.45 |  | 0.52 |  | 0.36 |  | *Laffont et al., 2009 EST* |
| Fish | Itenez river basin | IT-P231 | -0.70 |  | 0.11 |  | 0.05 |  | *Laffont et al., 2009 EST* |
| Fish | Itenez river basin | IT-P237 | -0.48 |  | 0.25 |  | 0.22 |  | *Laffont et al., 2009 EST* |
| Fish | Itenez river basin | IT-P265 | -0.40 |  | 0.31 |  | 0.27 |  | *Laffont et al., 2009 EST* |
| Fish | Itenez river basin | IT-P264 | -0.53 |  | 0.02 |  | -0.02 |  | *Laffont et al., 2009 EST* |
| Fish | Itenez river basin | IT-P240 | -0.66 |  | -0.08 |  | 0.12 |  | *Laffont et al., 2009 EST* |
| Fish | Itenez river basin | IT-P366 | -0.38 |  | 0.30 |  | 0.29 |  | *Laffont et al., 2009 EST* |
| Fish | Itenez river basin | IT-P351 | -0.68 |  | 0.08 |  | 0.06 |  | *Laffont et al., 2009 EST* |
| Fish | Itenez river basin | IT-P376 | -0.68 |  | 0.10 |  | 0.08 |  | *Laffont et al., 2009 EST* |
| Fish | Itenez river basin | IT-P372 | -0.55 |  | 0.12 |  | 0.08 |  | *Laffont et al., 2009 EST* |
| Fish | Itenez river basin | IT-P310 | -0.61 |  | 0.16 |  | 0.11 |  | *Laffont et al., 2009 EST* |
|  |  |  |  |  |  |  |  |  |  |
| Lichen | Alberta, Canada | 646 | -1.93 |  | 0.10 |  | 0.06 |  | *Blum et al., 2012* |
| Lichen | Alberta, Canada | 602 | -1.87 |  | 0.05 |  | -0.02 |  | *Blum et al., 2012* |
| Lichen | Alberta, Canada | 36 | -2.04 |  | -0.19 |  | -0.26 |  | *Blum et al., 2012* |
| Lichen | Alberta, Canada | 12 | -1.95 |  | -0.29 |  | -0.41 |  | *Blum et al., 2012* |
| Lichen | Alberta, Canada | 690 | -2.19 |  | -0.19 |  | -0.31 |  | *Blum et al., 2012* |
| Lichen | Alberta, Canada | 45 | -1.62 |  | -0.05 |  | -0.22 |  | *Blum et al., 2012* |
| Lichen | Alberta, Canada | 45 | -1.86 |  | -0.01 |  | -0.19 |  | *Blum et al., 2012* |
| Lichen | Alberta, Canada | 648 | -1.84 |  | -0.07 |  | -0.13 |  | *Blum et al., 2012* |
| Lichen | Alberta, Canada | 660 | -2.23 |  | -0.20 |  | -0.28 |  | *Blum et al., 2012* |
| Lichen | Alberta, Canada | 33 | -1.91 |  | -0.01 |  | -0.07 |  | *Blum et al., 2012* |
| Lichen | Alberta, Canada | 32 | -1.81 |  | -0.27 |  | -0.37 |  | *Blum et al., 2012* |
| Lichen | Alberta, Canada | 592 | -1.67 |  | -0.16 |  | -0.26 |  | *Blum et al., 2012* |
| Lichen | Alberta, Canada | 744 | -1.66 |  | -0.17 |  | -0.37 |  | *Blum et al., 2012* |
| Lichen | Alberta, Canada | 1 | -1.90 |  | -0.20 |  | -0.34 |  | *Blum et al., 2012* |
| Lichen | Alberta, Canada | 604 | -1.81 |  | 0.33 |  | 0.16 |  | *Blum et al., 2012* |
| Lichen | Alberta, Canada | 2 | -1.82 |  | -0.28 |  | -0.36 |  | *Blum et al., 2012* |
| Lichen | Alberta, Canada | 20 | -1.67 |  | -0.19 |  | -0.33 |  | *Blum et al., 2012* |
| Lichen | Alberta, Canada | 20 | -1.72 |  | -0.26 |  | -0.35 |  | *Blum et al., 2012* |
| Lichen | Alberta, Canada | 774 | -1.82 |  | -0.30 |  | -0.37 |  | *Blum et al., 2012* |
| Lichen | Alberta, Canada | 116 | -1.87 |  | -0.20 |  | -0.34 |  | *Blum et al., 2012* |
| Lichen | Alberta, Canada | 114 | -1.72 |  | -0.19 |  | -0.33 |  | *Blum et al., 2012* |
| Lichen | Alberta, Canada | 76 | -1.91 |  | -0.17 |  | -0.31 |  | *Blum et al., 2012* |
| Lichen | Alberta, Canada | 88 | -1.93 |  | -0.43 |  | -0.52 |  | *Blum et al., 2012* |
| Lichen | Alberta, Canada | 257 | -1.93 |  | -0.37 |  | -0.45 |  | *Blum et al., 2012* |
| Lichen | Alberta, Canada | 325 | -1.78 |  | -0.31 |  | -0.45 |  | *Blum et al., 2012* |
| Lichen | Alberta, Canada | 597 | -1.81 |  | -0.37 |  | -0.50 |  | *Blum et al., 2012* |
| Lichen | Alberta, Canada | 207 | -1.76 |  | -0.35 |  | -0.49 |  | *Blum et al., 2012* |
| Lichen | Alberta, Canada | 184 | -2.29 |  | -0.31 |  | -0.45 |  | *Blum et al., 2012* |
| Lichen | Alberta, Canada | 304 | -1.75 |  | -0.36 |  | -0.44 |  | *Blum et al., 2012* |
| Lichen | Alberta, Canada | 463 | -1.41 |  | -0.26 |  | -0.41 |  | *Blum et al., 2012* |
| Lichen | Alberta, Canada | 497 | -1.91 |  | -0.38 |  | -0.49 |  | *Blum et al., 2012* |
| Lichen | Alberta, Canada | 421 | -1.86 |  | -0.48 |  | -0.54 |  | *Blum et al., 2012* |
| Lichen | Alberta, Canada | 421 | -1.81 |  | -0.40 |  | -0.52 |  | *Blum et al., 2012* |
| Lichen | Alberta, Canada | 555 | -1.58 |  | -0.39 |  | -0.51 |  | *Blum et al., 2012* |
| Lichen | Alberta, Canada | 540 | -2.66 |  | -0.39 |  | -0.44 |  | *Blum et al., 2012* |
| Lichen | Alberta, Canada | 569 | -1.70 |  | -0.55 |  | -0.63 |  | *Blum et al., 2012* |
| Lichen | Alberta, Canada | 548 | -2.22 |  | -0.29 |  | -0.44 |  | *Blum et al., 2012* |
| Lichen | Alberta, Canada | 588 | -1.87 |  | -0.40 |  | -0.47 |  | *Blum et al., 2012* |
| Foliage and leaf litter | California | Senesced foliage | -2.53 |  | -0.37 |  | -0.35 |  | *Tsui et al., ES&T, 2012* |
| Foliage and leaf litter | California | Decomposed bark | -2.12 |  | -0.36 |  | -0.33 |  | *Tsui et al., ES&T, 2012* |
| Lichen | France | Fr01 29 | -0.87 |  | -0.40 |  | -0.46 |  | *Carignan et al., 2009* |
| Lichen | France | Fr03 29 | -1.35 |  | -0.34 |  | -0.39 |  | *Carignan et al., 2009* |
| Lichen | France | Fr06 29a | -1.01 |  | -0.39 |  | -0.42 |  | *Carignan et al., 2009* |
| Lichen | France | Fr01 14A | -1.21 |  | -0.41 |  | -0.52 |  | *Carignan et al., 2009* |
| Lichen | France | Fr03 14A | -1.23 |  | -0.43 |  | -0.46 |  | *Carignan et al., 2009* |
| Lichen | France | Fr03 18 | -1.11 |  | -0.34 |  | -0.49 |  | *Carignan et al., 2009* |
| Lichen | France | Fr06 18A | -0.75 |  | -0.32 |  | -0.41 |  | *Carignan et al., 2009* |
| Lichen | France | Fr01 27 | 1.30 |  | -0.62 |  | -0.62 |  | *Carignan et al., 2009* |
| Lichen | France | bis | 1.18 |  | -0.65 |  | -0.64 |  | *Carignan et al., 2009* |
| Lichen | France | Fr03 27 | 1.41 |  | -0.38 |  | -0.41 |  | *Carignan et al., 2009* |
| Lichen | France | bis | 1.40 |  | -0.42 |  | -0.39 |  | *Carignan et al., 2009* |
| Lichen | France | Fr06 27 | 1.50 |  | -0.51 |  | -0.55 |  | *Carignan et al., 2009* |
| Lichen | France | bis | 1.49 |  | -0.56 |  | -0.53 |  | *Carignan et al., 2009* |
| Lichen | France | Fr03 12B | -1.61 |  | -0.36 |  | -0.50 |  | *Carignan et al., 2009* |
| Lichen | France | Fr03 10A | -1.74 |  | -0.55 |  | -0.59 |  | *Carignan et al., 2009* |
| Lichen | France | Fr03 07 | -1.10 |  | -0.43 |  | -0.49 |  | *Carignan et al., 2009* |
| Lichen | France | Fr06 07 | -0.92 |  | -0.44 |  | -0.50 |  | *Carignan et al., 2009* |
| Lichen | France | Fr03 35 | -0.90 |  | -0.61 |  | -0.65 |  | *Carignan et al., 2009* |
| Lichen | France | Fr06 35b | -1.21 |  | -0.51 |  | -0.56 |  | *Carignan et al., 2009* |
| Lichen | France | Fr01 21 | -1.42 |  | -0.22 |  | -0.30 |  | *Carignan et al., 2009* |
| Lichen | France | Fr06 21 | -1.30 |  | -0.24 |  | -0.35 |  | *Carignan et al., 2009* |
| Lichen | France | Fr03 24 | -2.18 |  | -0.56 |  | -0.58 |  | *Carignan et al., 2009* |
| Lichen | France | Fr06 24 | -1.09 |  | -0.41 |  | -0.51 |  | *Carignan et al., 2009* |
| Lichen | NE France | UA-21A | -1.42 |  | -0.22 |  | -0.30 |  | *Estrade et al., 2010 EST* |
| Lichen | NE France | UA-22 | -1.30 |  | -0.24 |  | -0.35 |  | *Estrade et al., 2010 EST* |
| Lichen | NE France | UA-17 | -0.99 |  | -0.31 |  | -0.41 |  | *Estrade et al., 2010 EST* |
| Lichen | NE France | UA-18 | -1.11 |  | -0.34 |  | -0.49 |  | *Estrade et al., 2010 EST* |
| Lichen | NE France | UA-18a | -0.75 |  | -0.32 |  | -0.41 |  | *Estrade et al., 2010 EST* |
| Lichen | NE France | UA-18 | -0.97 |  | -0.28 |  | -0.41 |  | *Estrade et al., 2010 EST* |
| Lichen | NE France | UA-16 | -0.65 |  | -0.26 |  | -0.36 |  | *Estrade et al., 2010 EST* |
| Lichen | NE France | UA-15 | -0.42 |  | -0.29 |  | -0.37 |  | *Estrade et al., 2010 EST* |
| Lichen | NE France | SA-07 | -1.10 |  | -0.43 |  | -0.49 |  | *Estrade et al., 2010 EST* |
| Lichen | NE France | SA-07 | -0.92 |  | -0.44 |  | -0.50 |  | *Estrade et al., 2010 EST* |
| Lichen | NE France | SA-07 | -0.79 |  | -0.28 |  | -0.35 |  | *Estrade et al., 2010 EST* |
| Lichen | NE France | SA-12B | -1.61 |  | -0.36 |  | -0.50 |  | *Estrade et al., 2010 EST* |
| Lichen | NE France | SA-12 | -1.58 |  | -0.21 |  | -0.27 |  | *Estrade et al., 2010 EST* |
| Lichen | NE France | SA-13 | -0.67 |  | -0.38 |  | -0.49 |  | *Estrade et al., 2010 EST* |
| Lichen | NE France | SA-14a | -1.21 |  | -0.41 |  | -0.52 |  | *Estrade et al., 2010 EST* |
| Lichen | NE France | SA-14a | -1.23 |  | -0.43 |  | -0.46 |  | *Estrade et al., 2010 EST* |
| Lichen | NE France | SA-14 | -0.79 |  | -0.42 |  | -0.47 |  | *Estrade et al., 2010 EST* |
| Lichen | NE France | SA-23 | -0.62 |  | -0.40 |  | -0.40 |  | *Estrade et al., 2010 EST* |
| Lichen | NE France | SA-29 | -0.87 |  | -0.40 |  | -0.46 |  | *Estrade et al., 2010 EST* |
| Lichen | NE France | SA-29 | -1.35 |  | -0.34 |  | -0.39 |  | *Estrade et al., 2010 EST* |
| Lichen | NE France | SA-29a | -1.01 |  | -0.39 |  | -0.42 |  | *Estrade et al., 2010 EST* |
| Lichen | NE France | SA-37 | -0.95 |  | -0.36 |  | -0.48 |  | *Estrade et al., 2010 EST* |
| Lichen | NE France | SA-24 | -1.09 |  | -0.41 |  | -0.51 |  | *Estrade et al., 2010 EST* |
| Lichen | NE France | SA-24 | -1.14 |  | -0.41 |  | -0.41 |  | *Estrade et al., 2010 EST* |
| Lichen | NE France | SA-19 | -1.26 |  | -0.42 |  | -0.53 |  | *Estrade et al., 2010 EST* |
| Lichen | NE France | RA-10A | -1.74 |  | -0.55 |  | -0.59 |  | *Estrade et al., 2010 EST* |
| Lichen | NE France | RA-10 | -1.49 |  | -0.44 |  | -0.49 |  | *Estrade et al., 2010 EST* |
| Lichen | NE France | RA-35 | -0.89 |  | -0.46 |  | -0.55 |  | *Estrade et al., 2010 EST* |
| Lichen | NE France | RA-35 | -0.90 |  | -0.61 |  | -0.65 |  | *Estrade et al., 2010 EST* |
| Lichen | NE France | RA-35 | -1.07 |  | -0.55 |  | -0.60 |  | *Estrade et al., 2010 EST* |
| Lichen | NE France | RA-35B | -1.21 |  | -0.51 |  | -0.56 |  | *Estrade et al., 2010 EST* |
| Lichen | NE France | RA-03 | -0.99 |  | -0.47 |  | -0.49 |  | *Estrade et al., 2010 EST* |
| Lichen | NE France | RA-01 | -1.49 |  | -0.40 |  | -0.41 |  | *Estrade et al., 2010 EST* |
| Lichen | NE France | RA-01 | -1.64 |  | -0.53 |  | -0.55 |  | *Estrade et al., 2010 EST* |
| Lichen | NE France | I-43 | -1.90 |  | -0.16 |  | -0.18 |  | *Estrade et al., 2010 EST* |
| Lichen | NE France | I-44 | -1.89 |  | -0.13 |  | -0.13 |  | *Estrade et al., 2010 EST* |
| Lichen | NE France | IV-45 | -1.75 |  | -0.23 |  | -0.27 |  | *Estrade et al., 2010 EST* |
| Lichen | NE France | IV-46 | -2.22 |  | -0.27 |  | -0.32 |  | *Estrade et al., 2010 EST* |
| Lichen | NE France | IV-47 | -2.19 |  | -0.30 |  | -0.28 |  | *Estrade et al., 2010 EST* |
| Lichen | NE France | IV-47 | -2.13 |  | -0.28 |  | -0.30 |  | *Estrade et al., 2010 EST* |
| Lichen | NE France | IV-48 | -2.07 |  | -0.29 |  | -0.33 |  | *Estrade et al., 2010 EST* |
| Lichen | NE France | IV-49 | -1.94 |  | -0.39 |  | -0.41 |  | *Estrade et al., 2010 EST* |
| Lichen | NE France | CP-27 | 1.24 |  | -0.64 |  | -0.63 |  | *Estrade et al., 2010 EST* |
| Lichen | NE France | CP-27 | 1.40 |  | -0.40 |  | -0.40 |  | *Estrade et al., 2010 EST* |
| Lichen | NE France | CP-27 | 1.50 |  | -0.54 |  | -0.54 |  | *Estrade et al., 2010 EST* |
| Lichen | NE France | CP-27 | 1.37 |  | -0.38 |  | -0.34 |  | *Estrade et al., 2010 EST* |
| Lichen | NE France | SA/ACP-38 | -0.99 |  | -0.42 |  | -0.46 |  | *Estrade et al., 2010 EST* |
| Lichen | NE France | SA/ACP-39 | -1.21 |  | -0.36 |  | -0.38 |  | *Estrade et al., 2010 EST* |
| Lichen | NE France | SA/ACP-40 | -1.11 |  | -0.49 |  | -0.49 |  | *Estrade et al., 2010 EST* |
| Lichen | NE France | SA/ACP-41 | -1.25 |  | -0.42 |  | -0.52 |  | *Estrade et al., 2010 EST* |
| Lichen | NE France | SA/ACP-42 | 0.46 |  | -0.42 |  | -0.44 |  | *Estrade et al., 2010 EST* |
| Lichen | Canada | Lac koury nior | -0.29 |  | -0.59 |  | -0.65 |  | *Carignan et al., 2009* |
| Lichen | Canada | Chisasib nior | -0.29 |  | -0.77 |  | -0.83 |  | *Carignan et al., 2009* |
| Lichen | Canada | Opinaca vert | -0.98 |  | -0.53 |  | -0.58 |  | *Carignan et al., 2009* |
| Lichen | Canada | D24 sud vert | 0.00 |  | -0.95 |  | -1.03 |  | *Carignan et al., 2009* |
| Lichen | Canada | Lac Evans nior | -0.82 |  | -0.85 |  | -0.88 |  | *Carignan et al., 2009* |
| Lichen | Canada | Lacorne | -2.04 |  | -0.24 |  | -0.51 |  | *Carignan et al., 2009* |
| Rice plant | Wanshan | rice seed-WK | -2.33 |  | -0.06 |  | -0.08 |  | *Yin et al., EST 2013* |
| Rice plant | Wanshan | rice seed-WK | -2.24 |  | -0.05 |  | -0.04 |  | *Yin et al., EST 2013* |
| Rice plant | Wanshan | rice seed-WK | -2.15 |  | -0.07 |  | -0.05 |  | *Yin et al., EST 2013* |
| Rice plant | Wanshan | rice root-WK | -1.08 |  | -0.02 |  | -0.01 |  | *Yin et al., EST 2013* |
| Rice plant | Wanshan | rice root-WK | -0.89 |  | -0.03 |  | -0.03 |  | *Yin et al., EST 2013* |
| Rice plant | Wanshan | rice root-WK | -0.92 |  | -0.03 |  | -0.04 |  | *Yin et al., EST 2013* |
| Rice plant | Wanshan | rice stem-WK | -2.70 |  | -0.12 |  | -0.11 |  | *Yin et al., EST 2013* |
| Rice plant | Wanshan | rice stem-WK | -2.82 |  | -0.16 |  | -0.15 |  | *Yin et al., EST 2013* |
| Rice plant | Wanshan | rice stem-WK | -2.65 |  | -0.13 |  | -0.13 |  | *Yin et al., EST 2013* |
| Rice plant | Wanshan | rice leave-WK | -3.38 |  | -0.37 |  | -0.35 |  | *Yin et al., EST 2013* |
| Rice plant | Wanshan | rice leave-WK | -2.97 |  | -0.23 |  | -0.21 |  | *Yin et al., EST 2013* |
| Rice plant | Wanshan | rice leave-WK | -2.88 |  | -0.28 |  | -0.23 |  | *Yin et al., EST 2013* |
| Rice plant | Wanshan | GOLD.-WK-1 | 0.69 |  | -0.21 |  | -0.23 |  | *Yin et al., EST 2013* |
| Rice plant | Wanshan | GOLD.-WK-2 | 0.24 |  | -0.29 |  | -0.29 |  | *Yin et al., EST 2013* |
| Rice plant | Wanshan | BB-WK-1 | -2.26 |  | -0.24 |  | -0.24 |  | *Yin et al., EST 2013* |
| Rice plant | Wanshan | BB-WK-2 | -1.85 |  | -0.26 |  | -0.26 |  | *Yin et al., EST 2013* |
| Rice plant | Wanshan | Lichen-WK-1 | -2.30 |  | -0.22 |  | -0.21 |  | *Yin et al., EST 2013* |
| Rice plant | Wanshan | Lichen-WK-2 | -1.83 |  | -0.31 |  | -0.28 |  | *Yin et al., EST 2013* |
| Rice plant | Wanshan | rice seed-GX | -2.53 |  | -0.02 |  | -0.06 |  | *Yin et al., EST 2013* |
| Rice plant | Wanshan | rice seed-GX | -2.56 |  | -0.02 |  | -0.05 |  | *Yin et al., EST 2013* |
| Rice plant | Wanshan | rice seed-GX | -2.45 |  | -0.02 |  | -0.05 |  | *Yin et al., EST 2013* |
| Rice plant | Wanshan | rice root-GX | -1.75 |  | 0.00 |  | -0.03 |  | *Yin et al., EST 2013* |
| Rice plant | Wanshan | rice root-GX | -1.98 |  | 0.01 |  | -0.04 |  | *Yin et al., EST 2013* |
| Rice plant | Wanshan | rice root-GX | -1.85 |  | 0.02 |  | -0.03 |  | *Yin et al., EST 2013* |
| Rice plant | Wanshan | rice stem-GX | -2.98 |  | -0.08 |  | -0.11 |  | *Yin et al., EST 2013* |
| Rice plant | Wanshan | rice stem-GX | -3.03 |  | -0.08 |  | -0.10 |  | *Yin et al., EST 2013* |
| Rice plant | Wanshan | rice stem-GX | -2.88 |  | -0.07 |  | -0.13 |  | *Yin et al., EST 2013* |
| Rice plant | Wanshan | rice leave-GX | -3.24 |  | -0.17 |  | -0.20 |  | *Yin et al., EST 2013* |
| Rice plant | Wanshan | rice leave-GX | -3.23 |  | -0.14 |  | -0.17 |  | *Yin et al., EST 2013* |
| Rice plant | Wanshan | rice leave-GX | -3.36 |  | -0.23 |  | -0.27 |  | *Yin et al., EST 2013* |
| Rice plant | Wanshan | GOLD.-GX-1 | 0.75 |  | -0.28 |  | -0.24 |  | *Yin et al., EST 2013* |
| Rice plant | Wanshan | GOLD.-GX-2 | 0.18 |  | -0.30 |  | -0.26 |  | *Yin et al., EST 2013* |
| Rice plant | Wanshan | BB-GX-1 | -2.32 |  | -0.30 |  | -0.28 |  | *Yin et al., EST 2013* |
| Rice plant | Wanshan | BB-GX-2 | -2.16 |  | -0.34 |  | -0.32 |  | *Yin et al., EST 2013* |
| Rice plant | Wanshan | Lichen-GX-1 | -2.26 |  | -0.32 |  | -0.34 |  | *Yin et al., EST 2013* |
| Rice plant | Wanshan | Lichen-GX-2 | -2.14 |  | -0.35 |  | -0.37 |  | *Yin et al., EST 2013* |
|  |  |  |  |  |  |  |  |  |  |
| Sediment | California | 120-122.5 | -0.65 |  | 0.09 |  | 0.02 |  | *Donovan et al., 2013* |
| Sediment | California | 150-152.5 | -0.95 |  | 0.13 |  | 0.04 |  | *Donovan et al., 2013* |
| Sediment | California | 120-122.5 | -0.91 |  | 0.16 |  | 0.04 |  | *Donovan et al., 2013* |
| Sediment | California | 100-102.5 | -1.01 |  | 0.19 |  | 0.12 |  | *Donovan et al., 2013* |
| Sediment | California | 100-102.5 | -0.95 |  | 0.20 |  | 0.08 |  | *Donovan et al., 2013* |
| Sediment | California | 157.5-160 | -1.08 |  | 0.18 |  | 0.11 |  | *Donovan et al., 2013* |
| Sediment | California | 37.5-40 | -0.80 |  | 0.17 |  | 0.09 |  | *Donovan et al., 2013* |
| Sediment | California | 67.5-70 | -1.08 |  | 0.24 |  | 0.13 |  | *Donovan et al., 2013* |
| Sediment | California | 0-2.5 | -0.48 |  | 0.04 |  | 0.00 |  | *Donovan et al., 2013* |
| Sediment | California | 30-32.5 | -0.45 |  | 0.01 |  | 0.02 |  | *Donovan et al., 2013* |
| Sediment | California | 0-2.5 | -0.50 |  | 0.05 |  | 0.00 |  | *Donovan et al., 2013* |
| Sediment | California | 15-17.5 | -0.62 |  | 0.01 |  | -0.01 |  | *Donovan et al., 2013* |
| Sediment | California | 0-2.5 | -0.56 |  | 0.07 |  | 0.06 |  | *Donovan et al., 2013* |
| Sediment | California | 20-22.5 | -0.75 |  | 0.08 |  | 0.06 |  | *Donovan et al., 2013* |
| Sediment | California | 20-22.5 (rep) | -0.52 |  | 0.02 |  | -0.01 |  | *Donovan et al., 2013* |
| Sediment | California | 67.5-70 | -0.72 |  | 0.04 |  | 0.01 |  | *Donovan et al., 2013* |
| Sediment | California | 0-2.5 | -0.57 |  | 0.07 |  | 0.05 |  | *Donovan et al., 2013* |
| Sediment | California | 22.5-25 | -0.43 |  | 0.06 |  | 0.05 |  | *Donovan et al., 2013* |
| Sediment | California | 0-2.5 | -0.50 |  | 0.07 |  | 0.03 |  | *Donovan et al., 2013* |
| Sediment | California | 20-22.5 | -0.40 |  | 0.04 |  | -0.05 |  | *Donovan et al., 2013* |
| Sediment | California | 0-2.5 | -0.53 |  | 0.13 |  | 0.08 |  | *Donovan et al., 2013* |
| Sediment | California | 30-32.5 | -0.32 |  | 0.09 |  | 0.05 |  | *Donovan et al., 2013* |
| Sediment | California | 2.5-5 | -0.39 |  | 0.07 |  | 0.05 |  | *Donovan et al., 2013* |
| Sediment | California | 57.5-60 | -0.25 |  | 0.06 |  | 0.05 |  | *Donovan et al., 2013* |
| Sediment | California | 77.5-80 | 0.29 |  | -0.01 |  | 0.02 |  | *Donovan et al., 2013* |
| Sediment | California | 77.5-80 (rep) | 0.12 |  | -0.03 |  | -0.03 |  | *Donovan et al., 2013* |
| Sediment | California | 97.5-100 | -0.20 |  | 0.06 |  | 0.03 |  | *Donovan et al., 2013* |
| Sediment | California | 162.5-165 | -0.18 |  | 0.01 |  | 0.02 |  | *Donovan et al., 2013* |
| Sediment | California | 2.5-5 | -0.50 |  | 0.16 |  | 0.14 |  | *Donovan et al., 2013* |
| Sediment | California | 17.5-20 | -0.42 |  | 0.11 |  | 0.05 |  | *Donovan et al., 2013* |
| Sediment | California | 22.5-25 | -0.39 |  | 0.17 |  | 0.12 |  | *Donovan et al., 2013* |
| Sediment | California | 27.5-30 | -0.65 |  | 0.11 |  | 0.04 |  | *Donovan et al., 2013* |
| Sediment | California | a | -0.64 |  | 0.05 |  | 0.05 |  | *Donovan et al., 2013* |
| Sediment | California | b | -0.50 |  | 0.04 |  | 0.01 |  | *Donovan et al., 2013* |
| Sediment | California | AS-C[0–7.5] | -0.29 |  | 0.07 |  | 0.08 |  | *Gehrke et al., GCA, 2011* |
| Sediment | California | AS-C[109–123] | -0.06 |  | 0.03 |  | 0.03 |  | *Gehrke et al., GCA, 2011* |
| Sediment | California | AS-C[183–196] | 0.29 |  | 0.03 |  | 0.01 |  | *Gehrke et al., GCA, 2011* |
| Sediment | California | AS-M[0–17] | -0.32 |  | 0.05 |  | 0.01 |  | *Gehrke et al., GCA, 2011* |
| Sediment | California | AS-M[100–131] | 0.20 |  | 0.00 |  | -0.03 |  | *Gehrke et al., GCA, 2011* |
| Sediment | California | AS-M[174–199] | -0.15 |  | 0.03 |  | 0.02 |  | *Gehrke et al., GCA, 2011* |
| Sediment | California | CR(Ra) | -0.88 |  | 0.06 |  | -0.01 |  | *Gehrke et al., GCA, 2011* |
| Sediment | California | CR(Rb) | -0.91 |  | 0.09 |  | 0.00 |  | *Gehrke et al., GCA, 2011* |
| Sediment | California | CR(Ma) | -0.78 |  | 0.08 |  | 0.00 |  | *Gehrke et al., GCA, 2011* |
| Sediment | California | CR(Ma) (rep) | -0.73 |  | 0.07 |  | -0.04 |  | *Gehrke et al., GCA, 2011* |
| Sediment | California | CR(Mb) | -0.75 |  | 0.04 |  | 0.02 |  | *Gehrke et al., GCA, 2011* |
| Sediment | California | YB(a) | -0.72 |  | 0.12 |  | 0.03 |  | *Gehrke et al., GCA, 2011* |
| Sediment | California | YB(c) | -0.65 |  | 0.08 |  | 0.02 |  | *Gehrke et al., GCA, 2011* |
| Sediment | California | YB(b) | -0.73 |  | 0.16 |  | 0.10 |  | *Gehrke et al., GCA, 2011* |
| Sediment | California | IT(-7) | -0.31 |  | 0.11 |  | 0.06 |  | *Gehrke et al., GCA, 2011* |
| Sediment | California | IT(-7) (rep) | -0.30 |  | 0.03 |  | 0.05 |  | *Gehrke et al., GCA, 2011* |
| Sediment | California | IT(-5b) | -0.43 |  | 0.09 |  | 0.04 |  | *Gehrke et al., GCA, 2011* |
| Sediment | California | IT(-5a) | -0.32 |  | 0.07 |  | 0.02 |  | *Gehrke et al., GCA, 2011* |
| Sediment | California | IT(-2) | -0.45 |  | 0.04 |  | 0.01 |  | *Gehrke et al., GCA, 2011* |
| Sediment | California | IT(-2) (rep) | -0.35 |  | 0.05 |  | 0.03 |  | *Gehrke et al., GCA, 2011* |
| Sediment | California | IT(-1) | -0.46 |  | 0.12 |  | 0.08 |  | *Gehrke et al., GCA, 2011* |
| Sediment | California | IT(14) | -0.53 |  | 0.09 |  | 0.08 |  | *Gehrke et al., GCA, 2011* |
| Sediment | California | IT(21) | -0.54 |  | 0.04 |  | 0.04 |  | *Gehrke et al., GCA, 2011* |
| Sediment | California | IT(42b) | -0.59 |  | 0.14 |  | 0.10 |  | *Gehrke et al., GCA, 2011* |
| Sediment | California | IT(42a) | -0.53 |  | 0.06 |  | 0.04 |  | *Gehrke et al., GCA, 2011* |
| Sediment | California | IT(52) | -0.65 |  | 0.10 |  | 0.07 |  | *Gehrke et al., GCA, 2011* |
| Sediment | California | IT(62a) | -0.65 |  | 0.12 |  | 0.11 |  | *Gehrke et al., GCA, 2011* |
| Sediment | California | IT(62b) | -0.69 |  | 0.13 |  | 0.03 |  | *Gehrke et al., GCA, 2011* |
| Sediment | California | IT(91) | -0.73 |  | 0.05 |  | 0.02 |  | *Gehrke et al., GCA, 2011* |
| Sediment | California | IT(90) | -0.59 |  | 0.06 |  | 0.06 |  | *Gehrke et al., GCA, 2011* |
| Sediment | California | IT(90) (rep) | -0.71 |  | 0.09 |  | 0.06 |  | *Gehrke et al., GCA, 2011* |
| Sediment | California | IT(85) | -0.62 |  | 0.08 |  | 0.04 |  | *Gehrke et al., GCA, 2011* |
| Sediment | California | IT(87) | -0.59 |  | 0.07 |  | 0.05 |  | *Gehrke et al., GCA, 2011* |
| Sediment | California | IT(110) | -0.90 |  | 0.10 |  | 0.03 |  | *Gehrke et al., GCA, 2011* |
| Sediment | California | IT(115) | -0.74 |  | 0.11 |  | 0.06 |  | *Gehrke et al., GCA, 2011* |
| Sediment | California | IT(119) | -0.99 |  | 0.07 |  | -0.01 |  | *Gehrke et al., GCA, 2011* |
| Sediment | California | IT(143) | -0.85 |  | 0.09 |  | 0.02 |  | *Gehrke et al., GCA, 2011* |
| Sediment | California | WL(-3) | -0.44 |  | 0.08 |  | 0.06 |  | *Gehrke et al., GCA, 2011* |
| Sediment | California | WL(9c) | -0.50 |  | 0.12 |  | 0.07 |  | *Gehrke et al., GCA, 2011* |
| Sediment | California | WL(9a) | -0.61 |  | 0.11 |  | 0.06 |  | *Gehrke et al., GCA, 2011* |
| Sediment | California | WL(9b) | -0.56 |  | 0.08 |  | 0.03 |  | *Gehrke et al., GCA, 2011* |
| Sediment | California | WL(16) | -0.60 |  | 0.16 |  | 0.12 |  | *Gehrke et al., GCA, 2011* |
| Sediment | California | WL(18) | -0.68 |  | 0.14 |  | 0.10 |  | *Gehrke et al., GCA, 2011* |
| Sediment | California | WL(21) | -0.66 |  | 0.16 |  | 0.11 |  | *Gehrke et al., GCA, 2011* |
| Sediment | California | WL(22) | -0.57 |  | 0.14 |  | 0.04 |  | *Gehrke et al., GCA, 2011* |
| Sediment | California | WL(24) | -0.67 |  | 0.14 |  | 0.05 |  | *Gehrke et al., GCA, 2011* |
|  |  |  |  |  |  |  |  |  |  |
| Sediment | Idrijca River | Travnik | -0.45 |  | -0.06 |  | -0.05 |  | *Foucher et al., 2009* |
| Sediment | Soča/Isonzo River | Soča-Y12 | -0.13 |  | -0.05 |  | -0.06 |  | *Foucher et al., 2009* |
| Sediment | Soča/Isonzo River | Soča-Y1 | -0.36 |  | -0.05 |  | -0.02 |  | *Foucher et al., 2009* |
| Sediment | Cajarc core | CI 4 | 0.11 |  | -0.09 |  | -0.12 |  | *Sonke et al., 2010* |
| Sediment | Cajarc core | CI 8 | 0.11 |  | -0.09 |  | -0.12 |  | *Sonke et al., 2010* |
| Sediment | Cajarc core | CI 10 | -0.21 |  | -0.05 |  | -0.10 |  | *Sonke et al., 2010* |
| Sediment | Cajarc core | CI 16 | 0.38 |  | -0.12 |  | -0.08 |  | *Sonke et al., 2010* |
| Sediment | Cajarc core | CI 17 | 0.28 |  | -0.07 |  | -0.08 |  | *Sonke et al., 2010* |
| Sediment | Cajarc core | CI 20 | 0.21 |  | -0.08 |  | -0.07 |  | *Sonke et al., 2010* |
| Sediment | Cajarc core | CI 21 | 0.51 |  | -0.10 |  | -0.07 |  | *Sonke et al., 2010* |
| Sediment | Cajarc core | C 16 | 0.15 |  | 0.07 |  | -0.08 |  | *Sonke et al., 2010* |
| Sediment | Cajarc core | C 17 | 0.28 |  | -0.08 |  | -0.06 |  | *Sonke et al., 2010* |
| Sediment | Cajarc core | C 18 | 0.15 |  | -0.06 |  | -0.04 |  | *Sonke et al., 2010* |
| Sediment | Cajarc core | C 20 | -0.01 |  | -0.08 |  | -0.07 |  | *Sonke et al., 2010* |
| Sediment | Cajarc core | C 22 | 0.48 |  | -0.09 |  | -0.07 |  | *Sonke et al., 2010* |
| Sediment | Cajarc core | C 27 | -0.15 |  | -0.07 |  | -0.13 |  | *Sonke et al., 2010* |
| Sediment | Cajarc core | D22 | -0.03 |  | -0.10 |  | -0.12 |  | *Sonke et al., 2010* |
| Sediment | Cajarc core | D23 | 0.64 |  | -0.15 |  | -0.11 |  | *Sonke et al., 2010* |
| Sediment | Cajarc core | D25 | 0.08 |  | -0.06 |  | -0.07 |  | *Sonke et al., 2010* |
| Sediment | Cajarc core | D27 | 0.71 |  | -0.14 |  | -0.11 |  | *Sonke et al., 2010* |
| Sediment | Marcenac core | MI 1 | -1.04 |  | -0.35 |  | -0.34 |  | *Sonke et al., 2010* |
| Sediment | Marcenac core | MI 6 | -1.22 |  | -0.07 |  | -0.12 |  | *Sonke et al., 2010* |
| Sediment | Marcenac core | MI 10 | -0.75 |  | -0.19 |  | -0.25 |  | *Sonke et al., 2010* |
| Sediment | Marcenac core | MI 15 | -0.86 |  | -0.28 |  | -0.26 |  | *Sonke et al., 2010* |
| Sediment | Marcenac core | MI 40 | -1.11 |  | -0.12 |  | -0.19 |  | *Sonke et al., 2010* |
| Sediment | Kempen sediments | M3-31 | -1.44 |  | 0.03 |  | -0.01 |  | *Sonke et al., 2010* |
| Sediment | Kempen sediments | M3-36 | -1.21 |  | -0.03 |  | 0.03 |  | *Sonke et al., 2010* |
| Sediment | Kempen sediments | M3-40 | -1.28 |  | 0.06 |  | -0.03 |  | *Sonke et al., 2010* |
| Sediment | Kempen sediments | b1 | 0.02 |  | -0.10 |  | -0.15 |  | *Sonke et al., 2010* |
| Sediment | Kempen sediments | b3 | -0.32 |  | -0.11 |  | -0.12 |  | *Sonke et al., 2010* |
| Sediment | Kempen sediments | b5 | -0.31 |  | -0.08 |  | -0.13 |  | *Sonke et al., 2010* |
| Sediment | Kempen sediments | b7 | -0.10 |  | -0.10 |  | -0.10 |  | *Sonke et al., 2010* |
| Sediment | Kempen sediments | b9 | -0.15 |  | -0.09 |  | -0.11 |  | *Sonke et al., 2010* |
| Sediment | Kempen sediments | b12 | -0.23 |  | -0.08 |  | -0.11 |  | *Sonke et al., 2010* |
| Sediment | Kempen sediments | b15 | 0.12 |  | -0.09 |  | -0.14 |  | *Sonke et al., 2010* |
| Sediment | Kempen sediments | b16 | 0.05 |  | -0.12 |  | -0.18 |  | *Sonke et al., 2010* |
| Sediment | Kempen sediments | b19 | 0.07 |  | -0.10 |  | -0.15 |  | *Sonke et al., 2010* |
| Sediment | Kempen sediments | b22 | 0.11 |  | -0.13 |  | -0.16 |  | *Sonke et al., 2010* |
| Sediments | dongjiang River | A1 | -0.80 |  | -0.02 |  | -0.03 |  | *Liu et al., 2011* |
| Sediments | dongjiang River | A3 | -1.14 |  | -0.02 |  | -0.03 |  | *Liu et al., 2011* |
| Sediments | dongjiang River | A4 | -1.00 |  | -0.02 |  | 0.00 |  | *Liu et al., 2011* |
| Sediments | dongjiang River | A5 | -0.91 |  | -0.02 |  | -0.03 |  | *Liu et al., 2011* |
| Sediments | dongjiang River | A7 | -0.67 |  | -0.01 |  | -0.03 |  | *Liu et al., 2011* |
| Sediments | dongjiang River | B2 | -1.12 |  | -0.04 |  | -0.03 |  | *Liu et al., 2011* |
| Sediments | dongjiang River | B3 | -1.14 |  | -0.02 |  | -0.01 |  | *Liu et al., 2011* |
| Sediments | dongjiang River | C1 | -0.71 |  | -0.03 |  | -0.03 |  | *Liu et al., 2011* |
| Sediments | dongjiang River | C7 | -0.60 |  | -0.02 |  | -0.05 |  | *Liu et al., 2011* |
| Sediments | dongjiang River | C8 | -0.72 |  | -0.02 |  | -0.02 |  | *Liu et al., 2011* |
| Sediments | dongjiang River | D6 | -1.00 |  | -0.03 |  | 0.03 |  | *Liu et al., 2011* |
| Sediments | dongjiang River | D8 | -0.90 |  | -0.04 |  | -0.02 |  | *Liu et al., 2011* |
| Sediments | dongjiang River | Z1 | -1.55 |  | -0.24 |  | -0.18 |  | *Liu et al., 2011* |
| Sediments | dongjiang River | Z4 | -1.99 |  | -0.20 |  | -0.16 |  | *Liu et al., 2011* |
| Sediments | dongjiang River | Z7 | -2.06 |  | -0.21 |  | -0.19 |  | *Liu et al., 2011* |
| Sediments | dongjiang River | Z14 | -2.16 |  | -0.27 |  | -0.21 |  | *Liu et al., 2011* |
| Sediments | dongjiang River | E2 | -2.35 |  | -0.08 |  | -0.07 |  | *Liu et al., 2011* |
| Sediments | dongjiang River | E9 | -1.96 |  | -0.10 |  | -0.10 |  | *Liu et al., 2011* |
| Sediments | dongjiang River | E11 | -1.88 |  | -0.09 |  | -0.09 |  | *Liu et al., 2011* |
| Sediments | dongjiang River | E12 | -2.31 |  | -0.08 |  | -0.07 |  | *Liu et al., 2011* |
| Sediments | dongjiang River | BCR 580 | -0.46 |  | -0.02 |  | -0.03 |  | *Liu et al., 2011* |
| Sediment | Baihua Lake | 1 | -1.10 |  | 0.01 |  | -0.04 |  | *Feng et al., 2010* |
| Sediment | Baihua Lake | 2 | -0.81 |  | -0.02 |  | -0.05 |  | *Feng et al., 2010* |
| Sediment | Baihua Lake | 4 | -0.85 |  | 0.00 |  | -0.02 |  | *Feng et al., 2010* |
| Sediment | Baihua Lake | 6 | -0.87 |  | -0.01 |  | -0.04 |  | *Feng et al., 2010* |
| Sediment | Baihua Lake | 10 | -0.70 |  | -0.03 |  | -0.05 |  | *Feng et al., 2010* |
| Sediment | Baihua Lake | 10 | -0.62 |  | -0.01 |  | -0.04 |  | *Feng et al., 2010* |
| Sediment | Baihua Lake | 12 | -0.75 |  | -0.03 |  | -0.06 |  | *Feng et al., 2010* |
| Sediment | Baihua Lake | 14 | -0.77 |  | -0.03 |  | -0.05 |  | *Feng et al., 2010* |
| Sediment | Baihua Lake | 16 | -0.60 |  | -0.03 |  | -0.05 |  | *Feng et al., 2010* |
| Sediment | Baihua Lake | 18 | -0.69 |  | -0.02 |  | -0.05 |  | *Feng et al., 2010* |
| Sediment | Baihua Lake | 20 | -0.75 |  | -0.04 |  | -0.06 |  | *Feng et al., 2010* |
| Sediment | Baihua Lake | 22 | -0.75 |  | -0.07 |  | -0.06 |  | *Feng et al., 2010* |
| Sediment | Baihua Lake | 24 | -0.87 |  | -0.01 |  | -0.05 |  | *Feng et al., 2010* |
| Sediment | Baihua Lake | 26 | -0.93 |  | -0.04 |  | -0.06 |  | *Feng et al., 2010* |
| Sediment | Baihua Lake | 28 | -1.10 |  | -0.08 |  | -0.08 |  | *Feng et al., 2010* |
| Sediment | Hongfeng lake | 1 | -2.02 |  | -0.02 |  | -0.06 |  | *Feng et al., 2010* |
| Sediment | Hongfeng lake | 3 | -1.79 |  | -0.06 |  | -0.08 |  | *Feng et al., 2010* |
| Sediment | Hongfeng lake | 4 | -1.77 |  | -0.11 |  | -0.13 |  | *Feng et al., 2010* |
| Sediment | Hongfeng lake | 5 | -1.85 |  | -0.04 |  | -0.06 |  | *Feng et al., 2010* |
| Sediment | Hongfeng lake | 6 | -1.73 |  | -0.08 |  | -0.10 |  | *Feng et al., 2010* |
| Sediment | Hongfeng lake | 9 | -1.76 |  | -0.03 |  | -0.08 |  | *Feng et al., 2010* |
| Sediment | Hongfeng lake | 11 | -1.93 |  | 0.00 |  | -0.06 |  | *Feng et al., 2010* |
| Sediment | Hongfeng lake | 14 | -1.87 |  | -0.02 |  | -0.08 |  | *Feng et al., 2010* |
| Sediment | Hongfeng lake | 16 | -1.95 |  | -0.01 |  | -0.06 |  | *Feng et al., 2010* |
| Sediment | Hongfeng lake | 18 | -1.84 |  | -0.06 |  | -0.09 |  | *Feng et al., 2010* |
| Sediment | Hongfeng lake | 20 | -1.67 |  | -0.08 |  | -0.11 |  | *Feng et al., 2010* |
| Sediment | Hongfeng lake | 22 | -1.70 |  | -0.12 |  | -0.14 |  | *Feng et al., 2010* |
| Sediment | Hongfeng lake | 24 | -1.83 |  | -0.08 |  | -0.13 |  | *Feng et al., 2010* |
| Sediment | Hongfeng lake | 26 | -1.68 |  | -0.09 |  | -0.13 |  | *Feng et al., 2010* |
| Sediment | Hongfeng lake | 28 | -1.80 |  | -0.16 |  | -0.16 |  | *Feng et al., 2010* |
| Sediment | Hongfeng lake | 30 | -1.76 |  | -0.14 |  | -0.14 |  | *Feng et al., 2010* |
| Sediment | Hongfeng lake | 32 | -1.75 |  | -0.10 |  | -0.17 |  | *Feng et al., 2010* |
| Sediment | Hongfeng lake | 34 | -1.87 |  | -0.08 |  | -0.13 |  | *Feng et al., 2010* |
| Sediment | Hongfeng lake | 39 | -1.75 |  | -0.15 |  | -0.17 |  | *Feng et al., 2010* |
| Sediment | Wanshan mercury mine | S1 | 0.12 |  | 0.02 |  | 0.03 |  | *Yin et al., 2013 Chem. Geol.* |
| Sediment | Wanshan mercury mine | S2 | 0.21 |  | -0.05 |  | -0.03 |  | *Yin et al., 2013 Chem. Geol.* |
| Sediment | Wanshan mercury mine | S3 | 0.05 |  | 0.03 |  | -0.02 |  | *Yin et al., 2013 Chem. Geol.* |
| Sediment | Wanshan mercury mine | S4 | 0.11 |  | 0.02 |  | 0.02 |  | *Yin et al., 2013 Chem. Geol.* |
| Sediment | Wanshan mercury mine | S5 | 0.04 |  | -0.02 |  | 0.02 |  | *Yin et al., 2013 Chem. Geol.* |
| Sediment | Wanshan mercury mine | S6 | -0.09 |  | 0.02 |  | 0.02 |  | *Yin et al., 2013 Chem. Geol.* |
| Sediment | Wanshan mercury mine | S7 | -0.15 |  | -0.01 |  | -0.02 |  | *Yin et al., 2013 Chem. Geol.* |
| Sediment | Wanshan mercury mine | S8 | -0.34 |  | 0.01 |  | -0.01 |  | *Yin et al., 2013 Chem. Geol.* |
| Sediment | Wanshan mercury mine | S9 | -0.45 |  | 0.04 |  | 0.02 |  | *Yin et al., 2013 Chem. Geol.* |
| Sediment | Wanshan mercury mine | S10 | -0.65 |  | 0.03 |  | 0.04 |  | *Yin et al., 2013 Chem. Geol.* |
| Sediment | Wanshan mercury mine | S11 | -0.57 |  | 0.00 |  | -0.03 |  | *Yin et al., 2013 Chem. Geol.* |
| Sediment | Wanshan mercury mine | S12 | -0.53 |  | 0.02 |  | 0.01 |  | *Yin et al., 2013 Chem. Geol.* |
| Sediment | Wanshan mercury mine | S13 | -0.83 |  | 0.03 |  | -0.03 |  | *Yin et al., 2013 Chem. Geol.* |
| Sediment | Wanshan mercury mine | S14 | -0.79 |  | 0.00 |  | -0.01 |  | *Yin et al., 2013 Chem. Geol.* |
| Sediment | Canada | Lake D | -0.44 |  | 0.20 |  | 0.12 |  | *Gantner et al., 2009 EST* |
| Sediment | Canada | Lake G | -0.82 |  | 0.06 |  | 0.05 |  | *Gantner et al., 2009 EST* |
| Sediment | Canada | Lake G | -0.84 |  | -0.03 |  | 0.02 |  | *Gantner et al., 2009 EST* |
| Sediment | Canada | Hazen 2007-1a | -1.06 |  | -0.44 |  | -0.42 |  | *Gantner et al., 2009 EST* |
| Sediment | Canada | Hazen 2007-1b | -1.11 |  | -0.34 |  | -0.32 |  | *Gantner et al., 2009 EST* |
| Sediment | Canada | Hazen 2007-1c | -1.16 |  | -0.34 |  | -0.29 |  | *Gantner et al., 2009 EST* |
| Sediment | Canada | Hazen 2007-2 | -1.13 |  | -0.16 |  | -0.25 |  | *Gantner et al., 2009 EST* |
| Sediment | Canada | Hazen 2007-3 | -1.13 |  | -0.14 |  | -0.26 |  | *Gantner et al., 2009 EST* |
| Sediment | Canada | Hazen 2006 | -1.01 |  | 0.04 |  | -0.02 |  | *Gantner et al., 2009 EST* |
| Sediment | Canada | Amituk L | -0.96 |  | -0.09 |  | -0.16 |  | *Gantner et al., 2009 EST* |
| Sediment | Canada | 9Mile L | -0.71 |  | 0.36 |  | 0.25 |  | *Gantner et al., 2009 EST* |
| Sediment | Canada | Resolute L | -2.03 |  | 0.16 |  | 0.08 |  | *Gantner et al., 2009 EST* |
| Sediment | Canada | Gavia FL-1 | -0.57 |  | 0.64 |  | 0.36 |  | *Gantner et al., 2009 EST* |
| Sediment | Canada | Gavia FL-2 | -0.75 |  | 0.62 |  | 0.42 |  | *Gantner et al., 2009 EST* |
| Sediment | Canada | Gavia FL-3 | -0.74 |  | 0.49 |  | 0.37 |  | *Gantner et al., 2009 EST* |
| Sediment | Canada | Little N L | -0.63 |  | 0.06 |  | 0.05 |  | *Gantner et al., 2009 EST* |
| Sediment | Canada | Little N L | -0.86 |  | 0.08 |  | 0.04 |  | *Gantner et al., 2009 EST* |
| Sediment | Canada | Notgordie L | -1.64 |  | 0.74 |  | 0.26 |  | *Gantner et al., 2009 EST* |
| Sediment | Canada | Pingualuk | 0.25 |  | -0.26 |  | -0.52 |  | *Gantner et al., 2009 EST* |
| Sediment | Lake Baikal | BIL-1 | -1.99 |  | -0.23 |  | -0.07 |  | *Perrot et al., 2011 EST* |
| Sediment | Lake Baikal | B371-A1 | -0.59 |  | -0.06 |  | -0.14 |  | *Perrot et al., 2011 EST* |
| Sediment | Lake Baikal | B371-A2 | -0.51 |  | 0.07 |  | -0.02 |  | *Perrot et al., 2011 EST* |
| Sediment | Lake Baikal | B371-A3 | -0.47 |  | -0.05 |  | 0.03 |  | *Perrot et al., 2011 EST* |
| Sediment | Lake Baikal | B372-A1 | -0.72 |  | -0.06 |  | -0.13 |  | *Perrot et al., 2011 EST* |
| Sediment | Lake Baikal | B372-A2 | -0.71 |  | -0.05 |  | -0.09 |  | *Perrot et al., 2011 EST* |
| Sediment | Lake Baikal | B376-B1 | -0.43 |  | 0.02 |  | 0.06 |  | *Perrot et al., 2011 EST* |
| Sediment | Lake Baikal | B376-B2 | -0.92 |  | -0.02 |  | 0.04 |  | *Perrot et al., 2011 EST* |
| Sediment | Lake Baikal | B376-B3 | -0.86 |  | -0.01 |  | -0.10 |  | *Perrot et al., 2011 EST* |
| Sediment | Lake Baikal | B376-B4 | -0.56 |  | 0.01 |  | -0.03 |  | *Perrot et al., 2011 EST* |
| Soil | New Hampshire | NH-1 | -1.83 |  | -0.40 |  | -0.38 |  | *Biswas et al., 2008 EST* |
| Soil | New Hampshire | NH-1 | -1.87 |  | -0.32 |  | -0.32 |  | *Biswas et al., 2008 EST* |
| Soil | Alaska | AK-2 | -1.29 |  | -0.15 |  | -0.12 |  | *Biswas et al., 2008 EST* |
| Soil | Alaska | AK-2 | -1.32 |  | -0.14 |  | -0.13 |  | *Biswas et al., 2008 EST* |
| Soil | Alaska | AK-3 | -1.21 |  | -0.21 |  | -0.22 |  | *Biswas et al., 2008 EST* |
| Soil | Alaska | AK-3 | -1.10 |  | -0.24 |  | -0.22 |  | *Biswas et al., 2008 EST* |
| Soil | Alaska | AK-3 | 1.19 |  | -0.22 |  | -0.18 |  | *Biswas et al., 2008 EST* |
| Soil | Belize | BE-1 | -1.42 |  | -0.20 |  | -0.21 |  | *Biswas et al., 2008 EST* |
| Soil | Washington | WA-2 | -1.48 |  | -0.19 |  | -0.15 |  | *Biswas et al., 2008 EST* |
| Soil | Wyoming | WY-1 | -0.96 |  | 0.03 |  | 0.09 |  | *Biswas et al., 2008 EST* |
| Soil | Northeastern France | SJ02* | -0.94 |  | 0.07 |  | -0.01 |  | *Estrade et al., 2010 EST* |
| Soil | Northeastern France | SJ38* | 0.73 |  | -0.05 |  | -0.04 |  | *Estrade et al., 2010 EST* |
| Soil | Northeastern France | SJ47* | -0.78 |  | -0.12 |  | -0.07 |  | *Estrade et al., 2010 EST* |
| Soil | Northeastern France | SJ14 | -0.72 |  | -0.03 |  | -0.05 |  | *Estrade et al., 2010 EST* |
| Soil | Northeastern France | SJ27 | -0.60 |  | -0.07 |  | -0.05 |  | *Estrade et al., 2010 EST* |
| Soil | Northeastern France | SJ12 | -0.71 |  | -0.04 |  | -0.06 |  | *Estrade et al., 2010 EST* |
| Soil | Northeastern France | SJ01 | -0.72 |  | -0.08 |  | -0.08 |  | *Estrade et al., 2010 EST* |
| Soil | Northeastern France | MC05 | -0.66 |  | -0.06 |  | -0.06 |  | *Estrade et al., 2010 EST* |
| Soil | Northeastern France | SJ30 | -0.43 |  | -0.05 |  | -0.05 |  | *Estrade et al., 2010 EST* |
| Soil | Northeastern France | MC10 | -0.45 |  | -0.08 |  | 0.00 |  | *Estrade et al., 2010 EST* |
| Soil | Northeastern France | MB05 | -0.43 |  | 0.03 |  | -0.09 |  | *Estrade et al., 2010 EST* |
| Soil | Northeastern France | SJ40 | -0.20 |  | 0.09 |  | 0.06 |  | *Estrade et al., 2010 EST* |
| Soil | Northeastern France | SJ52 | -0.31 |  | -0.04 |  | -0.03 |  | *Estrade et al., 2010 EST* |
| Soil | Northeastern France | SJ21 | -0.16 |  | -0.05 |  | -0.07 |  | *Estrade et al., 2010 EST* |
| Soil | Wanshan mercury mine | S1 | -0.19 |  | 0.02 |  | 0.04 |  | *Yin et al., 2013b Chem Geol* |
| Soil | Wanshan mercury mine | S2 | -0.29 |  | 0.02 |  | 0.05 |  | *Yin et al., 2013b Chem Geol* |
| Soil | Wanshan mercury mine | S3 | -0.05 |  | 0.05 |  | 0.05 |  | *Yin et al., 2013b Chem Geol* |
| Soil | Wanshan mercury mine | S4 | 0.03 |  | 0.05 |  | 0.03 |  | *Yin et al., 2013b Chem Geol* |
| Soil | Wanshan mercury mine | S5 | -0.03 |  | 0.04 |  | -0.07 |  | *Yin et al., 2013b Chem Geol* |
| Soil | Wanshan mercury mine | S6 | 0.14 |  | 0.07 |  | 0.02 |  | *Yin et al., 2013b Chem Geol* |
| Soil | Wanshan mercury mine | S7 | 0.15 |  | -0.04 |  | 0.02 |  | *Yin et al., 2013b Chem Geol* |
| Soil | Wanshan mercury mine | S8 | 0.09 |  | -0.05 |  | 0.00 |  | *Yin et al., 2013b Chem Geol* |
| Soil | Northern France | 16B | -0.90 |  | -0.04 |  | -0.02 |  | *Estrade et al., 2010 EST* |
| Soil | Northern France | 17B | -0.70 |  | -0.03 |  | -0.04 |  | *Estrade et al., 2010 EST* |
| Soil | Northern France | 22B | -0.78 |  | -0.02 |  | -0.05 |  | *Estrade et al., 2010 EST* |
| Soil | Northern France | 1B | -0.77 |  | -0.04 |  | -0.09 |  | *Estrade et al., 2010 EST* |
| Soil | Northern France | 52B | -0.62 |  | 0.04 |  | 0.00 |  | *Estrade et al., 2010 EST* |
| Soil | Northern France | 55B | -0.59 |  | 0.02 |  | 0.02 |  | *Estrade et al., 2010 EST* |
| Soil | Northern France | 36B | -0.61 |  | 0.02 |  | 0.03 |  | *Estrade et al., 2010 EST* |
| Soil | Northern France | 32B | -0.59 |  | 0.04 |  | 0.00 |  | *Estrade et al., 2010 EST* |
| Soil | Northern France | 31B | -0.64 |  | 0.05 |  | -0.02 |  | *Estrade et al., 2010 EST* |
| Soil | Northern France | 34B | -0.56 |  | 0.05 |  | 0.05 |  | *Estrade et al., 2010 EST* |
| Soil | Northern France | 30B | -0.50 |  | 0.02 |  | 0.07 |  | *Estrade et al., 2010 EST* |
| Soil | 2185 | LS-1 | -2.27 |  | -0.48 |  | -0.43 |  | *Zhang et al., 2013 SR* |
| Soil | 2151 | LS-2 | -2.63 |  | -0.32 |  | -0.33 |  | *Zhang et al., 2013 SR* |
| Soil | 2015 | LS-3 | -2.36 |  | -0.27 |  | -0.26 |  | *Zhang et al., 2013 SR* |
| Soil | 1836 | LS-4 | -2.13 |  | -0.28 |  | -0.29 |  | *Zhang et al., 2013 SR* |
| Soil | 1804 | LS-5 | -1.94 |  | -0.37 |  | -0.34 |  | *Zhang et al., 2013 SR* |
| Soil | 1714 | LS-6 | -2.43 |  | -0.30 |  | -0.30 |  | *Zhang et al., 2013 SR* |
| Soil | 1675 | LS-7 | -2.18 |  | -0.22 |  | -0.22 |  | *Zhang et al., 2013 SR* |
| Soil | 1617 | LS-8 | -2.28 |  | -0.35 |  | -0.36 |  | *Zhang et al., 2013 SR* |
| Soil | 1542 | LS-9 | -2.09 |  | -0.28 |  | -0.29 |  | *Zhang et al., 2013 SR* |
| Soil | 1496 | LS-10 | -2.13 |  | -0.29 |  | -0.29 |  | *Zhang et al., 2013 SR* |
| Soil | 1413 | LS-11 | -1.97 |  | -0.24 |  | -0.25 |  | *Zhang et al., 2013 SR* |
| Soil | 1351 | LS-12 | -1.98 |  | -0.34 |  | -0.34 |  | *Zhang et al., 2013 SR* |
| Soil | 1308 | LS-13 | -1.85 |  | -0.17 |  | -0.16 |  | *Zhang et al., 2013 SR* |
| Soil | 1214 | LS-14 | -1.73 |  | -0.12 |  | -0.12 |  | *Zhang et al., 2013 SR* |
| Soil | 1121 | LS-15 | -1.52 |  | -0.08 |  | -0.07 |  | *Zhang et al., 2013 SR* |
| Soil | 1078 | LS-16 | -1.57 |  | -0.13 |  | -0.11 |  | *Zhang et al., 2013 SR* |
| Soil | 983 | LS-17 | -1.74 |  | -0.06 |  | -0.04 |  | *Zhang et al., 2013 SR* |
| Soil | 957 | LS-18 | -1.61 |  | -0.13 |  | -0.12 |  | *Zhang et al., 2013 SR* |
| Soil | 908 | LS-19 | -1.52 |  | -0.11 |  | -0.13 |  | *Zhang et al., 2013 SR* |
| Soil | 867 | LS-20 | -1.42 |  | -0.06 |  | -0.06 |  | *Zhang et al., 2013 SR* |
| Soil | 806 | LS-21 | -1.62 |  | -0.12 |  | -0.13 |  | *Zhang et al., 2013 SR* |
| Soil | Wanshan mercury mine | Soil-WK | -0.34 |  | -0.02 |  | -0.01 |  | *Yin et al., EST 2013* |
| Soil | Wanshan mercury mine | Soil-WK | -0.39 |  | 0.02 |  | 0.02 |  | *Yin et al., EST 2013* |
| Soil | Wanshan mercury mine | Soil-WK | -0.28 |  | 0.03 |  | 0.02 |  | *Yin et al., EST 2013* |
| Soil | Wanshan mercury mine | Soil-WK | -0.35 |  | -0.02 |  | -0.01 |  | *Yin et al., EST 2013* |
| Soil | Wanshan mercury mine | Soil-GX | -0.48 |  | 0.06 |  | 0.01 |  | *Yin et al., EST 2013* |
| Soil | Wanshan mercury mine | Soil-GX | -0.65 |  | 0.07 |  | -0.02 |  | *Yin et al., EST 2013* |
| Soil | Wanshan mercury mine | Soil-GX | -0.52 |  | 0.06 |  | 0.03 |  | *Yin et al., EST 2013* |
| Soil | Wanshan mercury mine | Soil-GX | -0.45 |  | 0.04 |  | 0.02 |  | *Yin et al., EST 2013* |
| Soil | California | Upland fores | -1.54 |  | -0.27 |  | -0.23 |  | *Tsui et al., ES&T, 2012* |
| Sediment | Mediterranean Sea | 974C 6H5 38-39 | -0.79 |  | 0.08 |  | 0.05 |  | *Gehrke et al., GCA, 2009* |
| Sediment | Mediterranean Sea | 974C 6H5 38-39 (rep) | -0.61 |  | 0.10 |  | 0.05 |  | *Gehrke et al., GCA, 2009* |
| Sediment | Mediterranean Sea | 974C 6H5 39-40 | -0.99 |  | 0.17 |  | 0.10 |  | *Gehrke et al., GCA, 2009* |
| Sediment | Mediterranean Sea | 974C 6H5 40-41 | -1.11 |  | 0.11 |  | 0.02 |  | *Gehrke et al., GCA, 2009* |
| Sediment | Mediterranean Sea | 974C 6H5 62-63 | -0.86 |  | 0.09 |  | 0.01 |  | *Gehrke et al., GCA, 2009* |
| Sediment | Mediterranean Sea | 974C 6H5 63-64 | -0.91 |  | 0.10 |  | 0.03 |  | *Gehrke et al., GCA, 2009* |
| Sediment | Mediterranean Sea | 974C 6H5 50-51 | -0.74 |  | 0.07 |  | 0.01 |  | *Gehrke et al., GCA, 2009* |
| Sediment | Mediterranean Sea | 974C 6H5 69-70 | -0.57 |  | 0.07 |  | -0.04 |  | *Gehrke et al., GCA, 2009* |
| Sediment | Mediterranean Sea | 974C 6H5 75-77 | -0.86 |  | 0.05 |  | -0.04 |  | *Gehrke et al., GCA, 2009* |
| Sediment | Mediterranean Sea | 974C 6H5 80-82 | -0.97 |  | 0.04 |  | 0.00 |  | *Gehrke et al., GCA, 2009* |
| Sediment | Mediterranean Sea | 974C 6H5 85-87 | -0.67 |  | 0.04 |  | -0.02 |  | *Gehrke et al., GCA, 2009* |
| Sediment | Soča/Isonzo Estuary | Soča-Stn6(0.5cm) | -0.30 |  | -0.06 |  | -0.05 |  | *Foucher et al., ES&T, 2009* |
| Sediment | Soča/Isonzo Estuary | Soča-Stn6(7cm) | -0.14 |  | -0.05 |  | -0.06 |  | *Foucher et al., ES&T, 2009* |
| Sediment | Soča/Isonzo Estuary | Soča-Stn6(14cm) | -0.27 |  | -0.06 |  | -0.02 |  | *Foucher et al., ES&T, 2009* |
| Sediment | Soča/Isonzo Estuary | Soča-Stn6(2cm) | -0.14 |  | -0.06 |  | -0.04 |  | *Foucher et al., ES&T, 2009* |
| Sediment | Soča/Isonzo Estuary | Soča-Stn6(8cm) | -0.22 |  | -0.02 |  | 0.00 |  | *Foucher et al., ES&T, 2009* |
| Sediment | Soča/Isonzo Estuary | Soča-Stn6(15cm) | -0.26 |  | -0.04 |  | -0.01 |  | *Foucher et al., ES&T, 2009* |
| Sediment | Gulf of Trieste | GT-D6 | -0.48 |  | -0.05 |  | -0.04 |  | *Foucher et al., ES&T, 2009* |
| Sediment | Gulf of Trieste | GT-1 | -0.30 |  | -0.08 |  | -0.08 |  | *Foucher et al., ES&T, 2009* |
| Sediment | Gulf of Trieste | GT-3 | -0.23 |  | -0.06 |  | -0.05 |  | *Foucher et al., ES&T, 2009* |
| Sediment | Gulf of Trieste | GT-A4 | -0.33 |  | -0.08 |  | -0.08 |  | *Foucher et al., ES&T, 2009* |
| Sediment | Gulf of Trieste | GT-AA1 | -0.33 |  | -0.05 |  | -0.06 |  | *Foucher et al., ES&T, 2009* |
| Sediment | Gulf of Trieste | GT-CZ | -0.47 |  | -0.04 |  | -0.01 |  | *Foucher et al., ES&T, 2009* |
| Sediment | Gulf of Trieste | GT-F1 | -2.53 |  | 0.01 |  | 0.12 |  | *Foucher et al., ES&T, 2009* |
| Sediment | Gulf of Trieste | GT-A3 | -1.43 |  | -0.02 |  | 0.00 |  | *Foucher et al., ES&T, 2009* |
| Sediment | Gulf of Trieste | GT-A20 | -0.38 |  | -0.04 |  | -0.09 |  | *Foucher et al., ES&T, 2009* |
| Sediment | Gulf of Trieste | GT-A28 | -1.19 |  | -0.02 |  | -0.06 |  | *Foucher et al., ES&T, 2009* |
| Sediment | Adriatic Sea - NE | Ad-STZB | -2.13 |  | 0.17 |  | 0.13 |  | *Foucher et al., ES&T, 2009* |
| Sediment | Adriatic Sea - NW | Ad-STZ6 | -1.49 |  | 0.00 |  | 0.07 |  | *Foucher et al., ES&T, 2009* |
| Sediment | Adriatic Sea - SW | Ad-STZ5 | -2.39 |  | 0.08 |  | 0.14 |  | *Foucher et al., ES&T, 2009* |
| Sediment | Central Portuguese Margin | 252-16(0~1) | -0.24 |  | 0.08 |  | -0.01 |  | *Mil-Homens et al., 2013* |
| Sediment | Central Portuguese Margin | 252-16(1~2) | -0.94 |  | 0.08 |  | 0.05 |  | *Mil-Homens et al., 2013* |
| Sediment | Central Portuguese Margin | 252-16(3~4) | -0.38 |  | 0.08 |  | 0.07 |  | *Mil-Homens et al., 2013* |
| Sediment | Central Portuguese Margin | 252-16(6~7) | -0.21 |  | 0.13 |  | 0.06 |  | *Mil-Homens et al., 2013* |
| Sediment | Central Portuguese Margin | 252-16(8~9) | -0.40 |  | 0.17 |  | 0.10 |  | *Mil-Homens et al., 2013* |
| Sediment | Central Portuguese Margin | 252-16(12~13) | -0.63 |  | 0.09 |  | -0.02 |  | *Mil-Homens et al., 2013* |
| Sediment | Central Portuguese Margin | 252-16(15~16) | -1.04 |  | 0.09 |  | 0.04 |  | *Mil-Homens et al., 2013* |
| Sediment | Central Portuguese Margin | 252-16(24~25) | -0.56 |  | 0.08 |  | 0.09 |  | *Mil-Homens et al., 2013* |
| Sediment | Central Portuguese Margin | 252-16(30~31) | -0.81 |  | 0.03 |  | 0.02 |  | *Mil-Homens et al., 2013* |
| Sediment | Central Portuguese Margin | 252-32(0~1) | -0.33 |  | 0.04 |  | 0.05 |  | *Mil-Homens et al., 2013* |
| Sediment | Central Portuguese Margin | 252-32(3~4) | -0.18 |  | 0.03 |  | -0.01 |  | *Mil-Homens et al., 2013* |
| Sediment | Central Portuguese Margin | 252-32(9~10) | -0.26 |  | 0.03 |  | 0.02 |  | *Mil-Homens et al., 2013* |
| Sediment | Central Portuguese Margin | 252-32(15~16) | -0.17 |  | -0.01 |  | -0.02 |  | *Mil-Homens et al., 2013* |
| Sediment | Central Portuguese Margin | 252-32(21~22) | -0.23 |  | -0.01 |  | 0.02 |  | *Mil-Homens et al., 2013* |
| Sediment | Central Portuguese Margin | 252-32(33~34) | -0.47 |  | 0.00 |  | -0.02 |  | *Mil-Homens et al., 2013* |
| Sediment | Central Portuguese Margin | 252-35(4-5) | -0.26 |  | 0.02 |  | 0.02 |  | *Mil-Homens et al., 2013* |
| Sediment | Central Portuguese Margin | 252-35(9-10) | -0.50 |  | 0.04 |  | -0.03 |  | *Mil-Homens et al., 2013* |
| Sediment | Central Portuguese Margin | 252-35(21-22) | -0.36 |  | -0.02 |  | -0.05 |  | *Mil-Homens et al., 2013* |
| Sediment | Central Portuguese Margin | 252-35(27-28) | -0.18 |  | -0.02 |  | -0.02 |  | *Mil-Homens et al., 2013* |
| Sediment | Central Portuguese Margin | 252-35(35-36) | -0.35 |  | -0.03 |  | -0.03 |  | *Mil-Homens et al., 2013* |
| Sediment | Central Portuguese Margin | 252-35(39-40) | -0.39 |  | -0.02 |  | 0.00 |  | *Mil-Homens et al., 2013* |
| Sediment | Pearl River Estuary | A8 | -1.06 |  | -0.03 |  | -0.03 |  | *Yin et al., 2015 EST* |
| Sediment | Pearl River Estuary | A9 | -1.64 |  | -0.01 |  | -0.01 |  | *Yin et al., 2015 EST* |
| Sediment | Pearl River Estuary | A10 | -1.62 |  | -0.08 |  | -0.09 |  | *Yin et al., 2015 EST* |
| Sediment | Pearl River Estuary | B1 | -0.80 |  | -0.06 |  | -0.08 |  | *Yin et al., 2015 EST* |
| Sediment | Pearl River Estuary | C1 | -1.39 |  | 0.04 |  | 0.03 |  | *Yin et al., 2015 EST* |
| Sediment | Pearl River Estuary | S1 | -0.86 |  | -0.02 |  | -0.06 |  | *Yin et al., 2015 EST* |
| Sediment | Pearl River Estuary | S2 | -1.04 |  | -0.06 |  | -0.05 |  | *Yin et al., 2015 EST* |
| Sediment | Pearl River Estuary | Y1 | -1.86 |  | 0.05 |  | 0.03 |  | *Yin et al., 2015 EST* |
| Sediment | Pearl River Estuary | Y2 | -0.68 |  | -0.03 |  | 0.00 |  | *Yin et al., 2015 EST* |
| Sediment | Pearl River Estuary | b2+ | -0.68 |  | -0.07 |  | -0.03 |  | *Yin et al., 2015 EST* |
| Sediment | Pearl River Estuary | A11 | -1.80 |  | -0.15 |  | -0.14 |  | *Yin et al., 2015 EST* |
| Sediment | Pearl River Estuary | A12 | -1.84 |  | 0.01 |  | 0.02 |  | *Yin et al., 2015 EST* |
| Sediment | Pearl River Estuary | A13 | -0.92 |  | -0.05 |  | -0.06 |  | *Yin et al., 2015 EST* |
| Sediment | Pearl River Estuary | A14 | -1.48 |  | -0.05 |  | -0.02 |  | *Yin et al., 2015 EST* |
| Sediment | Pearl River Estuary | B2 | -1.23 |  | -0.09 |  | -0.11 |  | *Yin et al., 2015 EST* |
| Sediment | Pearl River Estuary | S3 | -2.18 |  | 0.08 |  | 0.06 |  | *Yin et al., 2015 EST* |
| Sediment | Pearl River Estuary | B3 | -1.79 |  | -0.15 |  | -0.13 |  | *Yin et al., 2015 EST* |
| Sediment | Pearl River Estuary | b3+ | -0.94 |  | -0.06 |  | -0.06 |  | *Yin et al., 2015 EST* |
| Sediment | Pearl River Estuary | B4 | -1.75 |  | -0.08 |  | -0.08 |  | *Yin et al., 2015 EST* |
| Sediment | Pearl River Estuary | C2 | -1.89 |  | -0.10 |  | -0.09 |  | *Yin et al., 2015 EST* |
| Sediment | Pearl River Estuary | c2+ | -1.92 |  | 0.03 |  | 0.03 |  | *Yin et al., 2015 EST* |
| Sediment | Pearl River Estuary | C3 | -1.42 |  | -0.06 |  | -0.02 |  | *Yin et al., 2015 EST* |
| Sediment | Pearl River Estuary | Y3 | -1.53 |  | -0.13 |  | -0.16 |  | *Yin et al., 2015 EST* |
| Sediment | Pearl River Estuary | Y4 | -2.64 |  | 0.05 |  | 0.06 |  | *Yin et al., 2015 EST* |
| Sediment | Pearl River Estuary | Y5 | -0.97 |  | -0.05 |  | -0.03 |  | *Yin et al., 2015 EST* |
| Sediment | Pearl River Estuary | Y6 | -1.41 |  | -0.04 |  | 0.03 |  | *Yin et al., 2015 EST* |
| Sediment | Pearl River Estuary | Y8 | -1.32 |  | -0.04 |  | -0.06 |  | *Yin et al., 2015 EST* |
| Sediment | Pearl River Estuary | Y9 | -1.55 |  | -0.14 |  | -0.14 |  | *Yin et al., 2015 EST* |
| Sediment | Pearl River Estuary | Y12 | -1.48 |  | -0.05 |  | -0.09 |  | *Yin et al., 2015 EST* |
| Sediment | Pearl River Estuary | A15 | -2.75 |  | 0.12 |  | 0.08 |  | *Yin et al., 2015 EST* |
| Sediment | Pearl River Estuary | B5 | -1.55 |  | 0.07 |  | 0.05 |  | *Yin et al., 2015 EST* |
| Sediment | Pearl River Estuary | B6 | -1.87 |  | 0.11 |  | 0.10 |  | *Yin et al., 2015 EST* |
| Sediment | Pearl River Estuary | C4 | -1.96 |  | 0.10 |  | 0.11 |  | *Yin et al., 2015 EST* |
| Sediment | Pearl River Estuary | Y7 | -2.80 |  | 0.07 |  | 0.08 |  | *Yin et al., 2015 EST* |
| Sediment | Pearl River Estuary | Y11 | -1.77 |  | 0.08 |  | 0.07 |  | *Yin et al., 2015 EST* |
| Sediment | Pearl River Estuary | Y13 | -1.97 |  | 0.08 |  | 0.05 |  | *Yin et al., 2015 EST* |
| Sediment | Pearl River Estuary | Y15 | -2.74 |  | 0.16 |  | 0.17 |  | *Yin et al., 2015 EST* |
| Sediment | South China Sea | E403 | -2.56 |  | 0.30 |  | 0.29 |  | *Yin et al., 2015 EST* |
| Sediment | South China Sea | E416 | -2.10 |  | 0.21 |  | 0.25 |  | *Yin et al., 2015 EST* |
| Sediment | South China Sea | E109 | -2.16 |  | 0.42 |  | 0.39 |  | *Yin et al., 2015 EST* |
| Sediment | South China Sea | E407 | -2.50 |  | 0.45 |  | 0.36 |  | *Yin et al., 2015 EST* |
| Sediment | South China Sea | E406 | -2.52 |  | 0.34 |  | 0.31 |  | *Yin et al., 2015 EST* |
| Sediment | South China Sea | E201 | -2.82 |  | 0.39 |  | 0.38 |  | *Yin et al., 2015 EST* |
| Coal | China | ＳＨ-１ | -1.87 |  | 0.27 |  | 0.24 |  | *Biswas et al., 2008* |
| Coal | China | ＳＨ-１ | -2.62 |  | 0.37 |  | 0.29 |  | *Biswas et al., 2008* |
| Coal | China | ＳＨ-１ | -2.47 |  | 0.38 |  | 0.33 |  | *Biswas et al., 2008* |
| Coal | China | ＡＮ－１ | -0.64 |  | 0.10 |  | 0.14 |  | *Biswas et al., 2008* |
| Coal | China | ＨＥ-１ | -1.58 |  | -0.44 |  | -0.35 |  | *Biswas et al., 2008* |
| Coal | China | ＨＥ-１ | -2.70 |  | -0.39 |  | -0.36 |  | *Biswas et al., 2008* |
| Coal | China | ＨＥ-１ | -2.57 |  | -0.38 |  | -0.34 |  | *Biswas et al., 2008* |
| Coal | China | ＡＮ－２ | -0.64 |  | 0.07 |  | 0.10 |  | *Biswas et al., 2008* |
| Coal | China | ＡＮ－２ | -1.14 |  | 0.08 |  | 0.15 |  | *Biswas et al., 2008* |
| Coal | China | ＡＮ－３ | -0.43 |  | 0.02 |  | 0.04 |  | *Biswas et al., 2008* |
| Coal | China | ＡＮ－４ | -0.43 |  | 0.06 |  | 0.09 |  | *Biswas et al., 2008* |
| Coal | China | ＡＮ－５ | -0.28 |  | 0.08 |  | 0.11 |  | *Biswas et al., 2008* |
| Coal | China | ＪＩ－１ | -0.85 |  | -0.04 |  | -0.05 |  | *Biswas et al., 2008* |
| Coal | China | ＧＵ－１ | -1.37 |  | -0.02 |  | 0.00 |  | *Biswas et al., 2008* |
| Coal | China | ＨＢ-１ | -1.41 |  | -0.03 |  | 0.00 |  | *Biswas et al., 2008* |
| Coal | China | ＧＵ-２ | -1.22 |  | 0.04 |  | 0.04 |  | *Biswas et al., 2008* |
| Coal | U.S | ＡＬ-１ | -0.95 |  | -0.14 |  | -0.12 |  | *Biswas et al., 2008* |
| Coal | U.S | ＡＬ-１ | -0.98 |  | -0.14 |  | -0.14 |  | *Biswas et al., 2008* |
| Coal | U.S | ＡＬ-１ | -1.03 |  | -0.14 |  | -0.16 |  | *Biswas et al., 2008* |
| Coal | U.S | ＰＮ-１ | -1.55 |  | -0.12 |  | -0.13 |  | *Biswas et al., 2008* |
| Coal | U.S | ＰＮ-１ | -1.58 |  | -0.12 |  | -0.12 |  | *Biswas et al., 2008* |
| Coal | U.S | ＰＮ-１ | -1.28 |  | -0.12 |  | -0.17 |  | *Biswas et al., 2008* |
| Coal | U.S | ＴＸ-１ | -1.73 |  | -0.10 |  | -0.12 |  | *Biswas et al., 2008* |
| Coal | U.S | ＴＸ-１ | -1.58 |  | -0.10 |  | -0.15 |  | *Biswas et al., 2008* |
| Coal | U.S | ＴＸ-１ | -1.74 |  | -0.10 |  | -0.07 |  | *Biswas et al., 2008* |
| Coal | U.S | ＡＲ-１ | -2.98 |  | 0.08 |  | 0.05 |  | *Biswas et al., 2008* |
| Coal | U.S | ＣＯ－１ | -1.68 |  | -0.21 |  | -0.18 |  | *Biswas et al., 2008* |
| Coal | U.S | ＣＯ－１ | -1.62 |  | -0.17 |  | -0.19 |  | *Biswas et al., 2008* |
| Coal | U.S | ＣＯ－２ | -2.75 |  | 0.11 |  | 0.08 |  | *Biswas et al., 2008* |
| Coal | U.S | ＣＯ－２ | -2.68 |  | 0.09 |  | -0.03 |  | *Biswas et al., 2008* |
| Coal | U.S | ＣＯ－２ | -2.84 |  | 0.09 |  | 0.05 |  | *Biswas et al., 2008* |
| Coal | U.S | ＷＡ-１ | -0.88 |  | -0.34 |  | -0.35 |  | *Biswas et al., 2008* |
| Coal | U.S | ＵＴ－１ | -2.11 |  | -0.09 |  | -0.11 |  | *Biswas et al., 2008* |
| Coal | U.S | ＵＴ－１ | -1.90 |  | -0.07 |  | -0.10 |  | *Biswas et al., 2008* |
| Coal | U.S | ＵＴ－１ | -2.03 |  | -0.07 |  | -0.13 |  | *Biswas et al., 2008* |
| Coal | U.S | ＵＴ－１ | -2.26 |  | -0.07 |  | -0.10 |  | *Biswas et al., 2008* |
| Coal | U.S | ＡＫ－１ | -1.27 |  | -0.24 |  | -0.22 |  | *Biswas et al., 2008* |
| Coal | U.S | ＡＫ－１ | -1.37 |  | -0.23 |  | -0.23 |  | *Biswas et al., 2008* |
| Coal | U.S | ＡＲ－２ | -1.60 |  | -0.09 |  | -0.09 |  | *Biswas et al., 2008* |
| Coal | U.S | ＡＲ－２ | -1.37 |  | -0.10 |  | -0.10 |  | *Biswas et al., 2008* |
| Coal | U.S | ＯＫ－１ | -1.16 |  | 0.08 |  | 0.04 |  | *Biswas et al., 2008* |
| Coal | U.S | ＯＫ－１ | -1.23 |  | 0.08 |  | 0.06 |  | *Biswas et al., 2008* |
| Coal | U.S | ＯＫ－１ | -1.27 |  | 0.08 |  | 0.02 |  | *Biswas et al., 2008* |
| Coal | RUSSIA-KAZ | ＰＯ-１ | -1.49 |  | -0.49 |  | -0.43 |  | *Biswas et al., 2008* |
| Coal | RUSSIA-KAZ | ＰＯ-１ | -1.47 |  | -0.52 |  | -0.46 |  | *Biswas et al., 2008* |
| Coal | RUSSIA-KAZ | ＰＯ-２ | -0.25 |  | -0.52 |  | -0.49 |  | *Biswas et al., 2008* |
| Coal | RUSSIA-KAZ | ＰＯ-３ | -0.59 |  | -0.40 |  | -0.35 |  | *Biswas et al., 2008* |
| Coal | RUSSIA-KAZ | ＲＯ－１ | -0.20 |  | 0.00 |  | -0.01 |  | *Biswas et al., 2008* |
| Coal | RUSSIA-KAZ | ＲＯ－１ | -0.34 |  | 0.00 |  | -0.09 |  | *Biswas et al., 2008* |
| Coal | RUSSIA-KAZ | ＲＯ－１ | -0.25 |  | 0.00 |  | -0.08 |  | *Biswas et al., 2008* |
| Coal | RUSSIA-KAZ | ＫＫ－１ | -0.11 |  | -0.66 |  | -0.56 |  | *Biswas et al., 2008* |
| Coal | RUSSIA-KAZ | ＫＫ－１ | -0.39 |  | -0.63 |  | -0.53 |  | *Biswas et al., 2008* |
| Coal | RUSSIA-KAZ | ＫＫ－１ | -1.00 |  | -0.61 |  | -0.51 |  | *Biswas et al., 2008* |
| Coal | RUSSIA-KAZ | ＭＩＸ | -1.54 |  | -0.23 |  | -0.21 |  | *Biswas et al., 2008* |
| Coal | RUSSIA-KAZ | ＭＩＸ | -1.18 |  | -0.23 |  | -0.20 |  | *Biswas et al., 2008* |
| Coal | RUSSIA-KAZ | ＣＨ－１ | -0.99 |  | -0.22 |  | -0.22 |  | *Biswas et al., 2008* |
| Coal | RUSSIA-KAZ | ＣＨ－１ | -1.02 |  | -0.23 |  | -0.22 |  | *Biswas et al., 2008* |
| Coal | RUSSIA-KAZ | ＫＲ－１ | -1.61 |  | -0.28 |  | -0.33 |  | *Biswas et al., 2008* |
| Coal | RUSSIA-KAZ | ＫＲ－１ | -1.81 |  | -0.21 |  | -0.21 |  | *Biswas et al., 2008* |
| Coal | Illinois Basin | Ｈ６-ＷＨ１t | -1.46 |  | -0.18 |  | -0.22 |  | *Lefticariu et al., 2011* |
| Coal | Illinois Basin | Ｈ６-ＷＨ２m | -1.55 |  | -0.17 |  | -0.15 |  | *Lefticariu et al., 2011* |
| Coal | Illinois Basin | Ｈ６-ＷＨ３m | -1.17 |  | -0.16 |  | -0.13 |  | *Lefticariu et al., 2011* |
| Coal | Illinois Basin | Ｈ６-LV3m | -1.16 |  | -0.12 |  | -0.14 |  | *Lefticariu et al., 2011* |
| Coal | Illinois Basin | Ｈ６-LV3m* | -1.13 |  | -0.10 |  | -0.12 |  | *Lefticariu et al., 2011* |
| Coal | Illinois Basin | Ｈ６-B1t | -1.89 |  | -0.16 |  | -0.13 |  | *Lefticariu et al., 2011* |
| Coal | Illinois Basin | Ｈ６-B2m | -1.27 |  | -0.04 |  | -0.09 |  | *Lefticariu et al., 2011* |
| Coal | Illinois Basin | Ｈ６-B2m* | -1.26 |  | -0.07 |  | -0.05 |  | *Lefticariu et al., 2011* |
| Coal | Illinois Basin | Ｈ６-B3b | -1.74 |  | -0.12 |  | -0.12 |  | *Lefticariu et al., 2011* |
| Coal | Illinois Basin | Ｈ６-G1t | -1.51 |  | -0.02 |  | -0.06 |  | *Lefticariu et al., 2011* |
| Coal | Illinois Basin | Ｈ６-G2m | -2.16 |  | -0.10 |  | -0.10 |  | *Lefticariu et al., 2011* |
| Coal | Illinois Basin | Ｈ６-G3b | -1.44 |  | -0.05 |  | -0.06 |  | *Lefticariu et al., 2011* |
| Coal | Illinois Basin | Ｈ６-C3t | -1.91 |  | -0.17 |  | -0.14 |  | *Lefticariu et al., 2011* |
| Coal | Illinois Basin | Ｈ６-C3m | -1.58 |  | -0.23 |  | -0.18 |  | *Lefticariu et al., 2011* |
| Coal | Illinois Basin | Ｈ６-C3b | -1.57 |  | -0.19 |  | -0.21 |  | *Lefticariu et al., 2011* |
| Coal | Illinois Basin | SF5-WL1t | -1.59 |  | -0.02 |  | 0.02 |  | *Lefticariu et al., 2011* |
| Coal | Illinois Basin | SF5-WL1t* | -1.51 |  | 0.02 |  | 0.03 |  | *Lefticariu et al., 2011* |
| Coal | Illinois Basin | SF5-WL1m | -1.87 |  | 0.00 |  | 0.02 |  | *Lefticariu et al., 2011* |
| Coal | Illinois Basin | SF5-V1t | -1.21 |  | -0.02 |  | 0.00 |  | *Lefticariu et al., 2011* |
| Coal | Illinois Basin | SF5-V2m | -0.96 |  | 0.04 |  | 0.00 |  | *Lefticariu et al., 2011* |
| Coal | Illinois Basin | SF5-V3b | -0.75 |  | -0.01 |  | -0.01 |  | *Lefticariu et al., 2011* |
| Coal | Illinois Basin | MB-CP1t | -2.68 |  | -0.01 |  | 0.01 |  | *Lefticariu et al., 2011* |
| Coal | Illinois Basin | MB-CP2m | -2.46 |  | -0.02 |  | -0.04 |  | *Lefticariu et al., 2011* |
| Coal | Illinois Basin | MB-CP3b | -1.91 |  | 0.00 |  | -0.05 |  | *Lefticariu et al., 2011* |
| Coal | Illinois Basin | MTR-CP1t | -1.81 |  | -0.11 |  | -0.06 |  | *Lefticariu et al., 2011* |
| Coal | Illinois Basin | MTR-CP2m | -1.54 |  | -0.04 |  | -0.03 |  | *Lefticariu et al., 2011* |
| Coal | Illinois Basin | MTR-CP3m | -2.10 |  | -0.07 |  | -0.04 |  | *Lefticariu et al., 2011* |
| Coal | Illinois Basin | MTR-CP4b | -1.80 |  | -0.15 |  | -0.09 |  | *Lefticariu et al., 2011* |
| Coal | Illinois Basin | NIST 1632C | -1.86 |  | -0.04 |  | -0.03 |  | *Lefticariu et al., 2011* |
| Coal | USA |  | -1.31 |  | -0.11 |  | -0.01 |  | *Gehrke et al., 2012 EST* |
| Coal | USA |  | -0.79 |  | -0.31 |  | -0.23 |  | *Gehrke et al., 2012 EST* |
| Coal | USA |  | 0.45 |  | -0.34 |  | -0.30 |  | *Gehrke et al., 2012 EST* |
| Coal | USA |  | -0.75 |  | -0.23 |  | -0.17 |  | *Gehrke et al., 2012 EST* |
| Coal | USA |  | -1.22 |  | -0.21 |  | -0.24 |  | *Gehrke et al., 2012 EST* |
| Coal | Guizhou | GZ-1 | -1.41 |  | -0.03 |  | -0.07 |  | *Yin et al., 2014 EST* |
| Coal | Guizhou | GZ-2 | -1.09 |  | -0.04 |  | -0.05 |  | *Yin et al., 2014 EST* |
| Coal | Guizhou | GZ-3 | -0.66 |  | -0.05 |  | -0.03 |  | *Yin et al., 2014 EST* |
| Coal | Guizhou | GZ-4 | -1.00 |  | -0.05 |  | -0.08 |  | *Yin et al., 2014 EST* |
| Coal | Guizhou | GZ-5 | -0.77 |  | 0.05 |  | 0.01 |  | *Yin et al., 2014 EST* |
| Coal | Guizhou | GZ-6 | -1.08 |  | -0.06 |  | -0.03 |  | *Yin et al., 2014 EST* |
| Coal | Chongqing | CQ-1 | -0.40 |  | -0.09 |  | -0.12 |  | *Yin et al., 2014 EST* |
| Coal | Chongqing | CQ-2 | -0.18 |  | 0.03 |  | 0.03 |  | *Yin et al., 2014 EST* |
| Coal | Sichuan | SC-1 | -0.83 |  | -0.05 |  | -0.03 |  | *Yin et al., 2014 EST* |
| Coal | Sichuan | SC-2 | -0.88 |  | -0.02 |  | -0.03 |  | *Yin et al., 2014 EST* |
| Coal | Sichuan | SC-3 | -0.70 |  | 0.02 |  | -0.03 |  | *Yin et al., 2014 EST* |
| Coal | Sichuan | SC-4 | -0.59 |  | -0.02 |  | -0.05 |  | *Yin et al., 2014 EST* |
| Coal | Sichuan | SC-5 | -0.26 |  | 0.05 |  | -0.07 |  | *Yin et al., 2014 EST* |
| Coal | Yunnan | YN-1 | -1.53 |  | -0.06 |  | -0.04 |  | *Yin et al., 2014 EST* |
| Coal | Yunnan | YN-2 | -1.53 |  | -0.08 |  | 0.02 |  | *Yin et al., 2014 EST* |
| Coal | Guangxi | GX-1 | -0.96 |  | -0.05 |  | 0.01 |  | *Yin et al., 2014 EST* |
| Coal | Guangxi | GX-2 | -0.89 |  | -0.02 |  | 0.04 |  | *Yin et al., 2014 EST* |
| Coal | Liaoning | LN-1 | -0.81 |  | -0.02 |  | -0.04 |  | *Yin et al., 2014 EST* |
| Coal | Liaoning | LN-2 | -0.79 |  | 0.04 |  | 0.02 |  | *Yin et al., 2014 EST* |
| Coal | Liaoning | LN-3 | -0.65 |  | 0.01 |  | 0.00 |  | *Yin et al., 2014 EST* |
| Coal | Shandong | SD-1 | -0.80 |  | 0.00 |  | -0.04 |  | *Yin et al., 2014 EST* |
| Coal | Shandong | SD-2 | -0.69 |  | 0.05 |  | 0.08 |  | *Yin et al., 2014 EST* |
| Coal | Shandong | SD-3 | -0.26 |  | -0.05 |  | -0.07 |  | *Yin et al., 2014 EST* |
| Coal | Shandong | SD-4 | -0.38 |  | -0.01 |  | -0.04 |  | *Yin et al., 2014 EST* |
| Coal | Jiangsu | JS-1 | -0.44 |  | -0.08 |  | -0.09 |  | *Yin et al., 2014 EST* |
| Coal | Jiangsu | JS-2 | -0.16 |  | 0.07 |  | 0.05 |  | *Yin et al., 2014 EST* |
| Coal | Jiangsu | JS-3 | -0.92 |  | -0.06 |  | -0.06 |  | *Yin et al., 2014 EST* |
| Coal | Hebei | HB-1 | -1.47 |  | -0.07 |  | -0.09 |  | *Yin et al., 2014 EST* |
| Coal | Hebei | HB-2 | -1.00 |  | -0.04 |  | -0.06 |  | *Yin et al., 2014 EST* |
| Coal | Hebei | HB-3 | -1.05 |  | -0.04 |  | -0.01 |  | *Yin et al., 2014 EST* |
| Coal | Anhui | AH-1 | -0.47 |  | 0.10 |  | 0.07 |  | *Yin et al., 2014 EST* |
| Coal | Anhui | AH-2 | -0.71 |  | -0.24 |  | -0.19 |  | *Yin et al., 2014 EST* |
| Coal | Anhui | AH-3 | -0.93 |  | -0.05 |  | -0.03 |  | *Yin et al., 2014 EST* |
| Coal | Beijing | BJ-1 | -0.95 |  | 0.01 |  | -0.03 |  | *Yin et al., 2014 EST* |
| Coal | Heilongjiang | HLJ-1 | -1.11 |  | -0.20 |  | -0.19 |  | *Yin et al., 2014 EST* |
| Coal | In-Mongolia | NMG-1 | -0.65 |  | -0.17 |  | -0.14 |  | *Yin et al., 2014 EST* |
| Coal | In-Mongolia | NMG-2 | -1.13 |  | 0.15 |  | 0.11 |  | *Yin et al., 2014 EST* |
| Coal | In-Mongolia | NMG-3 | -0.85 |  | -0.18 |  | -0.16 |  | *Yin et al., 2014 EST* |
| Coal | In-Mongolia | NMG-4 | -1.93 |  | -0.38 |  | -0.37 |  | *Yin et al., 2014 EST* |
| Coal | In-Mongolia | NMG-5 | -1.00 |  | -0.10 |  | -0.16 |  | *Yin et al., 2014 EST* |
| Coal | Ningxia | NX-1 | -0.14 |  | -0.04 |  | -0.12 |  | *Yin et al., 2014 EST* |
| Coal | Xinjiang | XJ-1 | -1.43 |  | -0.24 |  | -0.24 |  | *Yin et al., 2014 EST* |
| Coal | Xinjiang | XJ-2 | -0.53 |  | -0.43 |  | -0.38 |  | *Yin et al., 2014 EST* |
| Coal | Xinjiang | XJ-3 | -0.86 |  | -0.36 |  | -0.31 |  | *Yin et al., 2014 EST* |
| Coal | Shaanxi | ShX-1 | -0.71 |  | -0.06 |  | -0.04 |  | *Yin et al., 2014 EST* |
| Coal | Shaanxi | ShX-2 | -0.79 |  | -0.03 |  | -0.03 |  | *Yin et al., 2014 EST* |
| Coal | Shaanxi | ShX-3 | -0.52 |  | -0.07 |  | -0.04 |  | *Yin et al., 2014 EST* |
| Coal | Shanxi | SX-1 | -1.49 |  | 0.06 |  | 0.07 |  | *Yin et al., 2014 EST* |
| Coal | Shanxi | SX-2 | -1.47 |  | 0.10 |  | 0.06 |  | *Yin et al., 2014 EST* |
| Coal | Shanxi | SX-3 | -1.75 |  | 0.25 |  | 0.20 |  | *Yin et al., 2014 EST* |
| Coal | Shanxi | SX-4 | -1.22 |  | 0.04 |  | 0.00 |  | *Yin et al., 2014 EST* |
| Coal | Shanxi | SX-5 | -1.36 |  | -0.01 |  | 0.04 |  | *Yin et al., 2014 EST* |
| Coal | Shanxi | SX-6 | -1.73 |  | -0.02 |  | 0.05 |  | *Yin et al., 2014 EST* |
| Coal | Shanxi | SX-7 | -2.26 |  | 0.33 |  | 0.38 |  | *Yin et al., 2014 EST* |
| Coal | Shanxi | SX-8 | -1.31 |  | -0.02 |  | -0.01 |  | *Yin et al., 2014 EST* |
| Coal | Henan | HN-1 | -1.60 |  | -0.24 |  | -0.20 |  | *Yin et al., 2014 EST* |
| Coal | Henan | HN-2 | -1.46 |  | -0.22 |  | -0.22 |  | *Yin et al., 2014 EST* |
| Coal | Henan | HN-3 | -0.65 |  | -0.10 |  | -0.14 |  | *Yin et al., 2014 EST* |
| Coal | Henan | HN-4 | -2.36 |  | -0.38 |  | -0.35 |  | *Yin et al., 2014 EST* |
| Coal | Henan | HN-5 | -0.38 |  | -0.25 |  | -0.21 |  | *Yin et al., 2014 EST* |
| Coal | Henan | HN-6 | -1.36 |  | -0.25 |  | -0.19 |  | *Yin et al., 2014 EST* |
| Coal | *Africa* | *Coal 1* | *-3.19* |  | *0.01* |  | *0.01* |  | *Sun et al., 2014 EST* |
| Coal | Africa | Coal 2 west B5 | -0.96 |  | -0.30 |  | -0.34 |  | *Sun et al., 2014 EST* |
| Coal | Africa | Coal 3 | -0.90 |  | -0.35 |  | -0.34 |  | *Sun et al., 2014 EST* |
| Coal | Africa | S-Africa | -1.62 |  | -0.27 |  | -0.43 |  | *Sun et al., 2014 EST* |
| Coal | Africa | SARM-20 | -1.09 |  | -0.38 |  | -0.43 |  | *Sun et al., 2014 EST* |
| Coal | Africa | Duvha | -1.25 |  | -0.34 |  | -0.33 |  | *Sun et al., 2014 EST* |
| Coal | Africa | Kriel | -1.25 |  | -0.28 |  | -0.30 |  | *Sun et al., 2014 EST* |
| Coal | Africa | Lethabo | -0.42 |  | -0.21 |  | -0.23 |  | *Sun et al., 2014 EST* |
| Coal | Africa | Majuba | -0.03 |  | -0.17 |  | -0.17 |  | *Sun et al., 2014 EST* |
| Coal | Africa | Camden | -0.73 |  | -0.22 |  | -0.25 |  | *Sun et al., 2014 EST* |
| Coal | Africa | Tutuka | -1.04 |  | -0.36 |  | -0.38 |  | *Sun et al., 2014 EST* |
| Coal | Africa | *Tutuka fresh ash* | *-1.82* |  | *-0.19* |  | *-0.19* |  | *Sun et al., 2014 EST* |
| Coal | Africa | *Tutuka old ash* | *-1.68* |  | *-0.39* |  | *-0.42* |  | *Sun et al., 2014 EST* |
| Coal | East Asia | Anhui-1 | 0.77 |  | -0.08 |  | -0.06 |  | *Sun et al., 2014 EST* |
| Coal | East Asia | Anhui-2 | -1.28 |  | 0.12 |  | 0.14 |  | *Sun et al., 2014 EST* |
| Coal | East Asia | Anhui-3 | -2.42 |  | 0.33 |  | 0.23 |  | *Sun et al., 2014 EST* |
| Coal | East Asia | Anhui-Huaian-4 | -0.61 |  | -0.04 |  | -0.05 |  | *Sun et al., 2014 EST* |
| Coal | East Asia | Anhui-Huaian-5 | -0.56 |  | 0.05 |  | 0.04 |  | *Sun et al., 2014 EST* |
| Coal | East Asia | Guiizhou-1 | -1.44 |  | 0.03 |  | -0.01 |  | *Sun et al., 2014 EST* |
| Coal | East Asia | Guizhou-2 | -1.37 |  | 0.02 |  | -0.02 |  | *Sun et al., 2014 EST* |
| Coal | East Asia | Guizhou-3 | -1.16 |  | 0.05 |  | -0.03 |  | *Sun et al., 2014 EST* |
| Coal | East Asia | Guizhou-4 | -2.47 |  | 0.13 |  | -0.06 |  | *Sun et al., 2014 EST* |
| Coal | East Asia | Guizhou-5 | -2.93 |  | 0.18 |  | 0.05 |  | *Sun et al., 2014 EST* |
| Coal | East Asia | Guizhou-6 | -0.89 |  | 0.23 |  | 0.18 |  | *Sun et al., 2014 EST* |
| Coal | East Asia | Hebei-1 | -1.93 |  | 0.02 |  | -0.02 |  | *Sun et al., 2014 EST* |
| Coal | East Asia | Hebei-2 | -2.64 |  | -0.08 |  | -0.05 |  | *Sun et al., 2014 EST* |
| Coal | East Asia | Hebei-3 | -0.20 |  | 0.02 |  | -0.01 |  | *Sun et al., 2014 EST* |
| Coal | East Asia | Henan-1 | -1.68 |  | -0.17 |  | -0.21 |  | *Sun et al., 2014 EST* |
| Coal | East Asia | Hubei-1 | -0.40 |  | 0.15 |  | 0.13 |  | *Sun et al., 2014 EST* |
| Coal | East Asia | Hubei-2 | 0.09 |  | 0.00 |  | -0.05 |  | *Sun et al., 2014 EST* |
| Coal | East Asia | Hubei-3 | -0.22 |  | 0.04 |  | -0.01 |  | *Sun et al., 2014 EST* |
| Coal | East Asia | Hubei-4 | -0.78 |  | -0.01 |  | 0.01 |  | *Sun et al., 2014 EST* |
| Coal | East Asia | Hubei-5 | -1.33 |  | -0.01 |  | -0.08 |  | *Sun et al., 2014 EST* |
| Coal | East Asia | IM-WL-33 | -3.07 |  | 0.21 |  | 0.13 |  | *Sun et al., 2014 EST* |
| Coal | East Asia | IM-WL-1 | -0.75 |  | 0.18 |  | 0.13 |  | *Sun et al., 2014 EST* |
| Coal | East Asia | Liaoning-1 | -0.60 |  | -0.06 |  | -0.08 |  | *Sun et al., 2014 EST* |
| Coal | East Asia | Shandong-1 | -1.92 |  | -0.01 |  | -0.05 |  | *Sun et al., 2014 EST* |
| Coal | East Asia | Shandong-2 | -1.15 |  | 0.06 |  | 0.05 |  | *Sun et al., 2014 EST* |
| Coal | East Asia | Shanxi-1 | -0.91 |  | -0.15 |  | -0.12 |  | *Sun et al., 2014 EST* |
| Coal | East Asia | Shanxi-2 | -1.01 |  | -0.09 |  | -0.08 |  | *Sun et al., 2014 EST* |
| Coal | East Asia | Shanxi-3 | -1.30 |  | -0.30 |  | -0.20 |  | *Sun et al., 2014 EST* |
| Coal | East Asia | Shanxi-4 | 0.49 |  | -0.16 |  | -0.09 |  | *Sun et al., 2014 EST* |
| Coal | East Asia | Shanxi-5 | -0.54 |  | -0.16 |  | -0.10 |  | *Sun et al., 2014 EST* |
| Coal | East Asia | Shanxi-6 | -0.29 |  | -0.21 |  | -0.17 |  | *Sun et al., 2014 EST* |
| Coal | East Asia | Xinjiang-1 | -0.32 |  | -0.30 |  | -0.25 |  | *Sun et al., 2014 EST* |
| Coal | East Asia | Yunnan-1 | -1.46 |  | -0.16 |  | -0.14 |  | *Sun et al., 2014 EST* |
| Coal | *West Europe* | *Cokes Carmeaux* | *-3.06* |  | *0.10* |  | *0.05* |  | *Sun et al., 2014 EST* |
| Coal | West Europe | Decazeville | -0.91 |  | -0.02 |  | -0.01 |  | *Sun et al., 2014 EST* |
| Coal | West Europe | Lignite Allemande | -1.55 |  | -0.46 |  | -0.48 |  | *Sun et al., 2014 EST* |
| Coal | East Europe | Romania-1 Valea de Brazi | -2.08 |  | -0.30 |  | -0.28 |  | *Sun et al., 2014 EST* |
| Coal | East Europe | Romania-2 Uricani | -0.73 |  | -0.44 |  | -0.38 |  | *Sun et al., 2014 EST* |
| Coal | East Europe | Romania-3 Barbateni | -1.12 |  | -0.40 |  | -0.40 |  | *Sun et al., 2014 EST* |
| Coal | East Europe | Romania-4 Barbateni | -1.35 |  | -0.37 |  | -0.38 |  | *Sun et al., 2014 EST* |
| Coal | East Europe | Romania-5 Lupeni | -1.15 |  | -0.34 |  | -0.31 |  | *Sun et al., 2014 EST* |
| Coal | East Europe | Romania-6 Paroseni | -1.67 |  | -0.33 |  | -0.37 |  | *Sun et al., 2014 EST* |
| Coal | East Europe | Romania-7 Vulcan | -1.85 |  | -0.36 |  | -0.36 |  | *Sun et al., 2014 EST* |
| Coal | East Europe | Romania-8 Aninoasa | -1.45 |  | -0.35 |  | -0.30 |  | *Sun et al., 2014 EST* |
| Coal | East Europe | Romania-9 Livezeni | -0.60 |  | -0.41 |  | -0.43 |  | *Sun et al., 2014 EST* |
| Coal | East Europe | Romania-10 Lonea | -1.44 |  | -0.41 |  | -0.37 |  | *Sun et al., 2014 EST* |
| Coal | East Europe | Romania-11 Petrila | 0.70 |  | -0.52 |  | -0.42 |  | *Sun et al., 2014 EST* |
| Coal | South Asia | JB-1 | -0.89 |  | 0.01 |  | -0.05 |  | *Sun et al., 2014 EST* |
| Coal | South Asia | JB-2 | -1.85 |  | 0.07 |  | 0.05 |  | *Sun et al., 2014 EST* |
| Coal | South Asia | JB-3 | -2.47 |  | 0.28 |  | 0.25 |  | *Sun et al., 2014 EST* |
| Coal | South Asia | JB-4 | -1.94 |  | 0.00 |  | -0.03 |  | *Sun et al., 2014 EST* |
| Coal | South Asia | JR-1 | -2.42 |  | 0.04 |  | -0.05 |  | *Sun et al., 2014 EST* |
| Coal | South Asia | JR-2 | -1.40 |  | -0.01 |  | 0.00 |  | *Sun et al., 2014 EST* |
| Coal | South Asia | JR-3 | -1.47 |  | -0.15 |  | -0.11 |  | *Sun et al., 2014 EST* |
| Coal | South Asia | JR-4 | -1.28 |  | 0.06 |  | 0.03 |  | *Sun et al., 2014 EST* |
| Coal | South Asia | RB-1 | -2.86 |  | 0.10 |  | 0.00 |  | *Sun et al., 2014 EST* |
| Coal | South Asia | RB-2 | -1.60 |  | -0.02 |  | -0.09 |  | *Sun et al., 2014 EST* |
| Coal | South Asia | RB-3 | -2.02 |  | 0.01 |  | -0.05 |  | *Sun et al., 2014 EST* |
| Coal | South Asia | WM-1 | -2.23 |  | 0.05 |  | 0.01 |  | *Sun et al., 2014 EST* |
| Coal | Southeast Asia | CQ01 | -0.40 |  | -0.04 |  | -0.06 |  | *Sun et al., 2014 EST* |
| Coal | Southeast Asia | CQ02 | -0.04 |  | -0.38 |  | -0.37 |  | *Sun et al., 2014 EST* |
| Coal | Southeast Asia | CQ03 | -0.97 |  | -0.17 |  | -0.17 |  | *Sun et al., 2014 EST* |
| Coal | Southeast Asia | CQ04 | -1.29 |  | -0.22 |  | -0.19 |  | *Sun et al., 2014 EST* |
| Coal | Southeast Asia | CQ05 | -1.18 |  | -0.15 |  | -0.17 |  | *Sun et al., 2014 EST* |
| Coal | Southeast Asia | CQ06 | -0.09 |  | -0.17 |  | -0.17 |  | *Sun et al., 2014 EST* |
| Coal | Southeast Asia | CQ07 | -0.42 |  | -0.42 |  | -0.35 |  | *Sun et al., 2014 EST* |
| Coal | Southeast Asia | CQ08 | 0.01 |  | -0.06 |  | -0.06 |  | *Sun et al., 2014 EST* |
| Coal | East&central Asia | Hov-I-1-11/02 | -0.89 |  | -0.39 |  | -0.33 |  | *Sun et al., 2014 EST* |
| Coal | East&central Asia | Chdg-2-B-10/02 | -1.21 |  | -0.42 |  | -0.36 |  | *Sun et al., 2014 EST* |
| Coal | East&central Asia | Nal-5-B-1-7/02 | 0.37 |  | -0.42 |  | -0.35 |  | *Sun et al., 2014 EST* |
| Coal | East&central Asia | Sivo-II-1-8/02 | 0.39 |  | -0.55 |  | -0.38 |  | *Sun et al., 2014 EST* |
| Coal | East&central Asia | Adnh-710-1-10/02 | -0.75 |  | -0.31 |  | -0.36 |  | *Sun et al., 2014 EST* |
| Coal | East&central Asia | Byne-28-1-8/02 | -1.77 |  | -0.32 |  | -0.35 |  | *Sun et al., 2014 EST* |
| Coal | East&central Asia | Byne-39-1-8/02 | -2.05 |  | -0.33 |  | -0.37 |  | *Sun et al., 2014 EST* |
| Coal | East&central Asia | Mogn-1-A-10/02 | -2.59 |  | -0.12 |  | -0.20 |  | *Sun et al., 2014 EST* |
| Coal | East&central Asia | Saio-6A-1-8/02 | 0.10 |  | -0.31 |  | -0.32 |  | *Sun et al., 2014 EST* |
| Coal | East&central Asia | Shar-Vln-1-9/02 | -0.08 |  | -0.18 |  | -0.20 |  | *Sun et al., 2014 EST* |
| Coal | East&central Asia | Nar-63-1-9/02 | -0.95 |  | -0.30 |  | -0.33 |  | *Sun et al., 2014 EST* |
| Coal | East&central Asia | Tav-4-1-6/02 | -2.13 |  | 0.22 |  | 0.07 |  | *Sun et al., 2014 EST* |
| Coal | East&central Asia | Tav-8-1-6/02 | -0.62 |  | 0.01 |  | -0.02 |  | *Sun et al., 2014 EST* |
| Coal | East&central Asia | Talb-I-1-2/03 | -1.50 |  | -0.25 |  | -0.32 |  | *Sun et al., 2014 EST* |
| Coal | East&central Asia | Talb-I-2-2/03 | -1.55 |  | -0.22 |  | -0.27 |  | *Sun et al., 2014 EST* |
| Coal | *Former USSR* | *R8 (catalyst)* | *-1.36* |  | *-0.05* |  | *-0.11* |  | *Sun et al., 2014 EST* |
| Coal | *Former USSR* | *R11-natural coke* | *-1.20* |  | *-0.02* |  | *-0.04* |  | *Sun et al., 2014 EST* |
| Coal | *Former USSR* | *R16-1-Shungite* | *-1.36* |  | *-0.02* |  | *-0.07* |  | *Sun et al., 2014 EST* |
| Coal | Former USSR | R1-1 | -2.52 |  | -0.17 |  | -0.20 |  | *Sun et al., 2014 EST* |
| Coal | Former USSR | R1-2 | -2.50 |  | -0.11 |  | -0.13 |  | *Sun et al., 2014 EST* |
| Coal | Former USSR | R3-1 | -2.77 |  | -0.14 |  | -0.17 |  | *Sun et al., 2014 EST* |
| Coal | Former USSR | R4 | -2.33 |  | -0.53 |  | -0.47 |  | *Sun et al., 2014 EST* |
| Coal | Former USSR | R5 | -1.75 |  | -0.08 |  | -0.12 |  | *Sun et al., 2014 EST* |
| Coal | Former USSR | R6 | 0.14 |  | 0.09 |  | 0.00 |  | *Sun et al., 2014 EST* |
| Coal | Former USSR | R7 | -0.55 |  | -0.23 |  | -0.23 |  | *Sun et al., 2014 EST* |
| Coal | Former USSR | R9 | -1.48 |  | -0.07 |  | -0.03 |  | *Sun et al., 2014 EST* |
| Coal | Former USSR | R10 | -3.46 |  | 0.15 |  | 0.06 |  | *Sun et al., 2014 EST* |
| Coal | Former USSR | R12 | -3.42 |  | 0.03 |  | -0.01 |  | *Sun et al., 2014 EST* |
| Coal | Former USSR | R13 | -1.36 |  | 0.02 |  | 0.00 |  | *Sun et al., 2014 EST* |
| Coal | Former USSR | R14 | -3.90 |  | -0.31 |  | -0.33 |  | *Sun et al., 2014 EST* |
| Coal | Former USSR | R15 | -2.47 |  | 0.20 |  | 0.17 |  | *Sun et al., 2014 EST* |
| Coal | North America | LMHS-1 | -1.54 |  | 0.06 |  | 0.03 |  | *Sun et al., 2014 EST* |
| Coal | North America | LLH-1 | -2.21 |  | 0.04 |  | -0.01 |  | *Sun et al., 2014 EST* |
| Coal | North America | LMA-1 | -1.44 |  | 0.06 |  | 0.05 |  | *Sun et al., 2014 EST* |
| Coal | North America | BV | -1.34 |  | -0.03 |  | -0.03 |  | *Sun et al., 2014 EST* |
|  |  |  |  |  |  |  |  |  |  |
| Rock (sedimentary) | California | 98HCL11 | -0.53 |  |  |  | -0.05 |  | *Smith et al., EPSL, 2008* |
| Rock (sedimentary) | California | GVBC-1 | -0.85 |  |  |  | -0.01 |  | *Smith et al., EPSL, 2008* |
| Rock (sedimentary) | California | GVBC-2 | -0.93 |  |  |  | 0.06 |  | *Smith et al., EPSL, 2008* |
| Rock (sedimentary) | California | GVBC-6 | -0.50 |  |  |  | 0.03 |  | *Smith et al., EPSL, 2008* |
| Rock (sedimentary) | California | GVBC-7 | -0.27 |  |  |  | 0.06 |  | *Smith et al., EPSL, 2008* |
| Rock (sedimentary) | California | GVSC-1 | -0.81 |  |  |  | 0.00 |  | *Smith et al., EPSL, 2008* |
| Rock (sedimentary) | California | GVSC-2 | -0.68 |  |  |  | 0.02 |  | *Smith et al., EPSL, 2008* |
| Rock (sedimentary) | California | GVSC-3 | -0.59 |  |  |  | 0.10 |  | *Smith et al., EPSL, 2008* |
| Rock (sedimentary) | California | GVSC-5 | -0.57 |  |  |  | -0.10 |  | *Smith et al., EPSL, 2008* |
| Rock (sedimentary) | California | GVSC-6 | -0.63 |  |  |  | 0.11 |  | *Smith et al., EPSL, 2008* |
| Rock (sedimentary) | California | GVSC-7 | -0.34 |  |  |  | 0.06 |  | *Smith et al., EPSL, 2008* |
| Rock (sedimentary) | California | GVSC-8 | -0.17 |  |  |  | 0.04 |  | *Smith et al., EPSL, 2008* |
| Rock (sedimentary) | California | GVSC-9 | -0.68 |  |  |  | 0.05 |  | *Smith et al., EPSL, 2008* |
| Rock (sedimentary) | California | KF-1 | -0.91 |  |  |  | 0.28 |  | *Smith et al., EPSL, 2008* |
| Rock (sedimentary) | California | KF-2 | -0.93 |  |  |  | -0.02 |  | *Smith et al., EPSL, 2008* |
| Rock (metamorphic) | California | CR-1 | -0.65 |  |  |  | -0.09 |  | *Smith et al., EPSL, 2008* |
| Rock (metamorphic) | California | F1 | -0.71 |  |  |  | -0.04 |  | *Smith et al., EPSL, 2008* |
| Rock (metamorphic) | California | F2 | -1.21 |  |  |  | 0.01 |  | *Smith et al., EPSL, 2008* |
| Rock (metamorphic) | California | F3 | -0.11 |  |  |  | -0.07 |  | *Smith et al., EPSL, 2008* |
| Rock (metamorphic) | California | F4 | -0.43 |  |  |  | -0.07 |  | *Smith et al., EPSL, 2008* |
| Rock (metamorphic) | California | F5 | -0.32 |  |  |  | 0.10 |  | *Smith et al., EPSL, 2008* |
| Rock (metamorphic) | California | F6 | -0.88 |  |  |  | 0.12 |  | *Smith et al., EPSL, 2008* |
| Rock (metamorphic) | California | F7 | 0.20 |  |  |  | 0.02 |  | *Smith et al., EPSL, 2008* |
| Rock (metamorphic) | California | SB-1 | 1.61 |  |  |  | -0.12 |  | *Smith et al., EPSL, 2008* |
| Rock (metamorphic) | California | SB-2 | -0.58 |  |  |  | -0.06 |  | *Smith et al., EPSL, 2008* |
| Rock (metamorphic) | California | Serp. Reiff Rd. | -1.70 |  |  |  | 0.02 |  | *Smith et al., EPSL, 2008* |
| Rock (volcanic) | California | 98HCL17 | -0.25 |  |  |  | -0.08 |  | *Smith et al., EPSL, 2008* |
| Rock (volcanic) | California | 98HCL12 | -0.69 |  |  |  | -0.08 |  | *Smith et al., EPSL, 2008* |
| Rock (volcanic) | California | 97HCL8 | -0.74 |  |  |  | -0.08 |  | *Smith et al., EPSL, 2008* |
| Rock (volcanic) | California | SB-16 | -1.20 |  |  |  | -0.13 |  | *Smith et al., EPSL, 2008* |
| Rock (volcanic) | California | 98HCL4 | -0.60 |  |  |  | -0.13 |  | *Smith et al., EPSL, 2008* |
| Rock (volcanic) | California | 98HCL14 | -0.56 |  |  |  | 0.07 |  | *Smith et al., EPSL, 2008* |
| Rock (volcanic) | California | 98HCL2 | -0.57 |  |  |  | -0.06 |  | *Smith et al., EPSL, 2008* |
| Rock (volcanic) | California | 98HCL13 | -0.49 |  |  |  | 0.13 |  | *Smith et al., EPSL, 2008* |
| Rock (volcanic) | California | 01HCL01 | -0.46 |  |  |  | -0.04 |  | *Smith et al., EPSL, 2008* |
|  |  |  |  |  |  |  |  |  |  |
| Hg ore | Nevada | CLEM-1 | -0.45 |  |  |  | 0.01 |  | *Smith et al., Geology, 2005* |
| Hg ore | Nevada | CLEM-1 (rep) | -0.45 |  |  |  | 0.01 |  | *Smith et al., Geology, 2005* |
| Hg ore | Nevada | BM-10 | -2.37 |  |  |  | 0.15 |  | *Smith et al., Geology, 2005* |
| Hg ore | Nevada | BM-11 | -3.05 |  |  |  | 0.14 |  | *Smith et al., Geology, 2005* |
| Hg ore | Nevada | BM-12 | -1.43 |  |  |  | 0.06 |  | *Smith et al., Geology, 2005* |
| Hg ore | Nevada | BM-13 | -0.47 |  |  |  | 0.06 |  | *Smith et al., Geology, 2005* |
| Hg ore | Nevada | BNMS-D | -1.66 |  |  |  | 0.09 |  | *Smith et al., Geology, 2005* |
| Hg ore | Nevada | BNMS-E | -3.54 |  |  |  | 0.20 |  | *Smith et al., Geology, 2005* |
| Hg ore | Nevada | VL-1 | -0.89 |  |  |  | 0.03 |  | *Smith et al., Geology, 2005* |
| Hg ore | Nevada | VL-2 | -1.27 |  |  |  | 0.10 |  | *Smith et al., Geology, 2005* |
| Hg ore | Nevada | VL-3 | -0.60 |  |  |  | 0.03 |  | *Smith et al., Geology, 2005* |
| Hg ore | Nevada | VL-10 | -0.62 |  |  |  | 0.00 |  | *Smith et al., Geology, 2005* |
| Hg ore | Nevada | BU-1 | -0.93 |  |  |  | 0.05 |  | *Smith et al., Geology, 2005* |
| Hg ore | Nevada | BU-2 | -1.00 |  |  |  | 0.08 |  | *Smith et al., Geology, 2005* |
| Hg ore | Nevada | KA-2 | -0.97 |  |  |  | 0.04 |  | *Smith et al., Geology, 2005* |
| Hg ore | Nevada | BM-9 | -0.33 |  |  |  | 0.03 |  | *Smith et al., Geology, 2005* |
| Hg ore | Nevada | BUCK02-1A1 | 0.34 |  |  |  | -0.01 |  | *Smith et al., Geology, 2005* |
| Hg ore | Nevada | BUCK02-1A2 | 1.99 |  |  |  | 0.11 |  | *Smith et al., Geology, 2005* |
| Hg ore | Nevada | BUCK02-1A3 | 0.24 |  |  |  | 0.11 |  | *Smith et al., Geology, 2005* |
| Hg ore | Nevada | BUCK02-5 | 1.81 |  |  |  | -0.05 |  | *Smith et al., Geology, 2005* |
| Hydrothermal | Nevada | BN-5 352 | -0.21 |  |  |  | 0.00 |  | *Smith et al., Geology, 2005* |
| Hydrothermal | Nevada | BM-14 | 0.45 |  |  |  | 0.05 |  | *Smith et al., Geology, 2005* |
| Hydrothermal | Nevada | BVPD03-1 | -1.43 |  |  |  | 0.08 |  | *Smith et al., Geology, 2005* |
| Hydrothermal | Nevada | BVPD03-2 | 1.20 |  |  |  | 0.00 |  | *Smith et al., Geology, 2005* |
| Hydrothermal | Nevada | IH76-902 | -0.76 |  |  |  | -0.05 |  | *Smith et al., Geology, 2005* |
| Hg ore | California | OH-1 | -0.91 |  |  |  | 0.09 |  | *Smith et al., EPSL, 2008* |
| Hg ore | California | OH-2 | -0.25 |  |  |  | 0.04 |  | *Smith et al., EPSL, 2008* |
| Hg ore | California | OH-4 | -3.85 |  |  |  | 0.26 |  | *Smith et al., EPSL, 2008* |
| Hg ore | California | OH-6 | -0.23 |  |  |  | 0.02 |  | *Smith et al., EPSL, 2008* |
| Hg ore | California | USGS-BC-1 | 0.55 |  |  |  | -0.04 |  | *Smith et al., EPSL, 2008* |
| Hg ore | California | USGS-BC-2 | -0.33 |  |  |  | 0.01 |  | *Smith et al., EPSL, 2008* |
| Hg ore | California | CTC-1 | 0.67 |  |  |  | -0.02 |  | *Smith et al., EPSL, 2008* |
| Hg ore | California | CTC-2 | 0.73 |  |  |  | -0.01 |  | *Smith et al., EPSL, 2008* |
| Hg ore | California | CM-1 | -0.36 |  |  |  | 0.04 |  | *Smith et al., EPSL, 2008* |
| Hg ore | California | CM-3 | -0.80 |  |  |  | 0.05 |  | *Smith et al., EPSL, 2008* |
| Hg ore | California | CM-4 | -0.76 |  |  |  | 0.06 |  | *Smith et al., EPSL, 2008* |
| Hg ore | California | USGS-CB-2 | -0.31 |  |  |  | 0.03 |  | *Smith et al., EPSL, 2008* |
| Hg ore | California | CB-3 | -0.83 |  |  |  | 0.09 |  | *Smith et al., EPSL, 2008* |
| Hg ore | California | CB-4 | -0.40 |  |  |  | 0.06 |  | *Smith et al., EPSL, 2008* |
| Hg ore | California | CB-5 | -0.62 |  |  |  | 0.07 |  | *Smith et al., EPSL, 2008* |
| Hg ore | California | CB-6 | -0.65 |  |  |  | 0.06 |  | *Smith et al., EPSL, 2008* |
| Hg ore | California | USGS-SCHTZ-2 | 0.38 |  |  |  | -0.04 |  | *Smith et al., EPSL, 2008* |
| Hg ore | California | USGS-SCHTZ-1 | 0.09 |  |  |  | -0.03 |  | *Smith et al., EPSL, 2008* |
| Hg ore | California | USGS-AET-1 | -0.51 |  |  |  | 0.04 |  | *Smith et al., EPSL, 2008* |
| Hg ore | California | USGS-AET-2 | -1.00 |  |  |  | 0.08 |  | *Smith et al., EPSL, 2008* |
| Hg ore | California | SB-4 | -0.50 |  |  |  | 0.04 |  | *Smith et al., EPSL, 2008* |
| Hg ore | California | SB-5 | -1.12 |  |  |  | 0.08 |  | *Smith et al., EPSL, 2008* |
| Hg ore | California | SB-6 | -0.65 |  |  |  | 0.06 |  | *Smith et al., EPSL, 2008* |
| Hg ore | California | SB-7 | -0.79 |  |  |  | 0.04 |  | *Smith et al., EPSL, 2008* |
| Hg ore | California | USGS-SB-1 | -0.18 |  |  |  | 0.03 |  | *Smith et al., EPSL, 2008* |
| Hg ore | California | CS0516 | -0.59 |  |  |  | 0.03 |  | *Smith et al., EPSL, 2008* |
| Hg ore | California | CS0517 | -0.88 |  |  |  | 0.01 |  | *Smith et al., EPSL, 2008* |
| Hg ore | California | CS0505 | -0.34 |  |  |  | 0.02 |  | *Smith et al., EPSL, 2008* |
| Hg ore | California | MZ-1 | -1.12 |  |  |  | 0.15 |  | *Smith et al., EPSL, 2008* |
| Hg ore | California | MZ-2 | 0.04 |  |  |  | 0.03 |  | *Smith et al., EPSL, 2008* |
| Hg ore | California | MZ-3 | -0.13 |  |  |  | 0.04 |  | *Smith et al., EPSL, 2008* |
| Hg ore | California | MZ-5 | -0.06 |  |  |  | 0.04 |  | *Smith et al., EPSL, 2008* |
| Hg ore | California | MZ-6 | -1.77 |  |  |  | 0.15 |  | *Smith et al., EPSL, 2008* |
| Hg ore | California | MZ-7 | 0.22 |  |  |  | 0.04 |  | *Smith et al., EPSL, 2008* |
| Hg ore | California | MZ-8 | -0.47 |  |  |  | 0.11 |  | *Smith et al., EPSL, 2008* |
| Hg ore | California | USGS-ABT-1 | -0.21 |  |  |  | 0.04 |  | *Smith et al., EPSL, 2008* |
| Hg ore | California | CS0507 | -2.34 |  |  |  | 0.15 |  | *Smith et al., EPSL, 2008* |
| Hg ore | California | CS0508 | -0.55 |  |  |  | 0.03 |  | *Smith et al., EPSL, 2008* |
| Hg ore | California | CS0509 | -3.69 |  |  |  | 0.41 |  | *Smith et al., EPSL, 2008* |
| Hg ore | California | CS0510 | -3.54 |  |  |  | 0.32 |  | *Smith et al., EPSL, 2008* |
| Hg ore | California | CS0511 | -0.75 |  |  |  | 0.01 |  | *Smith et al., EPSL, 2008* |
| Hg ore | California | CS0503 | -1.12 |  |  |  | 0.05 |  | *Smith et al., EPSL, 2008* |
| Hg ore | California | CS0504 | -0.01 |  |  |  | -0.03 |  | *Smith et al., EPSL, 2008* |
| Hg ore | California | CS0514 | -0.36 |  |  |  | 0.01 |  | *Smith et al., EPSL, 2008* |
| Hg ore | California | CS0513 | -1.58 |  |  |  | 0.06 |  | *Smith et al., EPSL, 2008* |
| Hg ore | California | USGS-MAN-4 | 0.00 |  |  |  | 0.06 |  | *Smith et al., EPSL, 2008* |
| Hg ore | California | USGS-MAN-5 | -0.65 |  |  |  | 0.07 |  | *Smith et al., EPSL, 2008* |
| Hg ore | California | USGS-MAN-6 | -0.78 |  |  |  | 0.04 |  | *Smith et al., EPSL, 2008* |
| Hg ore | California | USGS-MAN-8 | -0.28 |  |  |  | 0.02 |  | *Smith et al., EPSL, 2008* |
| Hg ore | California | USGS-MAN-1 | 0.07 |  |  |  | -0.03 |  | *Smith et al., EPSL, 2008* |
| Hg ore | California | USGS-MAN-9 | -0.25 |  |  |  | 0.00 |  | *Smith et al., EPSL, 2008* |
| Hg ore | California | USGS-MAN-7 | -0.90 |  |  |  | 0.08 |  | *Smith et al., EPSL, 2008* |
| Hg ore | California | KM-1 | -0.42 |  |  |  | 0.00 |  | *Smith et al., EPSL, 2008* |
| Hg ore | California | KM-2 | -1.32 |  |  |  | 0.14 |  | *Smith et al., EPSL, 2008* |
| Hg ore | California | KM-3 | -0.68 |  |  |  | 0.08 |  | *Smith et al., EPSL, 2008* |
| Hg ore | California | KM-4 | -0.34 |  |  |  | 0.05 |  | *Smith et al., EPSL, 2008* |
| Hg ore | California | KM-5 | -0.42 |  |  |  | 0.07 |  | *Smith et al., EPSL, 2008* |
| Hg ore | California | KM-6 | -1.18 |  |  |  | 0.11 |  | *Smith et al., EPSL, 2008* |
| Hg ore | California | RM-10 | -1.62 |  |  |  | 0.15 |  | *Smith et al., EPSL, 2008* |
| Hg ore | California | RM-4 | -0.92 |  |  |  | 0.09 |  | *Smith et al., EPSL, 2008* |
| Hg ore | California | RM-5 | -1.30 |  |  |  | 0.12 |  | *Smith et al., EPSL, 2008* |
| Hg ore | California | RM-7 | -1.15 |  |  |  | 0.13 |  | *Smith et al., EPSL, 2008* |
| Hg ore | California | RM-8 | -0.65 |  |  |  | 0.07 |  | *Smith et al., EPSL, 2008* |
| Hg ore | California | RM-9 | -0.22 |  |  |  | 0.17 |  | *Smith et al., EPSL, 2008* |
| Hg ore | California | SS-3 | -2.52 |  |  |  | 0.22 |  | *Smith et al., EPSL, 2008* |
| Hg ore | California | USGS-BAK-1 | -1.05 |  |  |  | 0.09 |  | *Smith et al., EPSL, 2008* |
| Hg ore | California | USGS-HAR-1 | -0.16 |  |  |  | 0.02 |  | *Smith et al., EPSL, 2008* |
| Hg ore | California | USGS-HAR-2 | -0.22 |  |  |  | 0.02 |  | *Smith et al., EPSL, 2008* |
| Hg ore | California | 66782 | -0.39 |  |  |  | 0.03 |  | *Smith et al., EPSL, 2008* |
| Hg ore | California | 66783 | -0.26 |  |  |  | 0.06 |  | *Smith et al., EPSL, 2008* |
| Hg ore | California | 66784 | -0.16 |  |  |  | 0.05 |  | *Smith et al., EPSL, 2008* |
| Hg ore | California | 98579-2 | -0.23 |  |  |  | 0.03 |  | *Smith et al., EPSL, 2008* |
| Hg ore | California | 98582-1 | -1.27 |  |  |  | 0.12 |  | *Smith et al., EPSL, 2008* |
| Hg ore | California | 98582-2 | -1.36 |  |  |  | 0.16 |  | *Smith et al., EPSL, 2008* |
| Hg ore | California | 98583-2 | -0.60 |  |  |  | 0.08 |  | *Smith et al., EPSL, 2008* |
| Hg ore | California | 98583-5 | 0.15 |  |  |  | 0.00 |  | *Smith et al., EPSL, 2008* |
| Hg ore | California | 66441 | 0.74 |  |  |  | -0.03 |  | *Smith et al., EPSL, 2008* |
| Hg ore | California | 66444 | -0.61 |  |  |  | 0.10 |  | *Smith et al., EPSL, 2008* |
| Hg ore | California | 66441-b | 0.44 |  |  |  | 0.02 |  | *Smith et al., EPSL, 2008* |
| Hg ore | California | 15107-2 | -1.23 |  |  |  | 0.11 |  | *Smith et al., EPSL, 2008* |
| Hg ore | California | 51621-3 | -0.04 |  |  |  | 0.01 |  | *Smith et al., EPSL, 2008* |
| Hg ore | California | 51621-4 | -0.15 |  |  |  | 0.03 |  | *Smith et al., EPSL, 2008* |
| Hg ore | California | 51621-5 | -0.10 |  |  |  | 0.05 |  | *Smith et al., EPSL, 2008* |
| Hg ore | California | 51621-7 | -0.16 |  |  |  | 0.05 |  | *Smith et al., EPSL, 2008* |
| Hg ore | California | 98616-2 | -0.97 |  |  |  | 0.09 |  | *Smith et al., EPSL, 2008* |
| Hg ore | California | 98616-3 | 0.85 |  |  |  | -0.04 |  | *Smith et al., EPSL, 2008* |
| Hg ore | California | 98617-1 | -0.34 |  |  |  | 0.03 |  | *Smith et al., EPSL, 2008* |
| Hydrothermal | California | Geysers-1 | -1.18 |  |  |  | 0.05 |  | *Smith et al., EPSL, 2008* |
| Hydrothermal | California | Geysers-2 | -0.70 |  |  |  | 0.03 |  | *Smith et al., EPSL, 2008* |
| Hydrothermal | California | 99SM1s | -0.85 |  |  |  | 0.05 |  | *Smith et al., EPSL, 2008* |
| Hydrothermal | California | AHS-1 | -1.56 |  |  |  | 0.16 |  | *Smith et al., EPSL, 2008* |
| Hydrothermal | California | AHS-2 | -1.02 |  |  |  | 0.04 |  | *Smith et al., EPSL, 2008* |
| Hydrothermal | California | AHS-5 | -0.98 |  |  |  | 0.03 |  | *Smith et al., EPSL, 2008* |
| Hydrothermal | California | TRS-1 | -1.45 |  |  |  | 0.10 |  | *Smith et al., EPSL, 2008* |
| Hydrothermal | California | ELB-1 | -1.60 |  |  |  | 0.14 |  | *Smith et al., EPSL, 2008* |
| Hydrothermal | California | WS-1 | -1.20 |  |  |  | 0.11 |  | *Smith et al., EPSL, 2008* |
| Hydrothermal | California | WS-1 (rep) | -1.39 |  |  |  | 0.12 |  | *Smith et al., EPSL, 2008* |
| Hydrothermal | California | Jones' Ftn.-1 | -1.50 |  |  |  | 0.16 |  | *Smith et al., EPSL, 2008* |
| Hydrothermal | California | Blanck-1 | -2.04 |  |  |  | 0.22 |  | *Smith et al., EPSL, 2008* |
| Hydrothermal | California | WS-3 | -0.95 |  |  |  | 0.11 |  | *Smith et al., EPSL, 2008* |
| Hydrothermal | California | ELG-1 | -3.03 |  |  |  | 0.28 |  | *Smith et al., EPSL, 2008* |
| Hydrothermal | California | ELG-2 | -3.42 |  |  |  | 0.32 |  | *Smith et al., EPSL, 2008* |
| Hydrothermal | California | BSS-1 | -2.11 |  |  |  | 0.15 |  | *Smith et al., EPSL, 2008* |
| Hydrothermal | California | 99MPS02 | -0.48 |  |  |  | 0.04 |  | *Smith et al., EPSL, 2008* |
| Hydrothermal | California | 99MPS02 (rep) | -0.21 |  |  |  | 0.04 |  | *Smith et al., EPSL, 2008* |
| Ore | Idrija Mine | Idrija ore-black | 0.23 |  | -0.11 |  | -0.13 |  | *Foucher et al., EST, 2009* |
| Ore | Idrija Mine | Idrija ore-red | -0.26 |  | -0.05 |  | -0.01 |  | *Foucher et al., EST, 2009* |
| Ore |  | Dunet | -0.48 |  | 0.08 |  | -0.07 |  | *Sonke et al., Chem. Geol. 2010* |
| Ore |  | lgue de Mas | -0.44 |  | -0.05 |  | -0.11 |  | *Sonke et al., Chem. Geol. 2010* |
| Ore |  | Cerons | 0.29 |  | -0.13 |  | -0.14 |  | *Sonke et al., Chem. Geol. 2010* |
| Ore |  | Dunet-low-fine | -0.33 |  | -0.11 |  | -0.14 |  | *Sonke et al., Chem. Geol. 2010* |
| Ore | Mount Isa, Australia | Mt. lsa 3 | -1.25 |  | 0.05 |  | -0.01 |  | *Sonke et al., Chem. Geol. 2010* |
| Ore | Mount Isa, Australia | Mt. lsa 4 | -0.58 |  | 0.03 |  | -0.05 |  | *Sonke et al., Chem. Geol. 2010* |
| Ore | Broken Hill, Australia | BH-1 | 0.46 |  | -0.12 |  | -0.19 |  | *Sonke et al., Chem. Geol. 2010* |
| Ore | Kipushi, Congo | 8300.00 | -1.13 |  | -0.07 |  | -0.10 |  | *Sonke et al., Chem. Geol. 2010* |
| Ore | Kipushi, Congo | 8957.00 | -0.62 |  | -0.08 |  | -0.10 |  | *Sonke et al., Chem. Geol. 2010* |
| Ore | Kipushi, Congo | 13092.00 | -1.41 |  | -0.08 |  | -0.08 |  | *Sonke et al., Chem. Geol. 2010* |
| Ore | Picos del Europa,Spain | Picos | -0.81 |  | 0.00 |  | 0.06 |  | *Sonke et al., Chem. Geol. 2010* |
| Ore | Wildcat Hills | H6 -WH 1t pyrite | -0.14 |  | -0.05 |  | -0.02 |  | *Lefticariu et al., EST 2011* |
| Ore | Wildcat Hills | H6 -WH 2t pyrite* | -0.14 |  | -0.01 |  | -0.03 |  | *Lefticariu et al., EST 2011* |
| Ore | Viper | SF-V2m pyrite | -0.05 |  | 0.04 |  | 0.01 |  | *Lefticariu et al., EST 2011* |
| Ore | Wanshan mercury mine | Hg ore-1 | -1.06 |  | 0.01 |  | 0.01 |  | *Feng et al., EST 2010* |
| Ore | Wanshan mercury mine | Hg ore-2 | -1.06 |  | 0.06 |  | 0.01 |  | *Feng et al., EST 2010* |
| Ore | Wanshan mercury mine | Hg ore-3 | -0.85 |  | -0.02 |  | -0.05 |  | *Feng et al., EST 2010* |
| Cinnabar | Wanshan mercury mine | Cin.-WS-1 | -0.75 |  | 0.03 |  | -0.03 |  | *Yin et al., Chem. Geol. 2013a* |
| Cinnabar | Wanshan mercury mine | Cin.-WS-2 | -0.92 |  | -0.02 |  | 0.03 |  | *Yin et al., Chem. Geol. 2013a* |
| Cinnabar | Wanshan mercury mine | Cin.-WS-3 | -0.60 |  | -0.01 |  | -0.02 |  | *Yin et al., Chem. Geol. 2013a* |
| Cinnabar | Wanshan mercury mine | Cin.-WS-4 | -0.71 |  | 0.01 |  | -0.03 |  | *Yin et al., Chem. Geol. 2013a* |
| Cinnabar | Wanshan mercury mine | Cin.-WS-5 | -0.66 |  | 0.01 |  | 0.00 |  | *Yin et al., Chem. Geol. 2013a* |
| Cinnabar | Wanshan mercury mine | Cin.-WS-6 | -0.89 |  | -0.02 |  | 0.02 |  | *Yin et al., Chem. Geol. 2013a* |
| Cinnabar | Wanshan mercury mine | Cin.-WS-7 | -0.82 |  | 0.00 |  | 0.03 |  | *Yin et al., Chem. Geol. 2013a* |
| Cinnabar | Wanshan mercury mine | Cin.-WS-9 | -0.80 |  | 0.05 |  | 0.04 |  | *Yin et al., Chem. Geol. 2013a* |
| Cinnabar | Wanshan mercury mine | Cin.-WS-10 | -0.79 |  | 0.02 |  | 0.04 |  | *Yin et al., Chem. Geol. 2013a* |
| Cinnabar | Wanshan mercury mine | Cin.-WS-11 | -0.70 |  | 0.01 |  | 0.01 |  | *Yin et al., Chem. Geol. 2013a* |
| Cinnabar | Wanshan mercury mine | Cin.-WS-12 | -0.56 |  | 0.02 |  | 0.01 |  | *Yin et al., Chem. Geol. 2013a* |
| Cinnabar | Wanshan mercury mine | Cin.-WS-13 | -0.78 |  | 0.02 |  | -0.03 |  | *Yin et al., Chem. Geol. 2013a* |
| Cinnabar | Wanshan mercury mine | Cin.-WS-14 | -0.68 |  | 0.01 |  | -0.02 |  | *Yin et al., Chem. Geol. 2013a* |
| Fluid | Ojo Caliente | OC-1 | 0.10 |  | 0.11 |  |  |  | *Sherman et al., 2009 EPSL* |
| Fluid | Ojo Caliente | OC-2 | 0.14 |  | 0.11 |  |  |  | *Sherman et al., 2009 EPSL* |
| Fluid | Ojo Caliente | OC-3 | 0.11 |  | 0.16 |  |  |  | *Sherman et al., 2009 EPSL* |
| Fluid | Ojo Caliente | OC-4 | 0.32 |  | 0.12 |  |  |  | *Sherman et al., 2009 EPSL* |
| Fluid | Ojo Caliente | OC-5 | 0.28 |  | 0.14 |  |  |  | *Sherman et al., 2009 EPSL* |
| Fluid | Ojo Caliente | OC-6 | 0.58 |  | 0.16 |  |  |  | *Sherman et al., 2009 EPSL* |
| Fluid | Mud Volcano | YNP-1 | -0.47 |  | 0.09 |  |  |  | *Sherman et al., 2009 EPSL* |
| Fluid | Mud Volcano | YNP-2 | -0.49 |  | 0.04 |  |  |  | *Sherman et al., 2009 EPSL* |
| Fluid | Mud Volcano | YNP-3 | -1.02 |  | -0.02 |  |  |  | *Sherman et al., 2009 EPSL* |
| Sinter | Ojo Caliente | OC-S-2 | -0.50 |  | 0.27 |  |  |  | *Sherman et al., 2009 EPSL* |
| Sinter | Ojo Caliente | OC-S-4 | -0.26 |  | 0.19 |  |  |  | *Sherman et al., 2009 EPSL* |
| Sinter | Ojo Caliente | OC-S-5 | 0.36 |  | 0.07 |  |  |  | *Sherman et al., 2009 EPSL* |
| Sinter | Ojo Caliente | OC-S-6 | -0.22 |  | 0.10 |  |  |  | *Sherman et al., 2009 EPSL* |
|  |  |  |  |  |  |  |  |  |  |
| Condensates | Vulcano Island (Italy) | FA | -0.38 |  | 0.02 |  | 0.05 |  | *Zambardi et al., EPSL 2009* |
| Condensates | Vulcano Island (Italy) | FA | -0.15 |  | -0.01 |  | 0.02 |  | *Zambardi et al., EPSL 2009* |
| Condensates | Vulcano Island (Italy) | F0 | -0.71 |  | 0.07 |  | 0.03 |  | *Zambardi et al., EPSL 2009* |
| Condensates | Vulcano Island (Italy) | F0 | -0.86 |  | 0.15 |  | 0.04 |  | *Zambardi et al., EPSL 2009* |
| Condensates | Vulcano Island (Italy) | F0 | -0.80 |  | 0.03 |  | -0.04 |  | *Zambardi et al., EPSL 2009* |
| Condensates | Vulcano Island (Italy) | F5 | -1.09 |  | -0.05 |  | -0.10 |  | *Zambardi et al., EPSL 2009* |
| Condensates | Vulcano Island (Italy) | F11 | -0.68 |  | 0.11 |  | 0.05 |  | *Zambardi et al., EPSL 2009* |
| Condensates | Vulcano Island (Italy) | F11 | -0.67 |  | 0.05 |  | -0.04 |  | *Zambardi et al., EPSL 2009* |
| Condensates | Vulcano Island (Italy) | F11 | -1.03 |  | 0.22 |  | 0.07 |  | *Zambardi et al., EPSL 2009* |
| HgIIp | Vulcano Island (Italy) | F0 | 0.10 |  | -0.08 |  | -0.10 |  | *Zambardi et al., EPSL 2009* |
| HgIIp | Vulcano Island (Italy) | F0 | -0.12 |  | 0.01 |  | 0.10 |  | *Zambardi et al., EPSL 2009* |
| HgIIp | Vulcano Island (Italy) | F11 | -0.22 |  | -0.11 |  | -0.17 |  | *Zambardi et al., EPSL 2009* |
| HgIIp | Vulcano Island (Italy) | F11 | -0.30 |  | 0.04 |  | -0.02 |  | *Zambardi et al., EPSL 2009* |
| HgIIp | Vulcano Island (Italy) | FA | 0.02 |  | -0.11 |  | -0.09 |  | *Zambardi et al., EPSL 2009* |
| HgIIp | Vulcano Island (Italy) | FA | -0.01 |  | -0.03 |  | -0.08 |  | *Zambardi et al., EPSL 2009* |
| HgIIp | Vulcano Island (Italy) | F5 | -0.09 |  | 0.09 |  | 0.05 |  | *Zambardi et al., EPSL 2009* |
| HgIIp | Vulcano Island (Italy) | F5 | -0.13 |  | -0.07 |  | 0.03 |  | *Zambardi et al., EPSL 2009* |
| HgIIp | Vulcano Island (Italy) | F5 | -0.06 |  | 0.03 |  | 0.02 |  | *Zambardi et al., EPSL 2009* |
| HgIIp | Vulcano Island (Italy) | F5 | -0.33 |  | 0.00 |  | 0.03 |  | *Zambardi et al., EPSL 2009* |
| Au trap Hg0g | Vulcano Island (Italy) | F0 | -1.73 |  | -0.12 |  | -0.22 |  | *Zambardi et al., EPSL 2009* |
| Cinnabar | McDermitt | MCD-100 | -0.70 |  | 0.01 |  | -0.01 |  | *Stetson et al., EST, 2009* |
| Cinnabar | Terlingua | MCD-101 | -0.64 |  | 0.02 |  | 0.09 |  | *Stetson et al., EST, 2009* |
| Cinnabar | Terlingua | MCD-102a | -0.52 |  | 0.00 |  | 0.07 |  | *Stetson et al., EST, 2009* |
| Cinnabar | Terlingua | MCD-102b | -0.56 |  | -0.04 |  | -0.01 |  | *Stetson et al., EST, 2009* |
| Cinnabar | Terlingua | MCD-102c | -0.50 |  | -0.03 |  | 0.01 |  | *Stetson et al., EST, 2009* |
| Cinnabar | Terlingua | MCD-102d | -0.42 |  | 0.00 |  | 0.01 |  | *Stetson et al., EST, 2009* |
| Cinnabar | Terlingua | MCD-102e | -0.61 |  | 0.04 |  | 0.01 |  | *Stetson et al., EST, 2009* |
| Cinnabar | Terlingua | MCD-102f | -0.69 |  | -0.01 |  | 0.06 |  | *Stetson et al., EST, 2009* |
| Cinnabar | Terlingua | MCD-103a | -0.58 |  | 0.03 |  | 0.04 |  | *Stetson et al., EST, 2009* |
| Cinnabar | Terlingua | MCD-103b | -0.60 |  | 0.00 |  | -0.03 |  | *Stetson et al., EST, 2009* |
| Cinnabar | Terlingua | MCD-104 | -0.61 |  | 0.02 |  | 0.03 |  | *Stetson et al., EST, 2009* |
| Hg ore | New Idria Hg Mine | NI ore | -0.26 |  | 0.02 |  | 0.02 |  | *Wiederhold et al., EST 2013* |
| Hg ore | New Idria Hg Mine | O1g | -0.09 |  | 0.00 |  | 0.01 |  | *Wiederhold et al., EST 2013* |
| Hg ore | New Idria Hg Mine | O1r | -0.08 |  | -0.03 |  | 0.00 |  | *Wiederhold et al., EST 2013* |
| Hg ore | New Idria Hg Mine | O2 | -0.03 |  | 0.03 |  | 0.00 |  | *Wiederhold et al., EST 2013* |
| Hg ore | New Idria Hg Mine | O3 | -0.06 |  | -0.03 |  | 0.00 |  | *Wiederhold et al., EST 2013* |
| Hg ore | New Idria Hg Mine | O4 | 0.02 |  | 0.00 |  | 0.02 |  | *Wiederhold et al., EST 2013* |
| Hg ore | New Idria Hg Mine | O5a | 0.16 |  | -0.05 |  | -0.01 |  | *Wiederhold et al., EST 2013* |
| Hg ore | New Idria Hg Mine | O5b | 0.07 |  | -0.03 |  | -0.07 |  | *Wiederhold et al., EST 2013* |
| Hg ore | New Idria Hg Mine | O6 | -0.03 |  | -0.04 |  | -0.04 |  | *Wiederhold et al., EST 2013* |
| Hg ore | New Idria Hg Mine | O7a | -0.08 |  | 0.02 |  | -0.01 |  | *Wiederhold et al., EST 2013* |
| Hg ore | New Idria Hg Mine | O7b | 0.09 |  | -0.02 |  | -0.01 |  | *Wiederhold et al., EST 2013* |
| Cinnabar ore | HgS Colin | H1 | -0.86 |  | -0.03 |  | -0.07 |  | *Cook et al., EST 2013* |
| Cinnabar ore | HgS Earl | H2 | 0.12 |  | -0.15 |  | -0.14 |  | *Cook et al., EST 2013* |
| Cinnabar ore | MGL #34990 | H3 | -1.40 |  | 0.08 |  | -0.03 |  | *Cook et al., EST 2013* |
| Cinnabar ore | BM1917, 510 | H4 | -0.39 |  | -0.06 |  | -0.06 |  | *Cook et al., EST 2013* |
| Cinnabar ore | BM1917, 511 | H5 | -2.44 |  | 0.21 |  | 0.06 |  | *Cook et al., EST 2013* |
| Cinnabar ore | Cat# 17090 | C1 | 0.79 |  | -0.17 |  | -0.17 |  | *Cook et al., EST 2013* |
| Cinnabar ore | BM1983, MI7325 | C2 | -0.40 |  | 0.08 |  | -0.02 |  | *Cook et al., EST 2013* |
| Cinnabar ore | BM1985, MI25138 | C3 | 0.25 |  | 0.21 |  | 0.14 |  | *Cook et al., EST 2013* |
| Cinnabar ore | BM1985, MI10916 | C4 | -0.31 |  | -0.13 |  | -0.10 |  | *Cook et al., EST 2013* |
| Cinnabar ore | MGL #92603 | C5 | -1.24 |  | 0.06 |  | -0.03 |  | *Cook et al., EST 2013* |
| Cinnabar ore | BM33451 | C6 | -0.84 |  | 0.14 |  | 0.02 |  | *Cook et al., EST 2013* |
| Cinnabar ore | BM86464 | C7 | 0.31 |  | 0.04 |  | 0.02 |  | *Cook et al., EST 2013* |
| Cinnabar ore | KW11 96KW G-S73 | A1 | -0.32 |  | -0.09 |  | -0.08 |  | *Cook et al., EST 2013* |
| Cinnabar ore | KW13 G-S28 | A2 | 0.12 |  | -0.03 |  | -0.09 |  | *Cook et al., EST 2013* |
| Cinnabar ore | 161971.000A | A3 | -0.61 |  | -0.08 |  | -0.11 |  | *Cook et al., EST 2013* |
| Cinnabar ore | 161971.001B | A4 | -0.89 |  | -0.04 |  | -0.07 |  | *Cook et al., EST 2013* |
| Cinnabar ore | 161971.002C | A5 | -0.42 |  | -0.08 |  | -0.12 |  | *Cook et al., EST 2013* |
| Cinnabar ore | 161971.003D | A6 | -0.39 |  | -0.04 |  | -0.09 |  | *Cook et al., EST 2013* |
| Cinnabar ore | 04LH-A-X919 | A7 | -0.38 |  | -0.02 |  | -0.04 |  | *Cook et al., EST 2013* |
| Cinnabar ore | 4CBN BN4E22-23 | A8 | -1.11 |  | 0.06 |  | 0.00 |  | *Cook et al., EST 2013* |
| Cinnabar ore | 197.271.35 | A9 | -0.66 |  | -0.06 |  | -0.12 |  | *Cook et al., EST 2013* |
| Cinnabar ore | 245642.000 | A10 | -0.20 |  | -0.03 |  | 0.01 |  | *Cook et al., EST 2013* |
| Cinnabar ore | 245643.000 | A11 | -0.54 |  | 0.00 |  | -0.09 |  | *Cook et al., EST 2013* |
| Cinnabar ore | 1994.35.24 | A12 | 0.22 |  | -0.12 |  | -0.14 |  | *Cook et al., EST 2013* |
| Cinnabar ore | 41.2/516/01 | A13 | -0.67 |  | -0.02 |  | -0.05 |  | *Cook et al., EST 2013* |
| Cinnabar ore | B/9180/03 | A14 | -2.00 |  | 0.17 |  | 0.05 |  | *Cook et al., EST 2013* |
| Cinnabar ore | 1979.206.1025 | A15 | -0.06 |  | 0.12 |  | 0.05 |  | *Cook et al., EST 2013* |
| Cinnabar ore | 1979.206.1026 | A16 | 0.05 |  | 0.12 |  | 0.12 |  | *Cook et al., EST 2013* |
| Cinnabar ore | 163779.000 | A17 | -1.56 |  | 0.27 |  | 0.20 |  | *Cook et al., EST 2013* |
|  |  |  |  |  |  |  |  |  |  |
|  |  |  |  |  |  |  |  |  |  |
| Chimney piece | Guaymas Basin | GB-1 | -0.31 |  | 0.00 |  |  |  | *Sherman et al., 2009 EPSL* |
| fluid precipitate | Guaymas Basin | GB-2 | -0.37 |  | 0.04 |  |  |  | *Sherman et al., 2009 EPSL* |
| Chimney piece | Guaymas Basin | GB-3 | -0.01 |  | 0.03 |  |  |  | *Sherman et al., 2009 EPSL* |
|  |  |  |  |  |  |  |  |  |  |
| Hg0 |  |  | 0.93 |  | -0.15 |  | -0.06 |  | *Demers et al., 2013* |
| Hg0 |  |  | 1.23 |  | -0.18 |  | -0.20 |  | *Demers et al., 2013* |
| Hg0 |  |  | 0.48 |  | -0.21 |  | -0.13 |  | *Demers et al., 2013* |
| Hg0 |  |  | 0.64 |  | -0.21 |  | -0.14 |  | *Demers et al., 2013* |
| Hg0 |  |  | 0.83 |  | -0.21 |  | -0.18 |  | *Demers et al., 2013* |
| Hg0 |  |  | 1.60 |  | -0.25 |  | -0.12 |  | *Demers et al., 2013* |
| Hg0 |  |  | 0.82 |  | -0.12 |  | -0.19 |  | *Demers et al., 2013* |
| Hg0 |  |  | 0.74 |  | -0.22 |  | -0.14 |  | *Demers et al., 2013* |
| Hg0 |  |  | 1.32 |  | -0.24 |  | -0.18 |  | *Demers et al., 2013* |
| Hg0 |  |  | 0.74 |  | -0.17 |  | -0.13 |  | *Demers et al., 2013* |
| Hg0 |  |  | 0.60 |  | -0.19 |  | -0.05 |  | *Demers et al., 2013* |
| Hg0 |  |  | 1.33 |  | -0.18 |  | -0.14 |  | *Demers et al., 2013* |
| Hg0 |  |  | -2.39 |  | -0.20 |  | -0.14 |  | *Rolison et al., 2013* |
| Hg0 |  |  | -0.33 |  | -0.26 |  | -0.20 |  | *Rolison et al., 2013* |
| Hg0 |  |  | -1.31 |  | -0.14 |  | -0.05 |  | *Rolison et al., 2013* |
| Hg0 |  |  | -3.88 |  | -0.21 |  | -0.20 |  | *Rolison et al., 2013* |
| Hg0 |  |  | -3.58 |  | -0.41 |  | -0.32 |  | *Rolison et al., 2013* |
| Hg0 |  |  | -3.22 |  | -0.27 |  | -0.25 |  | *Rolison et al., 2013* |
| Hg0 |  |  | -2.88 |  | -0.29 |  | -0.23 |  | *Rolison et al., 2013* |
| Hg0 |  |  | -3.24 |  | -0.37 |  | -0.25 |  | *Rolison et al., 2013* |
| Hg0 |  |  | -3.28 |  | -0.28 |  | -0.29 |  | *Rolison et al., 2013* |
| Hg0 |  |  | -3.67 |  | -0.03 |  | -0.04 |  | *Rolison et al., 2013* |
| Hg0 |  |  | -3.45 |  | -0.20 |  | -0.18 |  | *Rolison et al., 2013* |
| Hg0 |  |  | -2.63 |  | -0.26 |  | -0.23 |  | *Rolison et al., 2013* |
| Hg0 |  |  | -1.27 |  | -0.28 |  | -0.27 |  | *Rolison et al., 2013* |
| Hg0 |  |  | 0.48 |  | -0.04 |  | -0.12 |  | *Gratz et al., 2010* |
| Hg0 |  |  | -0.39 |  | -0.17 |  | -0.06 |  | *Gratz et al., 2010* |
| Hg0 |  |  | 0.27 |  | -0.06 |  | 0.00 |  | *Gratz et al., 2010* |
| Hg0 |  |  | 0.41 |  | -0.07 |  | -0.09 |  | *Gratz et al., 2010* |
| Hg0 |  |  | 0.27 |  | 0.00 |  | -0.02 |  | *Gratz et al., 2010* |
| Hg0 |  |  | 0.25 |  | -0.10 |  | -0.08 |  | *Gratz et al., 2010* |
| Hg0 |  |  | -0.59 |  | 0.06 |  | 0.03 |  | *Gratz et al., 2010* |
| Hg0 |  |  | -0.12 |  | -0.11 |  | -0.14 |  | *Sherman et al., 2010* |
| Hg0 |  |  | 0.15 |  | -0.22 |  | -0.18 |  | *Sherman et al., 2010* |
| Hg0 | Dexte | DXT-VP-1 | 0.48 |  | -0.04 |  | -0.12 |  | *Gratz et al., 2010* |
| Hg0 | Dexte | DXT-VP-2 | -0.39 |  | -0.17 |  | -0.06 |  | *Gratz et al., 2010* |
| Hg0 | Dexte | DXT-VP-3 | 0.27 |  | -0.06 |  | 0.00 |  | *Gratz et al., 2010* |
| Hg0 | Dexte | DXT-VP-4 | 0.41 |  | -0.07 |  | -0.09 |  | *Gratz et al., 2010* |
| Hg0 | Dexte | DXT-VP-5 | 0.27 |  | 0.00 |  | -0.02 |  | *Gratz et al., 2010* |
| Hg0 | Dexte | DXT-VP-6 | 0.25 |  | -0.10 |  | -0.08 |  | *Gratz et al., 2010* |
| Hg0 | Chicago | UOC-VP-1 | -0.59 |  | 0.06 |  | 0.03 |  | *Gratz et al., 2010* |
| Gaseous HgT | Arctic | Gaseous HgT | -0.12 |  | -0.11 |  | -0.14 |  | *Sherman et al., 2010 Nature Geosci* |
| Gaseous HgT | Arctic | Gaseous HgT | 0.15 |  | -0.22 |  | -0.18 |  | *Sherman et al., 2010 Nature Geosci* |
| Gaseous HgT | Arctic | Chamber expt Emitted Hg0 | -0.38 |  | -1.87 |  | -1.97 |  | *Sherman et al., 2010 Nature Geosci* |
|  |  |  |  |  |  |  |  |  |  |
| Snow | Arctic | Snowfall | 0.54 |  | -0.95 |  | -0.81 |  | *Sherman et al., 2010 Nature Geosci* |
| Snow | Arctic | Snowfall | -0.05 |  | -1.20 |  | -1.21 |  | *Sherman et al., 2010 Nature Geosci* |
| Snow | Arctic | Surface snow | 0.70 |  | -2.41 |  | -2.23 |  | *Sherman et al., 2010 Nature Geosci* |
| Snow | Arctic | Surface snow | -0.10 |  | -2.49 |  | -2.52 |  | *Sherman et al., 2010 Nature Geosci* |
| Snow | Arctic | Surface snow | -0.49 |  | -2.63 |  | -2.76 |  | *Sherman et al., 2010 Nature Geosci* |
| Snow | Arctic | Drifted snow | 0.57 |  | -3.84 |  | -3.70 |  | *Sherman et al., 2010 Nature Geosci* |
| Snow | Arctic | Drifted snow | 0.40 |  | -4.93 |  | -4.83 |  | *Sherman et al., 2010 Nature Geosci* |
| Snow | Arctic | Drifted snow | 0.44 |  | -5.08 |  | -4.97 |  | *Sherman et al., 2010 Nature Geosci* |
| Snow | Arctic | Chamber expt HgIIpre | 0.40 |  | -4.93 |  | -4.83 |  | *Sherman et al., 2010 Nature Geosci* |
| Snow | Arctic | Chamber expt HgIIpre | 0.44 |  | -5.08 |  | -4.97 |  | *Sherman et al., 2010 Nature Geosci* |
| Snow | Arctic | Chamber expt HgIIpost | 0.73 |  | -5.34 |  | -5.16 |  | *Sherman et al., 2010 Nature Geosci* |
| Snow | Arctic | Chamber expt HgIIpost | 0.70 |  | -5.52 |  | -5.34 |  | *Sherman et al., 2010 Nature Geosci* |
| Snow |  | Wet frost flowers | 0.36 |  | 0.86 |  | 0.47 |  | *Sherman et al., 2012 JGR* |
| Snow |  | Wet frost flowers | 0.47 |  | 0.67 |  | 0.39 |  | *Sherman et al., 2012 JGR* |
| Snow |  | Dry frost flowers | 0.50 |  | 0.24 |  | 0.09 |  | *Sherman et al., 2012 JGR* |
| Snow |  | Dry frost flowers | 0.59 |  | 0.05 |  | -0.12 |  | *Sherman et al., 2012 JGR* |
| Snow |  | Dry frost flowers 48h old | 0.49 |  | 0.13 |  | -0.02 |  | *Sherman et al., 2012 JGR* |
| Snow |  | Dry frost flowers 48h old | 0.53 |  | 0.06 |  | 0.02 |  | *Sherman et al., 2012 JGR* |
| Snow |  | Suirface snow | 0.69 |  | -1.01 |  | -1.00 |  | *Sherman et al., 2012 JGR* |
| Snow |  | Suirface snow | 0.63 |  | -0.97 |  | -0.88 |  | *Sherman et al., 2012 JGR* |
| Snow |  | Dry frost flowers | -0.28 |  | -0.36 |  | -0.40 |  | *Sherman et al., 2012 JGR* |
| Snow |  | Dry frost flowers | -0.29 |  | -0.28 |  | -0.14 |  | *Sherman et al., 2012 JGR* |
| Snow |  | Brine | -0.39 |  | -0.39 |  | -0.23 |  | *Sherman et al., 2012 JGR* |
| Snow |  | Brine | -0.19 |  | -0.41 |  | -0.33 |  | *Sherman et al., 2012 JGR* |
|  |  |  |  |  |  |  |  |  |  |
| Hg2+ |  |  | 1.41 |  | -0.07 |  | -0.03 |  | *Rolison et al., 2013* |
| Hg2+ |  |  | 1.45 |  | -0.07 |  | -0.06 |  | *Rolison et al., 2013* |
| Hg2+ |  |  | 1.29 |  | -0.10 |  | -0.04 |  | *Rolison et al., 2013* |
| Hg2+ |  |  | 1.57 |  | -0.24 |  | -0.07 |  | *Rolison et al., 2013* |
| Hg2+ |  |  | 0.51 |  | 0.03 |  | -0.02 |  | *Rolison et al., 2013* |
| Hg2+ |  |  | 1.58 |  | -0.09 |  | -0.13 |  | *Rolison et al., 2013* |
| Hg2+ |  |  | 1.19 |  | -0.20 |  | -0.25 |  | *Rolison et al., 2013* |
| Hg2+ |  |  | 1.23 |  | -0.26 |  | -0.23 |  | *Rolison et al., 2013* |
| Hg2+ |  |  | 0.79 |  | 0.18 |  | 0.19 |  | *Rolison et al., 2013* |
| Hg2+ |  |  | 1.61 |  | -0.28 |  | -0.22 |  | *Rolison et al., 2013* |
|  |  |  |  |  |  |  |  |  |  |
| Hgp |  |  | -0.92 |  | 0.56 |  | 0.48 |  | *Rolison et al., 2013* |
| Hgp |  |  | -1.61 |  | 0.39 |  | 0.37 |  | *Rolison et al., 2013* |
| Hgp |  |  | -0.12 |  | 0.89 |  | 0.81 |  | *Rolison et al., 2013* |
| Hgp |  |  | -0.98 |  | 0.75 |  | 0.66 |  | *Rolison et al., 2013* |
| Hgp |  |  | -1.19 |  | 0.86 |  | 0.68 |  | *Rolison et al., 2013* |
| Hgp |  |  | -0.79 |  | 1.27 |  | 1.13 |  | *Rolison et al., 2013* |
| Hgp |  |  | -0.85 |  | 0.71 |  | 0.62 |  | *Rolison et al., 2013* |
| Hgp |  |  | -0.28 |  | 1.16 |  | 1.01 |  | *Rolison et al., 2013* |
| Hgp |  |  | -1.44 |  | 1.36 |  | 1.20 |  | *Rolison et al., 2013* |
| Hgp |  |  | -0.49 |  | 0.36 |  | 0.30 |  | *Rolison et al., 2013* |

**Table S2** Mercury isotopic compositions of the sphalerites in China

| **Sample ID** | **δ^202^Hg** | **2σ** | **Δ^199^Hg** | **2σ** | **Δ^200^Hg** | **2σ** | **Δ^201^Hg** | **2σ** | **Deposit** |
| --- | --- | --- | --- | --- | --- | --- | --- | --- | --- |
|  | **(‰)** | **(‰)** | **(‰)** | **(‰)** | **(‰)** | **(‰)** | **(‰)** | **(‰)** | **Types** |
| S-1 | -0.31 | 0.16 | 0.05 | 0.01 | 0.02 | 0.02 | 0.01 | 0.05 | SEDEX |
| S-2 | -0.5 | 0.04 | 0.08 | 0.01 | 0.01 | 0.02 | 0.08 | 0.07 | SEDEX |
| S-3 | -0.17 | 0.07 | 0.02 | 0.01 | -0.02 | 0.02 | 0.06 | 0.03 | SEDEX |
| S-4 | -0.57 | 0.08 | 0.09 | 0.04 | -0.01 | 0.03 | 0.14 | 0.03 | SEDEX |
| S-5 | -1.6 | 0.14 | 0.12 | 0 | 0.01 | 0.02 | 0.06 | 0.01 | SEDEX |
| S-6 | -0.58 | 0.06 | 0.18 | 0.04 | 0.02 | 0.03 | 0.18 | 0.02 | SEDEX |
| S-7 | 0.23 | 0.11 | 0.04 | 0.04 | 0.00 | 0.03 | 0.05 | 0.01 | SEDEX |
| S-8 | -0.79 | 0.11 | -0.02 | 0.03 | 0.02 | 0.03 | -0.01 | 0.03 | SEDEX |
| S-9 | -1.07 | 0.09 | -0.08 | 0.01 | 0.02 | 0.03 | -0.07 | 0.01 | SEDEX |
| S-10 | -0.69 | 0.13 | 0.08 | 0.01 | -0.02 | 0.03 | 0.06 | 0.04 | SEDEX |
| S-11 | -0.24 | 0.09 | -0.01 | 0.01 | -0.01 | 0.03 | 0.02 | 0.01 | SEDEX |
| S-12 | -0.25 | 0.11 | -0.09 | 0.01 | 0.00 | 0.01 | -0.09 | 0.06 | SEDEX |
| S-13 | -0.81 | 0.21 | 0.12 | 0.02 | 0.01 | 0.01 | 0.09 | 0.03 | SEDEX |
| S-14 | -0.73 | 0.08 | 0.01 | 0.08 | 0.02 | 0.01 | 0.01 | 0.09 | SEDEX |
| S-15 | -0.24 | 0.15 | -0.07 | 0.09 | -0.01 | 0.01 | -0.1 | 0.06 | SEDEX |
| S-16 | -0.7 | 0.17 | 0.06 | 0.05 | 0.01 | 0.04 | 0.05 | 0.09 | SEDEX |
| S-17 | -1.03 | 0.17 | -0.03 | 0.04 | -0.01 | 0.04 | -0.06 | 0.01 | SEDEX |
| S-18 | -0.51 | 0.12 | 0.05 | 0.01 | 0.01 | 0.01 | 0.05 | 0.05 | SEDEX |
| S-19 | -0.33 | 0.08 | -0.03 | 0.05 | -0.02 | 0.01 | -0.04 | 0.06 | SEDEX |
| M-1 | 0.24 | 0.19 | 0.04 | 0.02 | 0.01 | 0.01 | 0.05 | 0.04 | MVT |
| M-2 | -1.02 | 0.1 | 0.12 | 0.01 | 0.02 | 0.03 | 0.09 | 0.03 | MVT |
| M-3 | -0.36 | 0.05 | 0.04 | 0.08 | 0.01 | 0.03 | 0.06 | 0.04 | MVT |
| M-4 | -0.63 | 0.09 | 0.11 | 0.04 | -0.02 | 0 | 0.13 | 0.01 | MVT |
| M-5 | -0.88 | 0.09 | -0.13 | 0.02 | 0.04 | 0.05 | -0.15 | 0 | MVT |
| M-6 | 0.19 | 0.08 | 0.02 | 0.07 | -0.01 | 0.01 | 0.05 | 0.03 | MVT |
| M-7 | -0.23 | 0.1 | 0.03 | 0.08 | 0.02 | 0.03 | 0.03 | 0.01 | MVT |
| M-8 | -0.66 | 0.05 | -0.05 | 0.08 | -0.01 | 0 | -0.05 | 0.04 | MVT |
| M-9 | -0.57 | 0.05 | 0.05 | 0.01 | 0.02 | 0.02 | 0.03 | 0 | MVT |
| M-10 | -1.87 | 0.04 | -0.09 | 0.04 | 0.02 | 0.04 | -0.08 | 0 | MVT |
| M-11 | -1.29 | 0.04 | 0 | 0.04 | 0.02 | 0.02 | 0.03 | 0.01 | MVT |
| M-12 | -0.96 | 0.14 | 0.04 | 0.02 | 0.02 | 0.01 | 0.08 | 0.03 | MVT |
| M-13 | -0.67 | 0.1 | 0.04 | 0.01 | -0.01 | 0.03 | 0.06 | 0.02 | MVT |
| M-14 | -1.43 | 0.13 | 0.09 | 0.01 | 0.02 | 0 | 0.09 | 0.04 | MVT |
| M-15 | -0.32 | 0.18 | 0.08 | 0.01 | 0.01 | 0.01 | 0.09 | 0.09 | MVT |
| M-16 | -0.29 | 0.18 | 0.14 | 0.01 | 0.01 | 0.02 | 0.16 | 0.02 | MVT |
| M-17 | -0.9 | 0.05 | 0.03 | 0.02 | 0.02 | 0.01 | 0.04 | 0.04 | MVT |
| M-18 | -1.3 | 0.15 | 0.09 | 0 | -0.01 | 0 | 0.05 | 0.02 | MVT |
| M-19 | -0.15 | 0.14 | 0.03 | 0 | 0.01 | 0.01 | 0.05 | 0.05 | MVT |
| M-20 | -1.13 | 0.21 | 0.08 | 0.01 | 0.01 | 0 | 0.03 | 0.01 | MVT |
| M-21 | -0.61 | 0.11 | 0.08 | 0.01 | 0.01 | 0.02 | 0.07 | 0.01 | MVT |
| M-22 | -1.25 | 0.05 | 0.05 | 0.03 | 0.02 | 0.04 | 0.02 | 0.06 | MVT |
| M-23 | -1.44 | 0.12 | -0.06 | 0.02 | 0.02 | 0 | 0.02 | 0.01 | MVT |
| M-24 | 0.61 | 0.05 | -0.21 | 0.02 | 0.02 | 0.02 | -0.19 | 0 | MVT |
| M-25 | 0.7 | 0.05 | -0.24 | 0.02 | 0.01 | 0.04 | -0.23 | 0 | MVT |
| V-1 | -0.72 | 0.14 | -0.01 | 0 | 0.02 | 0.04 | -0.11 | 0.1 | VMS |
| V-2 | -0.17 | 0.08 | 0.04 | 0.02 | 0.01 | 0.04 | -0.01 | 0.04 | VMS |
| V-3 | -0.28 | 0.07 | -0.06 | 0.03 | -0.01 | 0.03 | 0 | 0.07 | VMS |
| V-4 | -0.57 | 0.19 | -0.02 | 0.04 | -0.01 | 0.01 | -0.02 | 0.01 | VMS |
| V-5 | -0.71 | 0.12 | 0.03 | 0.01 | 0.01 | 0.01 | 0.01 | 0.07 | VMS |
| V-6 | -0.68 | 0.11 | 0.01 | 0.01 | 0.00 | 0.01 | 0.06 | 0.03 | VMS |
| V-7 | -0.72 | 0.17 | 0.05 | 0.06 | -0.02 | 0.05 | 0.05 | 0.01 | VMS |
| V-8 | -0.53 | 0.15 | 0.01 | 0.05 | 0.01 | 0.01 | -0.07 | 0.02 | VMS |
| V-9 | -0.26 | 0.35 | 0.03 | 0.02 | -0.01 | 0.01 | -0.04 | 0.13 | VMS |
| V-10 | -0.82 | 0.13 | 0.06 | 0.04 | -0.02 | 0.01 | -0.03 | 0.01 | VMS |
| V-11 | -0.13 | 0.16 | -0.03 | 0.01 | 0.01 | 0.01 | 0 | 0.06 | VMS |
| V-12 | -0.41 | 0.2 | -0.01 | 0.01 | 0.01 | 0.01 | 0.06 | 0.01 | VMS |
| V-13 | -0.4 | 0.09 | -0.06 | 0.01 | 0.01 | 0 | 0.02 | 0.09 | VMS |
| V-14 | -0.84 | 0.04 | 0.04 | 0.03 | 0.02 | 0 | 0.04 | 0.03 | VMS |
| IR-1 | -0.19 | 0.15 | -0.04 | 0.01 | 0.01 | 0.01 | -0.03 | 0.1 | IR |
| IR-2 | 0.36 | 0.14 | 0.05 | 0.01 | 0.01 | 0.01 | 0.05 | 0.06 | IR |
| IR-3 | -0.58 | 0.11 | 0.04 | 0.1 | 0.02 | 0.03 | 0.03 | 0.07 | IR |
| IR-4 | 0.12 | 0.14 | -0.03 | 0.04 | -0.01 | 0.02 | 0.03 | 0.01 | IR |
| IR-5 | 0.24 | 0.13 | 0.05 | 0 | 0.02 | 0 | 0.01 | 0.04 | IR |
| IR-6 | 0.16 | 0.04 | 0.05 | 0.05 | 0.01 | 0.03 | 0.05 | 0.01 | IR |
| IR-7 | -0.36 | 0.12 | -0.01 | 0 | 0.02 | 0.02 | 0.06 | 0.02 | IR |
| IR-8 | -0.05 | 0.04 | 0.03 | 0.05 | 0.01 | 0.02 | 0.02 | 0.01 | IR |
| IR-9 | -0.07 | 0.15 | -0.05 | 0.01 | -0.02 | 0.03 | -0.07 | 0.02 | IR |
| IR-10 | -0.54 | 0.19 | -0.05 | 0.04 | 0.02 | 0.01 | -0.07 | 0.02 | IR |
| IR-11 | -0.21 | 0.12 | 0.04 | 0.04 | 0.02 | 0.02 | -0.02 | 0.07 | IR |
| IR-12 | -0.07 | 0.13 | -0.05 | 0.02 | 0.01 | 0.01 | 0.04 | 0.03 | IR |
| IR-13 | -0.66 | 0.05 | 0.04 | 0 | 0.02 | 0.04 | -0.01 | 0.06 | IR |
| IR-14 | -0.25 | 0.08 | 0.06 | 0 | -0.02 | 0.04 | 0.04 | 0.04 | IR |
| IR-15 | -0.28 | 0.15 | -0.05 | 0.07 | -0.01 | 0.01 | -0.08 | 0.04 | IR |
| IR-16 | -0.96 | 0.05 | 0.05 | 0.02 | -0.01 | 0.01 | 0.04 | 0.02 | IR |
| IR-17 | -0.28 | 0.16 | 0.07 | 0.03 | -0.01 | 0.01 | 0.02 | 0.03 | IR |
| IR-18 | 0.13 | 0.09 | 0.02 | 0.01 | 0.02 | 0.03 | 0.03 | 0.02 | IR |
| IR-19 | 0.21 | 0.14 | 0.07 | 0.02 | 0.01 | 0.01 | 0.03 | 0.01 | IR |
| IR-20 | -0.4 | 0.11 | 0.04 | 0.04 | -0.03 | 0.01 | 0.03 | 0.03 | IR |
| IR-21 | -0.72 | 0.28 | 0.04 | 0.01 | -0.02 | 0.01 | 0.03 | 0.06 | IR |
| IR-22 | -0.08 | 0.14 | -0.01 | 0.03 | -0.01 | 0.01 | 0.07 | 0.01 | IR |
| IR-23 | -0.44 | 0.13 | 0.06 | 0 | 0.02 | 0.01 | 0.03 | 0.01 | IR |
| IR-24 | -0.28 | 0.07 | -0.03 | 0.07 | -0.01 | 0.01 | 0.02 | 0.01 | IR |
| IR-25 | -0.66 | 0.13 | 0.07 | 0.01 | 0.02 | 0.01 | 0.06 | 0.09 | IR |
| IR-26 | -0.67 | 0.18 | 0.02 | 0.01 | 0.01 | 0.06 | -0.02 | 0.01 | IR |
| IR-27 | -0.71 | 0.04 | 0.05 | 0.02 | -0.02 | 0.01 | 0.08 | 0.01 | IR |
| IR-28 | -0.32 | 0.12 | 0.02 | 0.01 | -0.02 | 0.04 | -0.07 | 0.01 | IR |
| IR-29 | -0.66 | 0.17 | 0.06 | 0.06 | -0.01 | 0.05 | 0.04 | 0.03 | IR |
| IR-30 | -0.7 | 0.24 | 0.02 | 0.02 | 0.01 | 0 | -0.03 | 0.01 | IR |
| IR-31 | 0.48 | 0.1 | -0.06 | 0.02 | -0.01 | 0.01 | 0.03 | 0.06 | IR |
| IR-32 | -0.62 | 0.09 | -0.02 | 0.01 | 0.01 | 0.01 | 0.01 | 0.07 | IR |
| IR-33 | -0.61 | 0.06 | 0.01 | 0.03 | 0.02 | 0.01 | 0.01 | 0.04 | IR |
| IR-34 | 0.54 | 0.09 | -0.01 | 0.01 | 0.02 | 0.03 | 0.08 | 0.02 | IR |
| IR-35 | -0.33 | 0.11 | 0.04 | 0.01 | 0.01 | 0.04 | 0.01 | 0.01 | IR |
| IR-36 | 0.18 | 0.11 | 0.05 | 0.02 | 0.02 | 0.01 | -0.02 | 0.08 | IR |
| IR-37 | -0.73 | 0.12 | 0.01 | 0.1 | 0.02 | 0.01 | -0.05 | 0.06 | IR |
| IR-38 | -0.35 | 0.11 | -0.01 | 0.04 | 0.02 | 0.04 | 0.03 | 0.06 | IR |
| IR-39 | -0.54 | 0.09 | 0.02 | 0 | 0.00 | 0.01 | -0.1 | 0.08 | IR |
| IR-40 | -0.42 | 0.1 | 0.05 | 0 | 0.01 | 0.04 | 0.06 | 0.06 | IR |
| IR-41 | -0.74 | 0.15 | 0.03 | 0.01 | 0.01 | 0.03 | 0.08 | 0 | IR |
| IR-42 | -0.71 | 0.08 | -0.03 | 0.01 | 0.01 | 0.01 | 0.01 | 0.08 | IR |
| IR-43 | -0.34 | 0.04 | 0.02 | 0.01 | 0.02 | 0.04 | 0.05 | 0.01 | IR |
| IR-44 | -0.84 | 0.11 | -0.07 | 0.01 | 0.02 | 0.01 | -0.07 | 0.02 | IR |

**Table S3 Sample locations, THg, Zn reserve and ore genesis of the sphalerites in China**

| **Deposit No.** | **Deposit Name** | **Locations** | **Provinces** | **HgT** | **Zn** | **Deposit** | **Additional references** |
| --- | --- | --- | --- | --- | --- | --- | --- |
|  |  |  |  | **(**μg/g**)** | **(10^4^t)** | **Types** |  |
| S-1 | Jiashengpan | Wulate | Neimenggu | 5.49 | 126.85 | SEDEX | Dai et al., 2005; Yin et al., 2012 |
| S-2 | Dongshengmiao | Wulate | Neimenggu | 7.03 | 408.24 | SEDEX | Dai et al., 2005; Yin et al., 2012 |
| S-3 | Tanyaokou | Wulate | Neimenggu | 3.16 | 110.66 | SEDEX | Dai et al., 2005; Yin et al., 2012 |
| S-4 | Dabaoshan | Qujiang | Guangdong | 5.17 | 69.79 | SEDEX | Dai et al., 2005; Yin et al., 2012 |
| S-5 | Jinding | Lanping | Yunnan | 29.78 | 1347.07 | SEDEX | Dai et al., 2005; Yin et al., 2012 |
| S-6 | Qiandongshan | Fengxian | Shanxi | 71.9 | 91.81 | SEDEX | Dai et al., 2005; Yin et al., 2012 |
| S-7 | Bafangshan | Fengxian | Shanxi | 9.31 | 39.74 | SEDEX | Dai et al., 2005; Yin et al., 2012 |
| S-8 | Yinmusi | Fengxian | Shanxi | 141 | 21.43 | SEDEX | Dai et al., 2005; Yin et al., 2012 |
| S-9 | Fengya | Fengxian | Shanxi | 91 | 28.46 | SEDEX | Dai et al., 2005; Yin et al., 2012 |
| S-10 | Erlihe | Fengxian | Shanxi | 191 | 27.33 | SEDEX | Dai et al., 2005; Yin et al., 2012 |
| S-11 | Yindongliang | Fengxian | Shanxi | 89 | 32.99 | SEDEX | Dai et al., 2005; Yin et al., 2012 |
| S-12 | Yindongzi | Zuoshui | Shanxi | 5.59 | 0.99 | SEDEX | Dai et al., 2005; Yin et al., 2012 |
| S-13 | Changba | Chengxian | Gansu | 195 | 306.44 | SEDEX | Dai et al., 2005; Yin et al., 2012 |
| S-14 | Lijiagou | Chengxian | Gansu | 300 | 164.41 | SEDEX | Dai et al., 2005; Yin et al., 2012 |
| S-15 | Bijiashan | Chengxian | Gansu | 91.7 | 45.92 | SEDEX | Dai et al., 2005; Yin et al., 2012 |
| S-16 | Dengjiashan | Xihe | Gansu | 210 | 46.49 | SEDEX | Dai et al., 2005; Yin et al., 2012 |
| S-17 | Luoba | Huixian | Gansu | 323 | 88.1 | SEDEX | Dai et al., 2005; Yin et al., 2012 |
| S-18 | Jiaolongzhang | Langzhuang | Gansu | 72.37 | 13.79 | SEDEX | Dai et al., 2005; Yin et al., 2012 |
| S-19 | Fankou | Renhua | Guangdong | 237 | 549.28 | SEDEX | Dai et al., 2005; Yin et al., 2012 |
| V-1 | Hongtoushan | Qingyuan | Liaoning | 179.7 | 68.84 | VMS | Dai et al., 2005; Yin et al., 2012 |
| V-2 | Wuao | Longquan | Zhejiang | 10.74 | 16.74 | VMS | Dai et al., 2005; Yin et al., 2012 |
| V-3 | Qiwan | Zhuji city | Zhejiang | 111.6 | 11.4 | VMS | Dai et al., 2005; Yin et al., 2012 |
| V-4 | Shuiji | Jianyang | Fujian | 2.97 | 24.15 | VMS | Dai et al., 2005; Yin et al., 2012 |
| V-5 | Meixian | Youxi | Fujian | 11.25 | 87.44 | VMS | Dai et al., 2005; Yin et al., 2012 |
| V-6 | Jiacun | Baiyu | Sichuan | 2.28 | 86.73 | VMS | Dai et al., 2005; Yin et al., 2012 |
| V-7 | Gayiqiong | Baiyu | Sichuan | 10.47 | 31.33 | VMS | Dai et al., 2005; Yin et al., 2012 |
| V-8 | Xiaotieshan | Baiyin | Gansu | 117.6 | 64.18 | VMS | Dai et al., 2005; Yin et al., 2012 |
| V-9 | Xitieshan | Chaidamu | Qinghai | 96 | 181.72 | VMS | Dai et al., 2005; Yin et al., 2012 |
| V-10 | Zhaokalong | Yushu | Qinghai | 4.47 | 2.27 | VMS | Dai et al., 2005; Yin et al., 2012 |
| V-11 | Ashele | Habahe | Xinjiang | 1.83 | 43.8 | VMS | Dai et al., 2005; Yin et al., 2012 |
| V-12 | Tiemierteduo | Aletai | Xinjiang | 0.93 | 11 | VMS | Dai et al., 2005; Yin et al., 2012 |
| V-13 | Abagong | Aletai | Xinjiang | 75 | 15.26 | VMS | Dai et al., 2005; Yin et al., 2012 |
| V-14 | Laochang | Lanchang | Yunnan | 0.63 | 32.86 | VMS | Dai et al., 2005; Yin et al., 2012 |
| M-1 | Chaihe | Kaiyuan | Liaoning | 1050 | 39.15 | MVT | Dai et al., 2005; Yin et al., 2012 |
| M-2 | Qingchengzi | Fengcheng | Liaoning | 10 | 34.93 | MVT | Dai et al., 2005; Yin et al., 2012 |
| M-3 | Qixiashan | Nanjing | Jiangsu | 2.61 | 75.5 | MVT | Dai et al., 2005; Yin et al., 2012 |
| M-4 | Ganjiaxiang | Nanjing | Jiangsu | 7.02 | 37.06 | MVT | Dai et al., 2005; Yin et al., 2012 |
| M-5 | Yinshan | Yangxin | Hubei | 0.76 | 35.49 | MVT | Dai et al., 2005; Yin et al., 2012 |
| M-6 | Limei | Huayuan | Hunan | 17.6 | 153.26 | MVT | Dai et al., 2005; Yin et al., 2012 |
| M-7 | Chaiqing | Lengshuijiang | Hunan | 12.1 | 35.83 | MVT | Dai et al., 2005; Yin et al., 2012 |
| M-8 | Houjiangqiao | Daoxian | Hunan | 2.13 | 50.8 | MVT | Dai et al., 2005; Yin et al., 2012 |
| M-9 | Beishan | Huanjiang | Guangxi | 77.4 | 103.33 | MVT | Dai et al., 2005; Yin et al., 2012 |
| M-10 | Siding | Rongan | Guangxi | 8.51 | 44.19 | MVT | Dai et al., 2005; Yin et al., 2012 |
| M-11 | Laochang | Yangshuo | Guangxi | 10 | 14.3 | MVT | Dai et al., 2005; Yin et al., 2012 |
| M-12 | Guli | Wuxuan | Guangxi | 21.6 | 9.86 | MVT | Dai et al., 2005; Yin et al., 2012 |
| M-13 | Zhaiziping | Kangding | Sichuan | 127 | 26.61 | MVT | Dai et al., 2005; Yin et al., 2012 |
| M-14 | Tianbanshan | Huili | Sichuan | 2.11 | 94.14 | MVT | Dai et al., 2005; Yin et al., 2012 |
| M-15 | Xiaoshifang | Huili | Sichuan | 44.77 | 29.73 | MVT | Dai et al., 2005; Yin et al., 2012 |
| M-16 | Daliangzi | Huidong | Sichuan | 41.82 | 200.72 | MVT | Dai et al., 2005; Yin et al., 2012 |
| M-17 | Zhazichang | Hezhang | Guizhou | 2.13 | 13.36 | MVT | Dai et al., 2005; Yin et al., 2012 |
| M-18 | 701 | Huize | Yunnan | 7.92 | 20.8 | MVT | Dai et al., 2005; Yin et al., 2012 |
| M-19 | Kuangshanchang | Huize | Yunnan | 25.2 | 49.6 | MVT | Dai et al., 2005; Yin et al., 2012 |
| M-20 | Wuxingchang | Huize | Yunnan | 9.71 | 9.89 | MVT | Dai et al., 2005; Yin et al., 2012 |
| M-21 | Fule | Luoping | Yunnan | 5.15 | 27.91 | MVT | Dai et al., 2005; Yin et al., 2012 |
| M-22 | Bainiuchang | Mengzi | Yunnan | 0.45 | 68.74 | MVT | Dai et al., 2005; Yin et al., 2012 |
| M-23 | Dulong | Maguan | Yunnan | 2.78 | 241.93 | MVT | Dai et al., 2005; Yin et al., 2012 |
| M-24 | Lanuoma | Changdu | Tibet | 6.32 | 48.85* | MVT | Tao et al., 2011; this study |
| M-25 | Zaxikang | Cuona Co. | Tibet | 4.11 | 25.82* | MVT | Lin et al., 2013; this study |
| IR-1 | Pingfeng | Pucheng | Fujian | 0.1 | 19.49 | IR | Dai et al., 2005; Yin et al., 2012 |
| IR-2 | Cuiqian | Gaoan | Jiangxi | 0.14 | 24.54 | IR | Dai et al., 2005; Yin et al., 2012 |
| IR-3 | Jianai | Datian | Fujian | 0.15 | 14.91 | IR | Dai et al., 2005; Yin et al., 2012 |
| IR-4 | Yinkeng | Putian | Fujian | 0.15 | 16.69 | IR | Dai et al., 2005; Yin et al., 2012 |
| IR-5 | Shangcang | Longmen | Guangdong | 0.21 | 22.12 | IR | Dai et al., 2005; Yin et al., 2012 |
| IR-6 | Yuli | Wuxian | Jiangsu | 0.24 | 24.18 | IR | Dai et al., 2005; Yin et al., 2012 |
| IR-7 | Qibaoshan | Shanggao | Jiangxi | 0.28 | 26.32 | IR | Dai et al., 2005; Yin et al., 2012 |
| IR-8 | Dachang | Nandan | Guangxi | 0.32 | 318.44 | IR | Dai et al., 2005; Yin et al., 2012 |
| IR-9 | Yindong | Fuding | Fujian | 0.32 | 24.9 | IR | Dai et al., 2005; Yin et al., 2012 |
| IR-10 | Huangshaping | Guiyang | Hunan | 0.34 | 110.8 | IR | Dai et al., 2005; Yin et al., 2012 |
| IR-11 | Shuikoushan | Changning | Hunan | 0.36 | 111.08 | IR | Dai et al., 2005; Yin et al., 2012 |
| IR-12 | Caijiayingzi | Zhangbei | Hebei | 0.39 | 143.96 | IR | Dai et al., 2005; Yin et al., 2012 |
| IR-13 | Chaipai | Longmen | Guangdong | 0.44 | 34.63 | IR | Dai et al., 2005; Yin et al., 2012 |
| IR-14 | Lengshuikeng | Guixi | Jiangxi | 0.46 | 218.82 | IR | Dai et al., 2005; Yin et al., 2012 |
| IR-15 | Xiertala | Chenbaerhu | Neimenggu | 0.59 | 27.67 | IR | Dai et al., 2005; Yin et al., 2012 |
| IR-16 | Lame | Nandan | Guangxi | 0.73 | 56.51 | IR | Dai et al., 2005; Yin et al., 2012 |
| IR-17 | Qibaoshan | Liuyang | Hunan | 0.95 | 21.64 | IR | Dai et al., 2005; Yin et al., 2012 |
| IR-18 | Sanhe | Eergunazuoqi | Neimenggu | 1.31 | 15.73 | IR | Dai et al., 2005; Yin et al., 2012 |
| IR-19 | Wangjiazhuang | Fushan | Shandong | 1.55 | 22.38 | IR | Dai et al., 2005; Yin et al., 2012 |
| IR-20 | Xiangkuang | Qixia | Shandong | 1.55 | 15.34 | IR | Dai et al., 2005; Yin et al., 2012 |
| IR-21 | Huanren | Huanren | Liaoning | 1.59 | 49.59 | IR | Dai et al., 2005; Yin et al., 2012 |
| IR-22 | Jiaoli | Shangyou | Jiangxi | 1.59 | 1.01 | IR | Dai et al., 2005; Yin et al., 2012 |
| IR-23 | Haobugao | Balin | Neimenggu | 1.88 | 62.46 | IR | Dai et al., 2005; Yin et al., 2012 |
| IR-24 | Congshuban | Chenzhou | Hunan | 2.13 | 45.32 | IR | Dai et al., 2005; Yin et al., 2012 |
| IR-25 | Wubu | Huangyan | Zhejiang | 2.55 | 88.28 | IR | Dai et al., 2005; Yin et al., 2012 |
| IR-26 | Tamu | Yingjisha | Xinjiang | 3.11 | 13.43 | IR | Dai et al., 2005; Yin et al., 2012 |
| IR-27 | Gejiu | Gejiu | Yunnan | 3.27 | 53 | IR | Dai et al., 2005; Yin et al., 2012 |
| IR-28 | Huoshibulake | Atushi | Xinjiang | 3.49 | 3.69 | IR | Dai et al., 2005; Yin et al., 2012 |
| IR-29 | Baiyinnuo | Balin | Neimenggu | 4.08 | 196.04 | IR | Dai et al., 2005; Yin et al., 2012 |
| IR-30 | Yueshan | Lujiang | Anhui | 4.1 | 27.56 | IR | Dai et al., 2005; Yin et al., 2012 |
| IR-31 | Taolin | Linxiang | Hunan | 7 | 65.32 | IR | Dai et al., 2005; Yin et al., 2012 |
| IR-32 | Xiaoxilin | Yichun | Heilongjiang | 7 | 53 | IR | Dai et al., 2005; Yin et al., 2012 |
| IR-33 | Changtun | Daxin | Guangxi | 7.04 | 22.73 | IR | Dai et al., 2005; Yin et al., 2012 |
| IR-34 | Jiawula | Chenbaerhu | Neimenggu | 8.8 | 37.9 | IR | Dai et al., 2005; Yin et al., 2012 |
| IR-35 | Fangniugou | Yitong | Jilin | 10 | 35.7 | IR | Dai et al., 2005; Yin et al., 2012 |
| IR-36 | Quli | Lushi | Henan | 13.54 | 68.47 | IR | Dai et al., 2005; Yin et al., 2012 |
| IR-37 | Xialadi | Lincang | Gansu | 30.8 | 0.08 | IR | Dai et al., 2005; Yin et al., 2012 |
| IR-38 | Qingshuitang | Qidong | Hunan | 41.04 | 24.2 | IR | Dai et al., 2005; Yin et al., 2012 |
| IR-39 | Bajiazi | Jianchang | Liaoning | 50 | 22.59 | IR | Dai et al., 2005; Yin et al., 2012 |
| IR-40 | Shiduolong | Xinghai | Qinghai | 50 | 11.11 | IR | Dai et al., 2005; Yin et al., 2012 |
| IR-41 | Xiaoyingzi | Wengniute | Neimenggu | 67.3 | 20.48 | IR | Dai et al., 2005; Yin et al., 2012 |
| IR-42 | Dajianshan | Lianping | Guangdong | 81.6 | 27.81 | IR | Dai et al., 2005; Yin et al., 2012 |
| IR-43 | Fozichong | Qinxi | Guangxi | 200 | 33.16 | IR | Dai et al., 2005; Yin et al., 2012 |
| IR-44 | Yinshan | Dexing | Jiangxi | 308.41 | 44.34 | IR | Dai et al., 2005; Yin et al., 2012 |

*data source: <http://dzzl.mlr.gov.cn/xzdatagateway/datasetView.action?datasetVO.serverNodeVO.id=1&datasetVO.lid=2102>

**References**

Bergquist, B. A., & Blum, J. D. (2007). Mass-dependent and-independent fractionation of Hg isotopes by photoreduction in aquatic systems. Science, 318(5849), 417-420.

Biswas, A., Blum, J. D., Bergquist, B. A., Keeler, G. J., & Xie, Z. (2008). Natural mercury isotope variation in coal deposits and organic soils. Environmental Science & Technology, 42(22), 8303-8309.

Blum, J. D., Johnson, M. W., Gleason, J. D., Demers, J. D., Landis, M. S., & Krupa, S. (2012). Mercury concentration and isotopic composition of epiphytic tree lichens in the Athabasca Oil Sands Region. Alberta Oil Sands: Energy, Industry and the Environment: Elsevier Press, Oxford, UK, 373-390.

Blum, J. D., Popp, B. N., Drazen, J. C., Choy, C. A., & Johnson, M. W. (2013). Methylmercury production below the mixed layer in the North Pacific Ocean. Nature Geoscience, 6(10), 879-884.

Carignan, J., Estrade, N., Sonke, J. E., & Donard, O. F. (2009). Odd isotope deficits in atmospheric Hg measured in lichens. Environmental science & technology, 43(15), 5660-5664.

Chen, J., Hintelmann, H., Feng, X., & Dimock, B. (2012). Unusual fractionation of both odd and even mercury isotopes in precipitation from Peterborough, ON, Canada. Geochimica et Cosmochimica Acta, 90, 33-46.

Cooke, C. A., Hintelmann, H., Ague, J. J., Burger, R., Biester, H., Sachs, J. P., & Engstrom, D. R. (2013). Use and Legacy of Mercury in the Andes. Environmental science & technology, 47(9), 4181-4188.

Demers J D, Blum J D, Zak D R. Mercury isotopes in a forested ecosystem: Implications for air-surface exchange dynamics and the global mercury cycle. Global Biogeochemical Cycles, 2013, 27(1): 222-238.

Donovan, P. M., Blum, J. D., Yee, D., Gehrke, G. E., & Singer, M. B. (2013). An isotopic record of mercury in San Francisco Bay sediment. Chemical Geology, 349, 87-98.

Estrade, N., Carignan, J., & Donard, O. F. (2010). Isotope tracing of atmospheric mercury sources in an urban area of northeastern France. Environmental science & technology, 44(16), 6062-6067.

Estrade, N., Carignan, J., & Donard, O. F. (2011). Tracing and quantifying anthropogenic mercury sources in soils of northern France using isotopic signatures. Environmental science & technology, 45(4), 1235-1242.

Feng, X., Yin, R., Yu, B., & Du, B. (2013). Mercury isotope variations in surface soils in different contaminated areas in Guizhou Province, China. Chinese Science Bulletin, 58(2), 249-255.

Foucher, D., & Hintelmann, H. (2008). Tracing mercury contamination from the Idrija mining region (Slovenia) to the Gulf of Trieste using Hg isotope ratio measurements. Environmental Science & Technology, 43(1), 33-39.

Foucher, D., Hintelmann, H., Al, T. A., & MacQuarrie, K. T. (2013). Mercury isotope fractionation in waters and sediments of the Murray Brook mine watershed (New Brunswick, Canada): Tracing mercury contamination and transformation. Chemical Geology, 336, 87-95.

Gantner, N., Hintelmann, H., Zheng, W., & Muir, D. C. (2009). Variations in stable isotope fractionation of Hg in food webs of Arctic lakes. Environmental science & technology, 43(24), 9148-9154.

Gehrke, G. E., Blum, J. D., & Marvin-DiPasquale, M. (2011). Sources of mercury to San Francisco Bay surface sediment as revealed by mercury stable isotopes. Geochimica et Cosmochimica Acta, 75(3), 691-705.

Gehrke, G. E., Blum, J. D., & Meyers, P. A. (2009). The geochemical behavior and isotopic composition of Hg in a mid-Pleistocene western Mediterranean sapropel. Geochimica et Cosmochimica Acta, 73(6), 1651-1665.

Gehrke, G. E., Blum, J. D., Slotton, D. G., & Greenfield, B. K. (2011). Mercury isotopes link mercury in San Francisco Bay forage fish to surface sediments. Environmental science & technology, 45(4), 1264-1270.

Ghosh, S., Xu, Y., Humayun, M., & Odom, L. (2008). Mass‐independent fractionation of mercury isotopes in the environment. Geochemistry, Geophysics, Geosystems, 9(3).

Gratz, L. E., Keeler, G. J., Blum, J. D., & Sherman, L. S. (2010). Isotopic composition and fractionation of mercury in Great Lakes precipitation and ambient air. Environmental science & technology, 44(20), 7764-7770.

Kwon, S. Y., Blum, J. D., Carvan, M. J., Basu, N., Head, J. A., Madenjian, C. P., & David, S. R. (2012). Absence of fractionation of mercury isotopes during trophic transfer of methylmercury to freshwater fish in captivity. Environmental science & technology, 46(14), 7527-7534.

Kwon, S. Y., Blum, J. D., Chen, C. Y., Meattey, D. E., & Mason, R. P. (2014). Mercury isotope study of sources and exposure pathways of methylmercury in estuarine food webs in the Northeastern US. Environmental science & technology, 48(17), 10089-10097.

Kwon, S. Y., Blum, J. D., Chirby, M. A., & Chesney, E. J. (2013). Application of mercury isotopes for tracing trophic transfer and internal distribution of mercury in marine fish feeding experiments. Environmental Toxicology and Chemistry, 32(10), 2322-2330.

Laffont, L., Sonke, J. E., Maurice, L., Hintelmann, H., Pouilly, M., Sánchez Bacarreza, Y., ... & Behra, P. (2009). Anomalous mercury isotopic compositions of fish and human hair in the Bolivian Amazon. Environmental science & technology, 43(23), 8985-8990.

Laffont, L., Sonke, J. E., Maurice, L., Monrroy, S. L., Chincheros, J., Amouroux, D., & Behra, P. (2011). Hg speciation and stable isotope signatures in human hair as a tracer for dietary and occupational exposure to mercury. Environmental science & technology, 45(23), 9910-9916.

Lefticariu, L., Blum, J. D., & Gleason, J. D. (2011). Mercury isotopic evidence for multiple mercury sources in coal from the Illinois Basin. Environmental science & technology, 45(4), 1724-1729.

Lin, B.; Tang, J.; Zheng, W.; et al. Geological characteristics and modes of occurrence of silver in Zhaxikang zinc polymetallic deposit. Mineral Deposits, 2013, 32(5), 899-914 (in Chinese with English abstract

Liu, J., Feng, X., Yin, R., Zhu, W., & Li, Z. (2011). Mercury distributions and mercury isotope signatures in sediments of Dongjiang, the Pearl River Delta, China. Chemical Geology, 287(1), 81-89.

Mil-Homens, M., Blum, J., Canário, J., Caetano, M., Costa, A. M., Lebreiro, S. M., ... & Melo, Z. (2013). Tracing anthropogenic Hg and Pb input using stable Hg and Pb isotope ratios in sediments of the central Portuguese Margin. Chemical Geology, 336, 62-71.

Perrot, V., Epov, V. N., Pastukhov, M. V., Grebenshchikova, V. I., Zouiten, C., Sonke, J. E., ... & Amouroux, D. (2010). Tracing sources and bioaccumulation of mercury in fish of Lake Baikal− Angara River using Hg isotopic composition. Environmental science & technology, 44(21), 8030-8037.

Perrot, V., Pastukhov, M. V., Epov, V. N., Husted, S., Donard, O. F., & Amouroux, D. (2012). Higher mass-independent isotope fractionation of methylmercury in the pelagic food web of Lake Baikal (Russia). Environmental science & technology, 46(11), 5902-5911.

Point, D., Sonke, J. E., Day, R. D., Roseneau, D. G., Hobson, K. A., Vander Pol, S. S., ... & Becker, P. R. (2011). Methylmercury photodegradation influenced by sea-ice cover in Arctic marine ecosystems. Nature geoscience, 4(3), 188-194.

Rolison, J. M., Landing, W. M., Luke, W., Cohen, M., & Salters, V. J. M. (2013). Isotopic composition of species-specific atmospheric Hg in a coastal environment. Chemical Geology, 336, 37-49.

Senn, D. B., Chesney, E. J., Blum, J. D., Bank, M. S., Maage, A., & Shine, J. P. (2010). Stable isotope (N, C, Hg) study of methylmercury sources and trophic transfer in the northern Gulf of Mexico. Environmental science & technology, 44(5), 1630-1637.

Sherman, L. S., & Blum, J. D. (2013). Mercury stable isotopes in sediments and largemouth bass from Florida lakes, USA. Science of the Total Environment, 448, 163-175.

Sherman, L. S., Blum, J. D., Douglas, T. A., & Steffen, A. (2012). Frost flowers growing in the Arctic ocean‐atmosphere–sea ice–snow interface: 2. Mercury exchange between the atmosphere, snow, and frost flowers. Journal of Geophysical Research: Atmospheres (1984–2012), 117(D14).

Sherman, L. S., Blum, J. D., Johnson, K. P., Keeler, G. J., Barres, J. A., & Douglas, T. A. (2010). Mass-independent fractionation of mercury isotopes in Arctic snow driven by sunlight. Nature Geoscience, 3(3), 173-177.

Sherman, L. S., Blum, J. D., Keeler, G. J., Demers, J. D., & Dvonch, J. T. (2011). Investigation of local mercury deposition from a coal-fired power plant using mercury isotopes. Environmental science & technology, 46(1), 382-390.

Sherman, L. S., Blum, J. D., Nordstrom, D. K., McCleskey, R. B., Barkay, T., & Vetriani, C. (2009). Mercury isotopic composition of hydrothermal systems in the Yellowstone Plateau volcanic field and Guaymas Basin sea-floor rift. Earth and Planetary Science Letters, 279(1), 86-96.

Smith, C. N., Kesler, S. E., Blum, J. D., & Rytuba, J. J. (2008). Isotope geochemistry of mercury in source rocks, mineral deposits and spring deposits of the California Coast Ranges, USA. Earth and Planetary Science Letters, 269(3), 399-407.

Smith, C. N., Kesler, S. E., Klaue, B., & Blum, J. D. (2005). Mercury isotope fractionation in fossil hydrothermal systems. Geology, 33(10), 825-828.

Sonke, J. E., Schäfer, J., Chmeleff, J., Audry, S., Blanc, G., & Dupré, B. (2010). Sedimentary mercury stable isotope records of atmospheric and riverine pollution from two major European heavy metal refineries. Chemical Geology, 279(3), 90-100.

Stetson, S. J., Gray, J. E., Wanty, R. B., & Macalady, D. L. (2009). Isotopic variability of mercury in ore, mine-waste calcine, and leachates of mine-waste calcine from areas mined for mercury. Environmental science & technology, 43(19), 7331-7336.

Sun, R., Heimbürger, L. E., Sonke, J. E., Liu, G., Amouroux, D., & Berail, S. (2013). Mercury stable isotope fractionation in six utility boilers of two large coal-fired power plants. Chemical Geology, 336, 103-111.

Sun, R., Sonke, J. E., Liu, G., Zheng, L., & Wu, D. (2014). Variations in the stable isotope composition of mercury in coal-bearing sequences: Indications for its provenance and geochemical processes. International Journal of Coal Geology, 133, 13-23.

Sun, R., Sonke, J., Heimbürger, L. E., Belkin, H., Liu, G., Shome, D., ... & Streets, D. G. (2014). Mercury Stable Isotope Signatures of World Coal Deposits and Historical Coal Combustion Emissions. Environmental Science & Technology.

Tao Y., Bi, X., Xin, Z., et al. (2011): Geology, geochemistry and origin of Lanuoma Pb-Zn-Sb deposit in Changdu area, Tibet. Mineral Deposits 30(4), 599-615 (in Chinese with English abstract).

Tsui, M. T. K., Blum, J. D., Finlay, J. C., Balogh, S. J., Nollet, Y. H., Palen, W. J., & Power, M. E. (2014). Variation in terrestrial and aquatic sources of methylmercury in stream predators as revealed by stable mercury isotopes. Environmental science & technology, 48(17), 10128-10135.

Tsui, M. T. K., Blum, J. D., Kwon, S. Y., Finlay, J. C., Balogh, S. J., & Nollet, Y. H. (2012). Sources and transfers of methylmercury in adjacent river and forest food webs. Environmental science & technology, 46(20), 10957-10964.

Wiederhold, J. G., Smith, R. S., Siebner, H., Jew, A. D., Brown Jr, G. E., Bourdon, B., & Kretzschmar, R. (2013). Mercury Isotope Signatures as Tracers for Hg Cycling at the New Idria Hg Mine. Environmental science & technology, 47(12), 6137-6145.

Yin R.S., Feng X.B., Zhang J.J. et al., (2014) Identifying the Sources and Processes of Mercury in Subtropical Estuarine and Ocean Sediments using Hg Isotopic Composition. Submitted to Environmental science & technology.

Yin, R., Feng, X., & Chen, J. (2014). Mercury Stable Isotope Compositions in Coals from Major Coal Producing Fields in China and Their Geochemical and Environmental Implications. Environmental science & technology.

Yin, R., Feng, X., & Meng, B. (2013). Stable mercury isotope variation in rice plants (Oryza sativa L.) from the Wanshan mercury mining district, SW China. Environmental science & technology, 47(5), 2238-2245.

Yin, R., Feng, X., Wang, J., Bao, Z., Yu, B., & Chen, J. (2013). Mercury isotope variations between bioavailable mercury fractions and total mercury in mercury contaminated soil in Wanshan Mercury Mine, SW China. Chemical Geology, 336, 80-86.

Yin, R., Feng, X., Wang, J., Li, P., Liu, J., Zhang, Y., Hu, T. (2013). Mercury speciation and mercury isotope fractionation during ore roasting process and their implication to source identification of downstream sediment in the Wanshan mercury mining area, SW China. Chemical Geology, 336, 72-79.

Yin R, Feng X, Li Z, et al. (2012). Metallogeny and environmental impact of Hg in Zn deposits in China. Applied Geochemistry, 27(1): 151-160.

Zambardi, T., Sonke, J. E., Toutain, J. P., Sortino, F., & Shinohara, H. (2009). Mercury emissions and stable isotopic compositions at Vulcano Island (Italy). Earth and Planetary Science Letters, 277(1), 236-243.

Zhang, H., Yin, R. S., Feng, X. B., Sommar, J., Anderson, C. W., Sapkota, A., ... & Larssen, T. (2013). Atmospheric mercury inputs in montane soils increase with elevation: evidence from mercury isotope signatures. Scientific reports, 3.
